# Supplementary figures and images for: Prevalence of CMV, EBV, HPV, and HSV among South Asian healthy population: A systematic review and meta-analysis
Source: PLOS Glob Public Health. 2026 Jan 7;6(1):e0005728. doi: 10.1371/journal.pgph.0005728 (PMC12779128; doi:10.1371/journal.pgph.0005728)

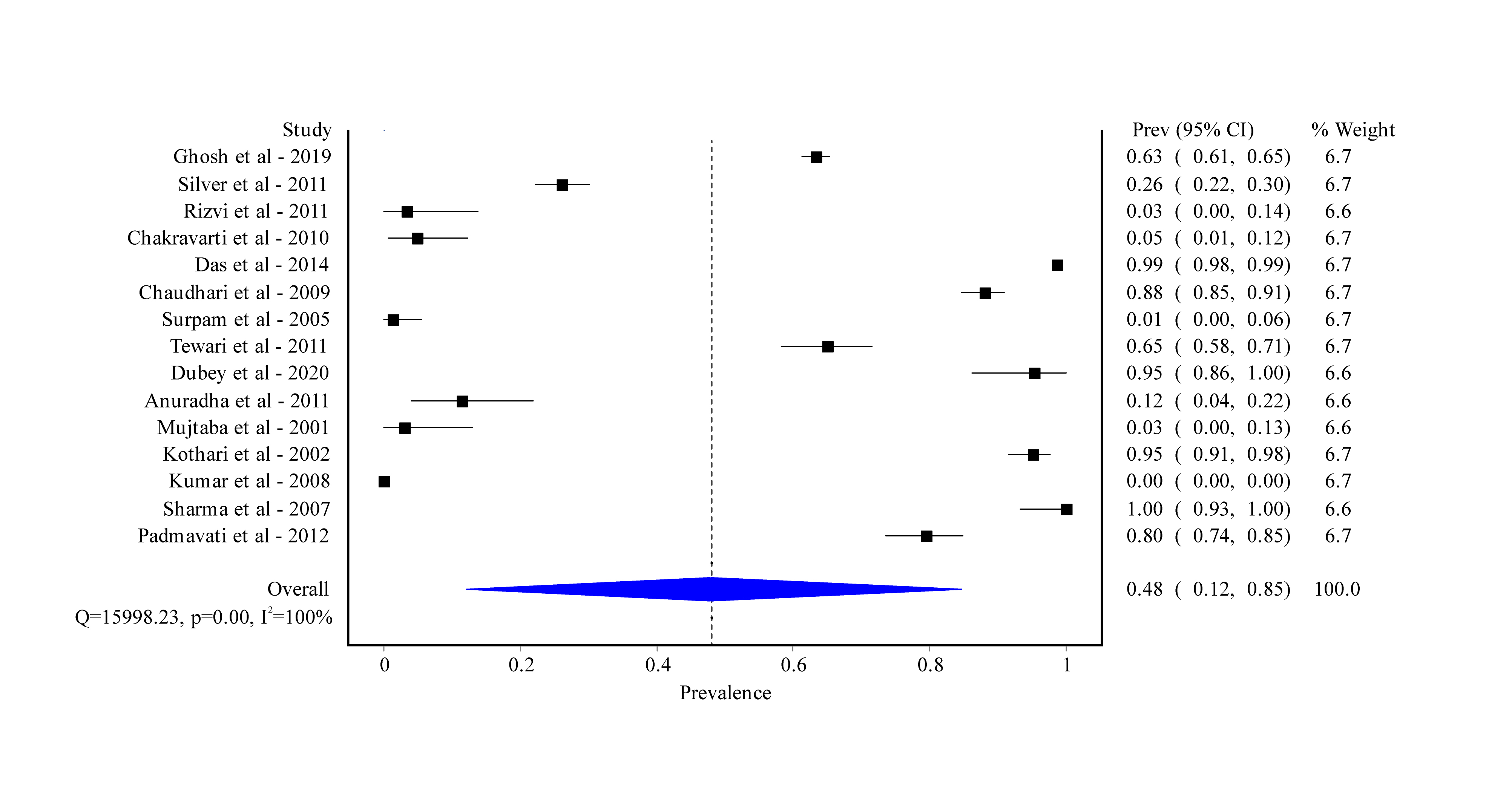

Supplement: S1 Fig — (JPG) [file pgph.0005728.s001.jpg]

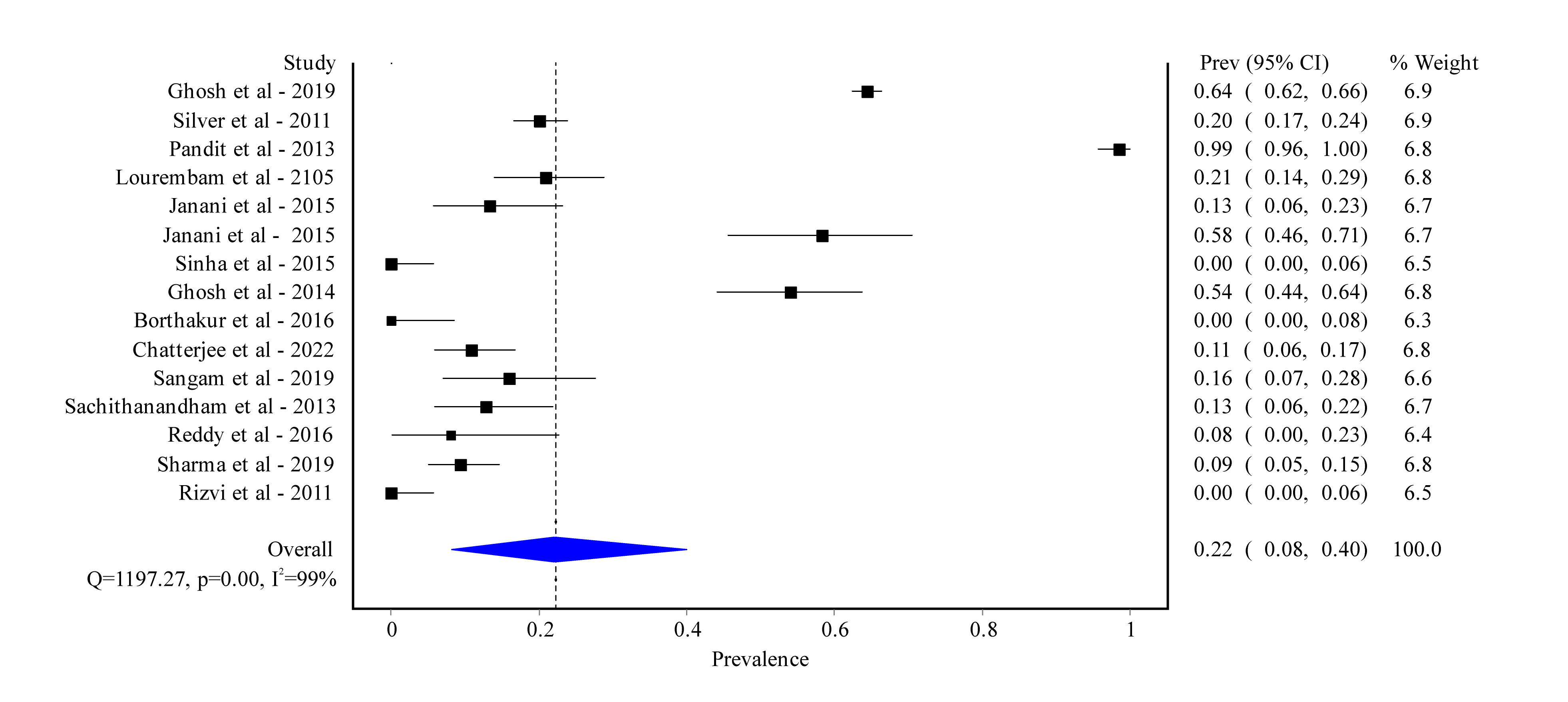

Supplement: S2 Fig — (JPG) [file pgph.0005728.s002.jpg]

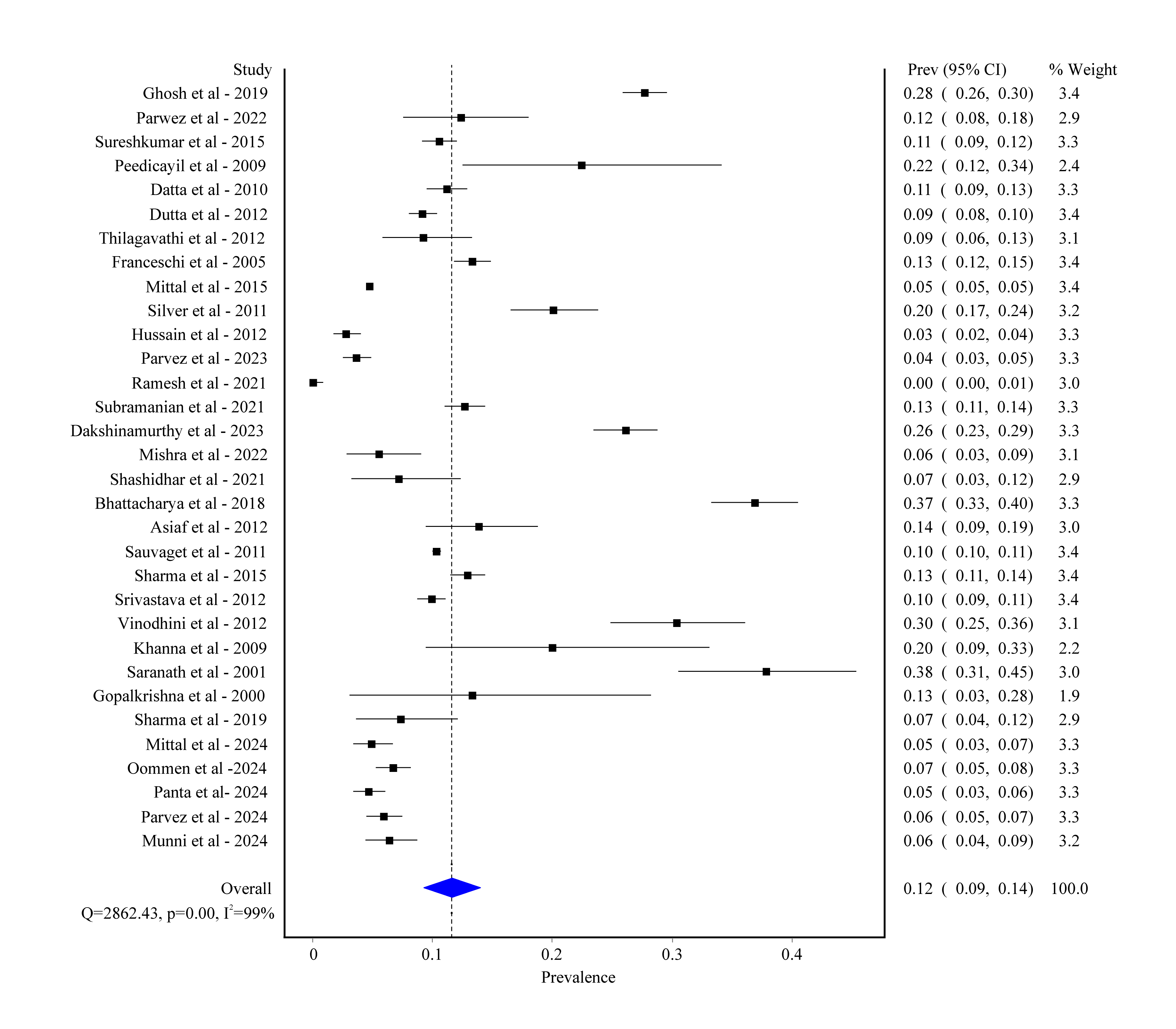

Supplement: S3 Fig — (JPG) [file pgph.0005728.s003.jpg]

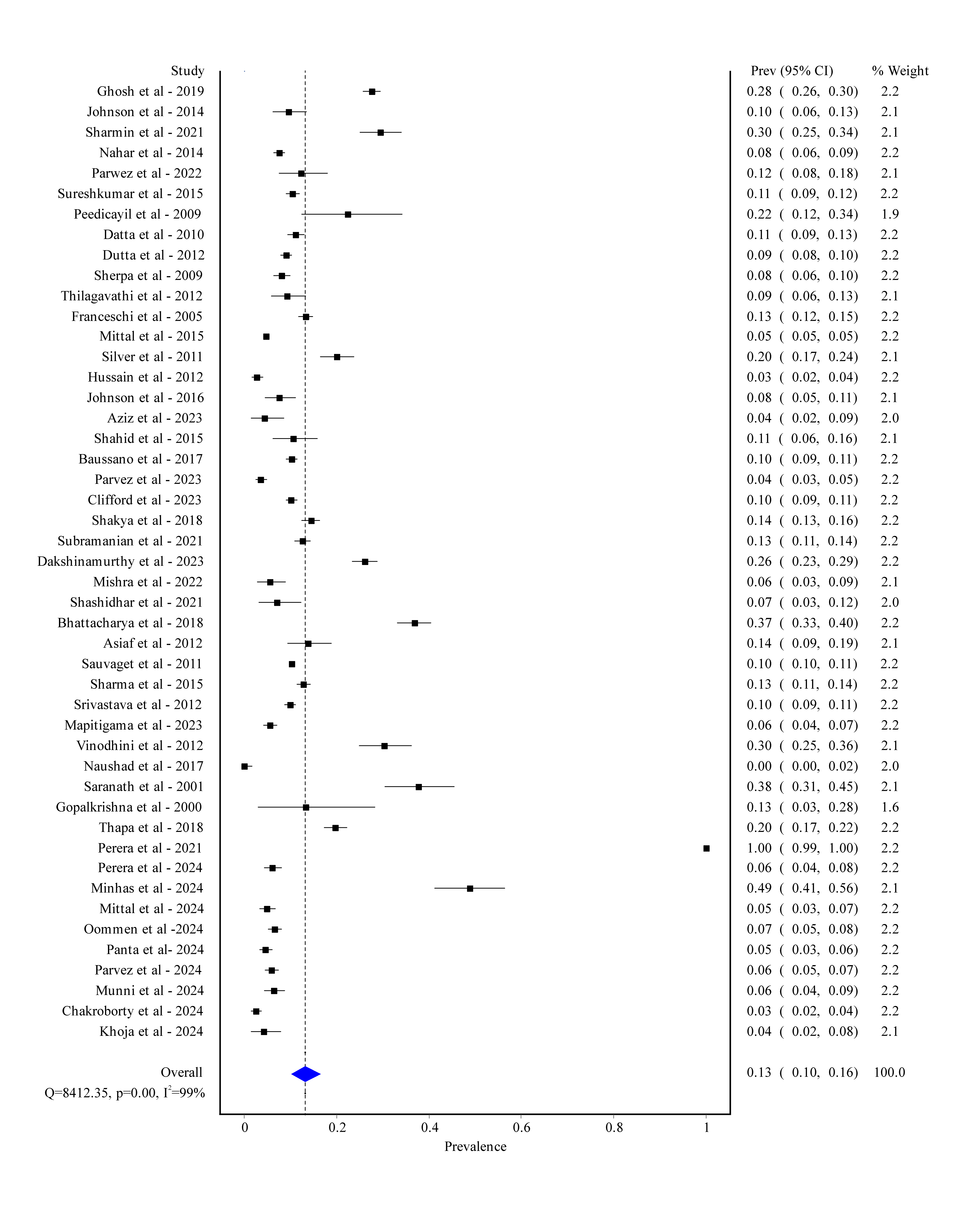

Supplement: S4 Fig — (JPG) [file pgph.0005728.s004.jpg]

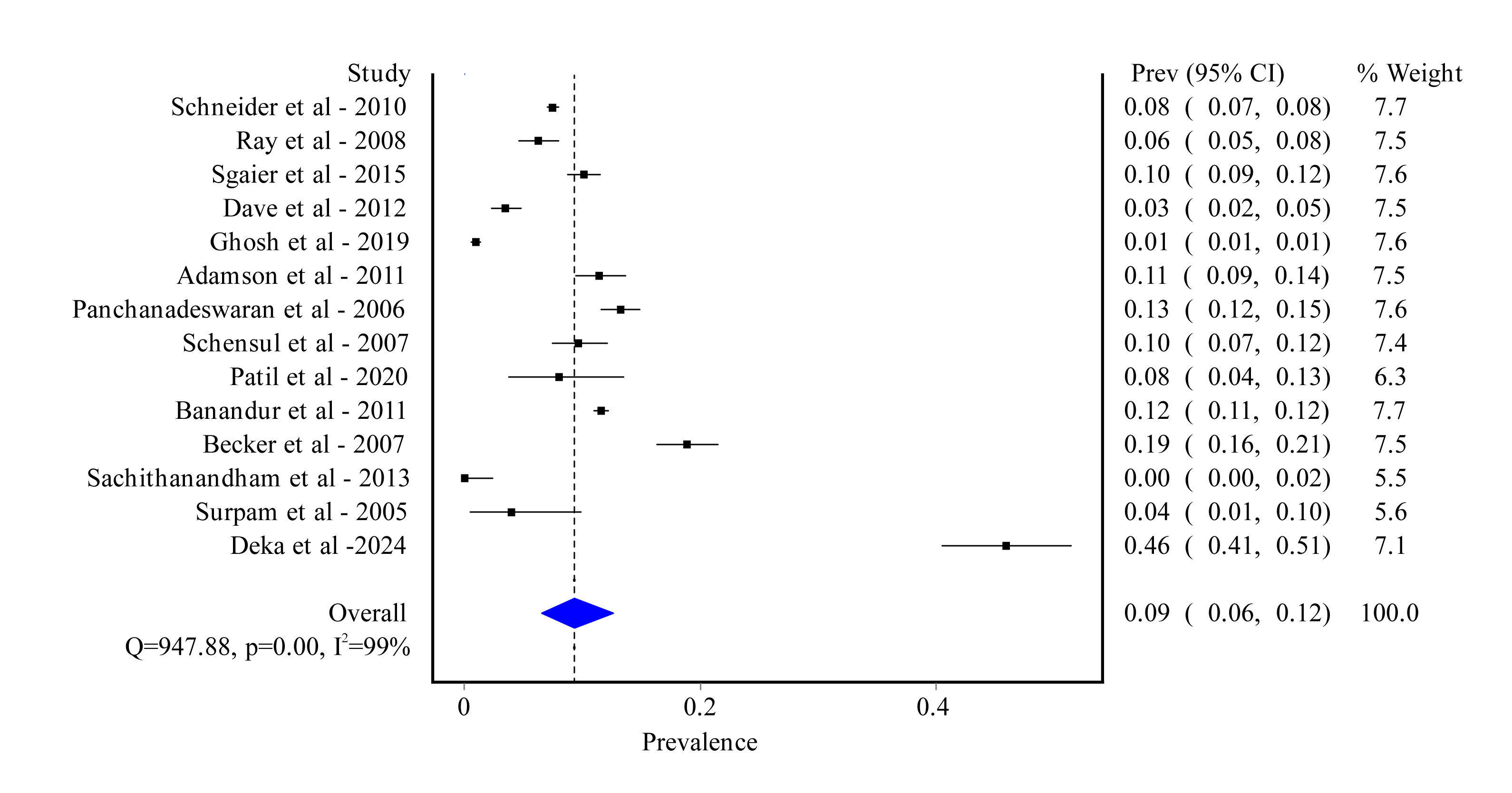

Supplement: S5 Fig — (JPG) [file pgph.0005728.s005.jpg]

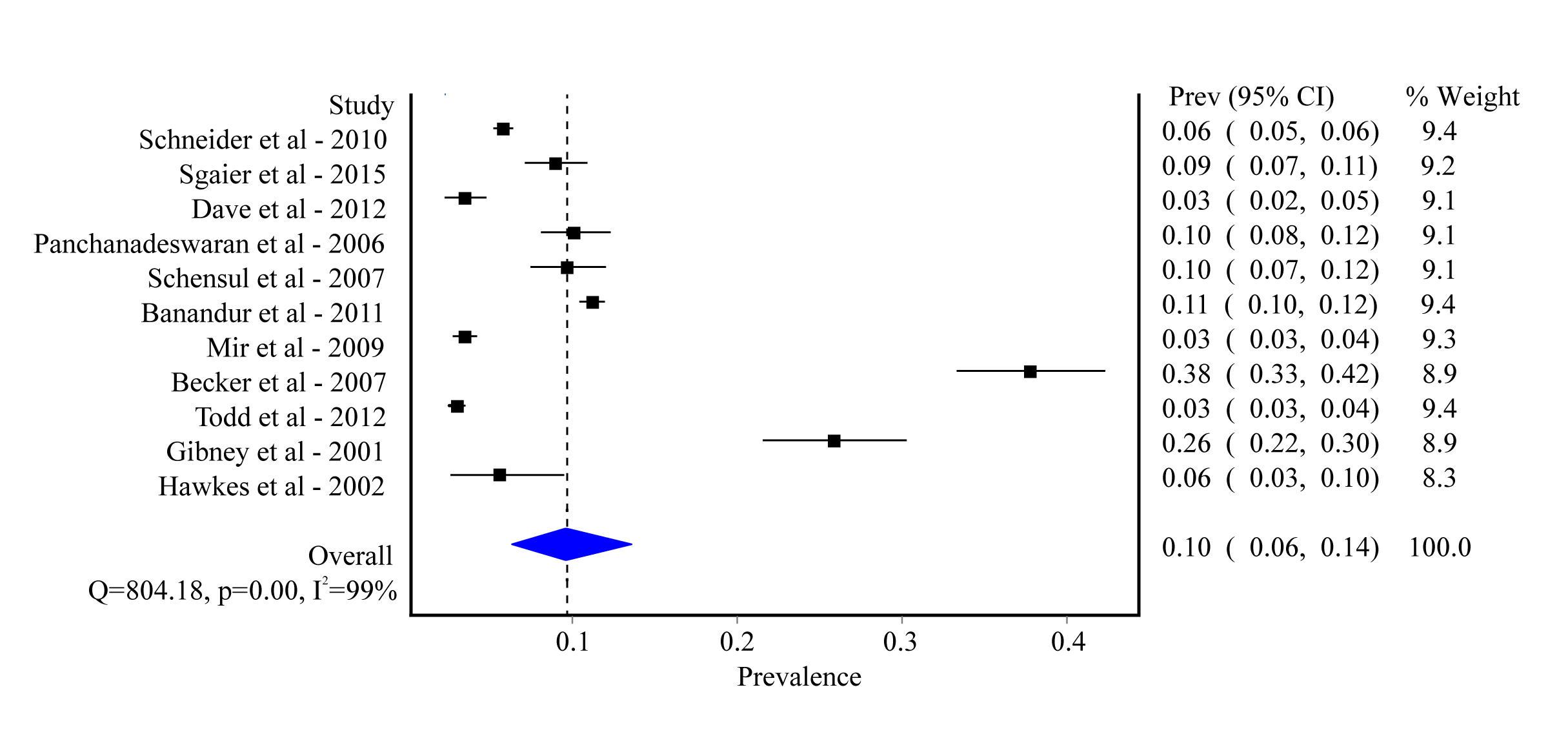

Supplement: S6 Fig — (JPG) [file pgph.0005728.s006.jpg]

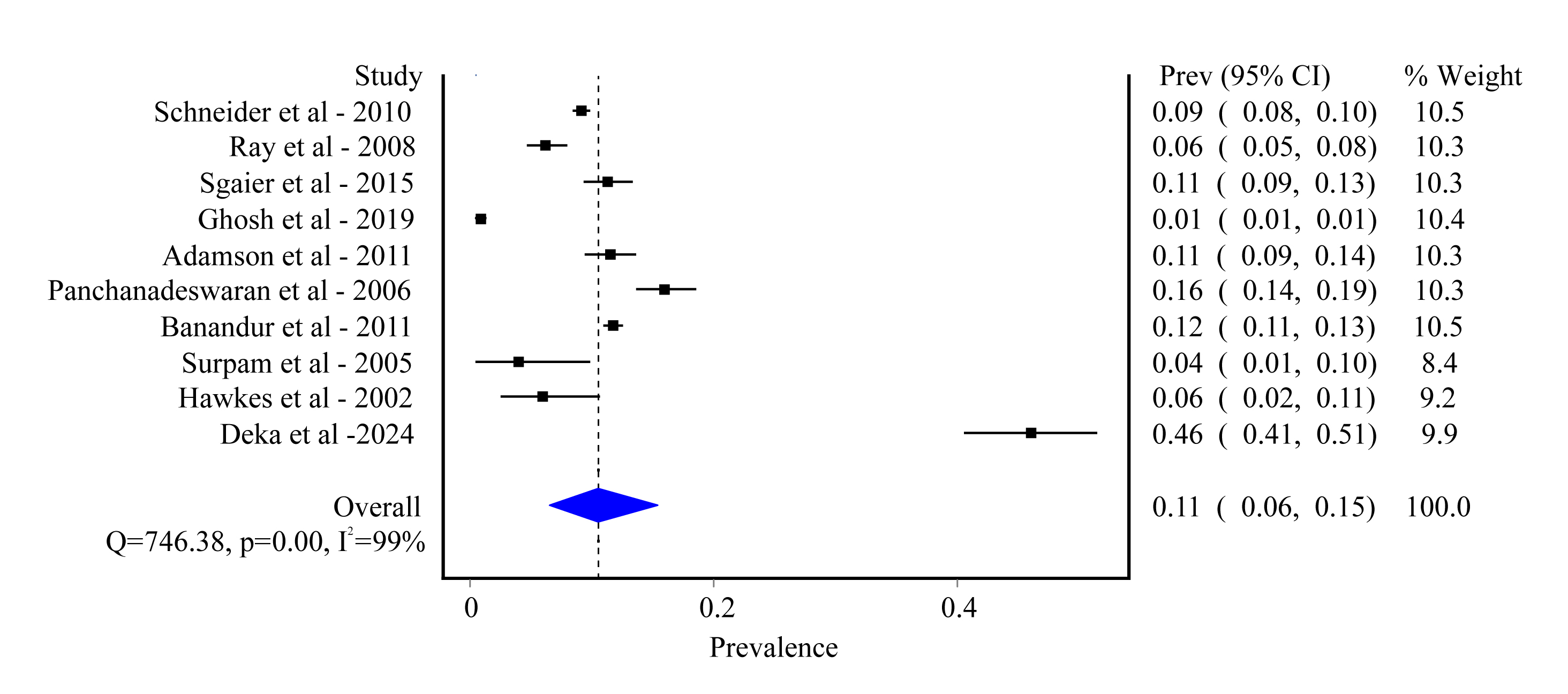

Supplement: S7 Fig — (JPG) [file pgph.0005728.s007.jpg]

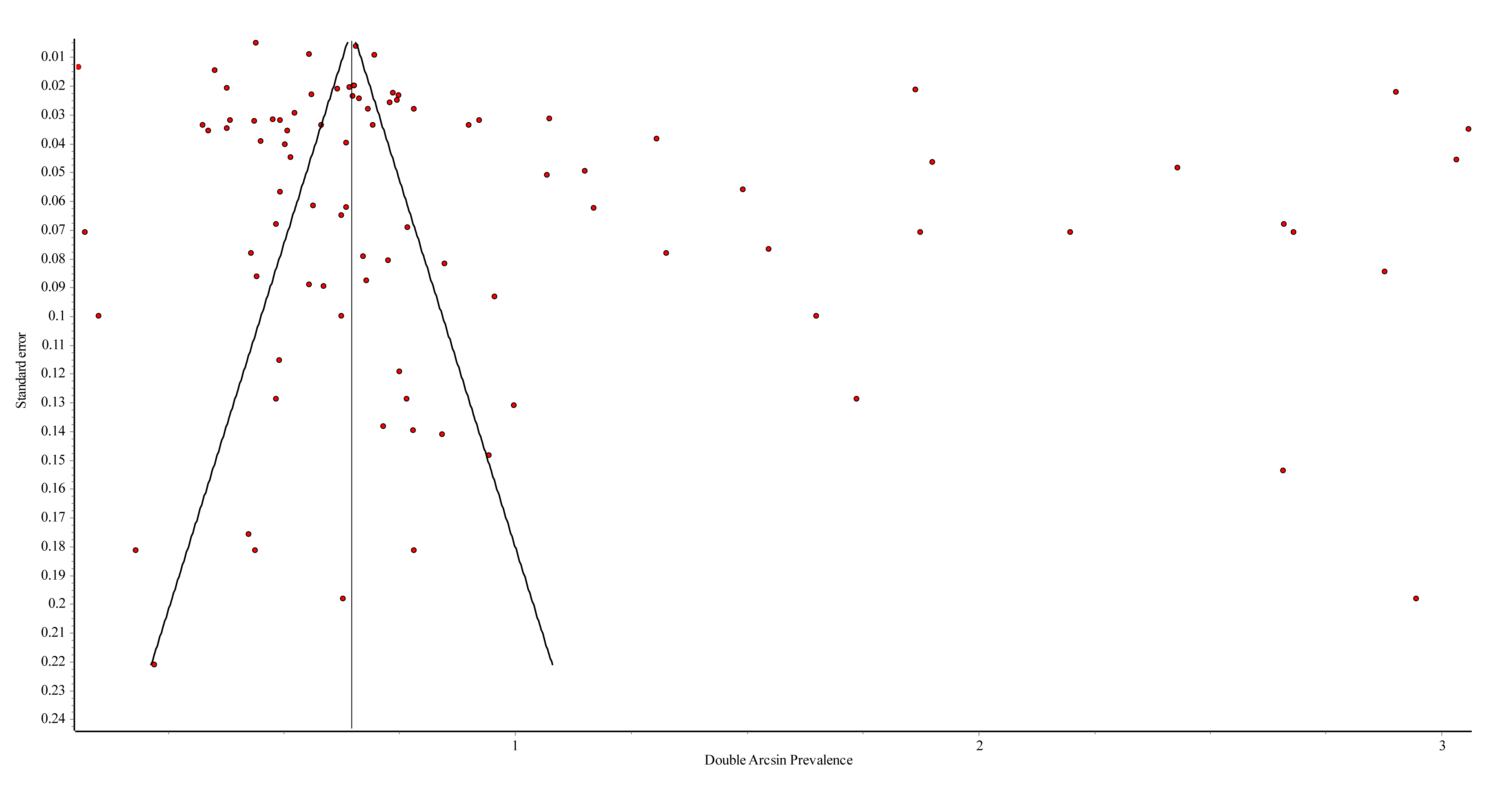

Supplement: S8 Fig — (JPG) [file pgph.0005728.s008.jpg]
