# Supplementary material for: Prevalence of CMV, EBV, HPV, and HSV among South Asian healthy population: A systematic review and meta-analysis
Source: PLOS Glob Public Health. 2026 Jan 7;6(1):e0005728. doi: 10.1371/journal.pgph.0005728 (PMC12779128; doi:10.1371/journal.pgph.0005728)
Supplement: S1 Table — (DOCX) [file pgph.0005728.s009.docx]

S1 Table: Searching detail, keywords, and alternative terms used in this study

| **PubMed** | **Scopus** | **Cochrane** | **Google Scholar** |
| --- | --- | --- | --- |
| ((CMV[Title/Abstract]) AND (Seroprevalence[Title/Abstract])) AND (Afghanistan[Title/Abstract])  ((CMV[Title/Abstract]) AND (Seroprevalence[Title/Abstract])) AND (Sri Lanka[Title/Abstract])  ((CMV[Title/Abstract]) AND (Seroprevalence[Title/Abstract])) AND (Nepal[Title/Abstract])  ((CMV[Title/Abstract]) AND (Seroprevalence[Title/Abstract])) AND (Bhutan[Title/Abstract])  ((CMV[Title/Abstract]) AND (Seroprevalence[Title/Abstract])) AND (Maldives[Title/Abstract])  ((CMV[Title/Abstract]) AND (Seroprevalence[Title/Abstract])) AND (Pakistan[Title/Abstract])  ((CMV[Title/Abstract]) AND (Seroprevalence[Title/Abstract])) AND (India[Title/Abstract])  ((CMV[Title/Abstract]) AND (Seroprevalence[Title/Abstract])) AND (Bangladesh[Title/Abstract])  ((CMV[Title/Abstract]) AND (seroprevalence[Title/Abstract])) AND (South Asia[Title/Abstract])  "Incidence"[Title/Abstract] AND "Epstein-Barr"[Title/Abstract] AND "Afghanistan"[Title/Abstract]  "Incidence"[Title/Abstract] AND "Epstein-Barr"[Title/Abstract] AND "Maldives"[Title/Abstract]  "Incidence"[Title/Abstract] AND "Epstein-Barr"[Title/Abstract] AND "Bhutan"[Title/Abstract]  "Incidence"[Title/Abstract] AND "Epstein-Barr"[Title/Abstract] AND "Nepal"[Title/Abstract]  "Incidence"[Title/Abstract] AND "Epstein-Barr"[Title/Abstract] AND "Nepal"[Title/Abstract]  "Incidence"[Title/Abstract] AND "Epstein-Barr"[Title/Abstract] AND "sri lanka"[Title/Abstract]  "Incidence"[Title/Abstract] AND "Epstein-Barr"[Title/Abstract] AND "Pakistan"[Title/Abstract]  "Incidence"[Title/Abstract] AND "Epstein-Barr"[Title/Abstract] AND "India"[Title/Abstract]  "Incidence"[Title/Abstract] AND "Epstein-Barr"[Title/Abstract] AND "Bangladesh"[Title/Abstract]  "Incidence"[Title/Abstract] AND "Epstein-Barr"[Title/Abstract] AND "south asia"[Title/Abstract]  "Seroprevalence"[Title/Abstract] AND "Epstein-Barr"[Title/Abstract] AND "Afghanistan"[Title/Abstract]  "Seroprevalence"[Title/Abstract] AND "Epstein-Barr"[Title/Abstract] AND "Maldives"[Title/Abstract]  "Seroprevalence"[Title/Abstract] AND "Epstein-Barr"[Title/Abstract] AND "Bhutan"[Title/Abstract]  "Seroprevalence"[Title/Abstract] AND "Epstein-Barr"[Title/Abstract] AND "Nepal"[Title/Abstract]  "Seroprevalence"[Title/Abstract] AND "Epstein-Barr"[Title/Abstract] AND "sri lanka"[Title/Abstract]  "Seroprevalence"[Title/Abstract] AND "Epstein-Barr"[Title/Abstract] AND "Pakistan"[Title/Abstract]  "Seroprevalence"[Title/Abstract] AND "Epstein-Barr"[Title/Abstract] AND "India"[Title/Abstract]  "Seroprevalence"[Title/Abstract] AND "Epstein-Barr"[Title/Abstract] AND "Bangladesh"[Title/Abstract]  "Seroprevalence"[Title/Abstract] AND "Epstein-Barr"[Title/Abstract] AND "south asia"[Title/Abstract]  "Seroprevalence"[Title/Abstract] AND "HPV"[Title/Abstract] AND "Afghanistan"[Title/Abstract]  "Seroprevalence"[Title/Abstract] AND "HPV"[Title/Abstract] AND "Maldives"[Title/Abstract]  "Seroprevalence"[Title/Abstract] AND "HPV"[Title/Abstract] AND "Bhutan"[Title/Abstract]  "Seroprevalence"[Title/Abstract] AND "HPV"[Title/Abstract] AND "Myanmar"[Title/Abstract]  "Seroprevalence"[Title/Abstract] AND "HPV"[Title/Abstract] AND "Pakistan"[Title/Abstract]  "Seroprevalence"[Title/Abstract] AND "HPV"[Title/Abstract] AND "Nepal"[Title/Abstract]  "Seroprevalence"[Title/Abstract] AND "HPV"[Title/Abstract] AND "sri lanka"[Title/Abstract]  "Seroprevalence"[Title/Abstract] AND "HPV"[Title/Abstract] AND "India"[Title/Abstract]  "Seroprevalence"[Title/Abstract] AND "HPV"[Title/Abstract] AND "Bangladesh"[Title/Abstract]  "Seroprevalence"[Title/Abstract] AND "HPV"[Title/Abstract] AND "south asia"[Title/Abstract]  "Prevalence"[Title/Abstract] AND "hsv 1"[Title/Abstract] AND "Afghanistan"[Title/Abstract]  "Prevalence"[Title/Abstract] AND "hsv 1"[Title/Abstract] AND "Maldives"[Title/Abstract]  "Prevalence"[Title/Abstract] AND "hsv 1"[Title/Abstract] AND "Bhutan"[Title/Abstract]  "Prevalence"[Title/Abstract] AND "hsv 1"[Title/Abstract] AND "Myanmar"[Title/Abstract]  "Prevalence"[Title/Abstract] AND "hsv 1"[Title/Abstract] AND "Pakistan"[Title/Abstract]  "Prevalence"[Title/Abstract] AND "hsv 1"[Title/Abstract] AND "Nepal"[Title/Abstract]  "Prevalence"[Title/Abstract] AND "hsv 1"[Title/Abstract] AND "Bangladesh"[Title/Abstract]  "Prevalence"[Title/Abstract] AND "hsv 1"[Title/Abstract] AND "India"[Title/Abstract]  "Prevalence"[Title/Abstract] AND "hsv 1"[Title/Abstract] AND "Sri Lanka"[Title/Abstract]  "Prevalence"[Title/Abstract] AND "hsv 1"[Title/Abstract] AND "South Asia"[Title/Abstract]  "Frequency"[Title/Abstract] AND "herpes simplex virus"[Title/Abstract] AND "Afghanistan"[Title/Abstract]  "Frequency"[Title/Abstract] AND "herpes simplex virus"[Title/Abstract] AND "Maldives"[Title/Abstract]  "Frequency"[Title/Abstract] AND "herpes simplex virus"[Title/Abstract] AND "Bhutan"[Title/Abstract]  "Frequency"[Title/Abstract] AND "herpes simplex virus"[Title/Abstract] AND "Myanmar"[Title/Abstract]  "Frequency"[Title/Abstract] AND "herpes simplex virus"[Title/Abstract] AND "Pakistan"[Title/Abstract]  "Frequency"[Title/Abstract] AND "herpes simplex virus"[Title/Abstract] AND "Nepal"[Title/Abstract]  "Frequency"[Title/Abstract] AND "herpes simplex virus"[Title/Abstract] AND "sri lanka"[Title/Abstract]  "Frequency"[Title/Abstract] AND "herpes simplex virus"[Title/Abstract] AND "India"[Title/Abstract]  "Frequency"[Title/Abstract] AND "herpes simplex virus"[Title/Abstract] AND "Bangladesh"[Title/Abstract]  "Frequency"[Title/Abstract] AND "herpes simplex virus"[Title/Abstract] AND "south asia"[Title/Abstract] | ( TITLE-ABS-KEY ( seroprevalence ) AND TITLE-ABS-KEY ( cytomegalovirus ) AND TITLE-ABS-KEY ( india ) )  ( TITLE-ABS-KEY ( seroprevalence ) AND TITLE-ABS-KEY ( cytomegalovirus ) AND TITLE-ABS-KEY ( bangladesh ) )  ( TITLE-ABS-KEY ( seroprevalence ) AND TITLE-ABS-KEY ( cytomegalovirus ) AND TITLE-ABS-KEY ( pakistan ) )  ( TITLE-ABS-KEY ( seroprevalence ) AND TITLE-ABS-KEY ( cytomegalovirus ) AND TITLE-ABS-KEY ( nepal ) )  ( TITLE-ABS-KEY ( seroprevalence ) AND TITLE-ABS-KEY ( cytomegalovirus ) AND TITLE-ABS-KEY ( sri AND lanka ) )  ( TITLE-ABS-KEY ( seroprevalence ) AND TITLE-ABS-KEY ( cytomegalovirus ) AND TITLE-ABS-KEY ( afghanistan ) )  ( TITLE-ABS-KEY ( seroprevalence ) AND TITLE-ABS-KEY ( cytomegalovirus ) AND TITLE-ABS-KEY ( maldives ) )  ( TITLE-ABS-KEY ( seroprevalence ) AND TITLE-ABS-KEY ( cytomegalovirus ) AND TITLE-ABS-KEY ( bhutan ) )  ( TITLE-ABS-KEY ( seroprevalence ) AND TITLE-ABS-KEY ( cytomegalovirus ) AND TITLE-ABS-KEY ( south AND asia ) )  ( TITLE-ABS-KEY ( prevalence ) AND TITLE-ABS-KEY ( epstein-barr AND virus ) AND TITLE-ABS-KEY ( south AND asia ) )  ( TITLE-ABS-KEY ( prevalence ) AND TITLE-ABS-KEY ( epstein-barr AND virus ) AND TITLE-ABS-KEY ( bangladesh ) )  ( TITLE-ABS-KEY ( prevalence ) AND TITLE-ABS-KEY ( epstein-barr AND virus ) AND TITLE-ABS-KEY ( india ) )  ( TITLE-ABS-KEY ( prevalence ) AND TITLE-ABS-KEY ( epstein-barr AND virus ) AND TITLE-ABS-KEY ( pakistan ) )  ( TITLE-ABS-KEY ( prevalence ) AND TITLE-ABS-KEY ( epstein-barr AND virus ) AND TITLE-ABS-KEY ( sri AND lanka ) )  ( TITLE-ABS-KEY ( prevalence ) AND TITLE-ABS-KEY ( epstein-barr AND virus ) AND TITLE-ABS-KEY ( nepal ) )  ( TITLE-ABS-KEY ( prevalence ) AND TITLE-ABS-KEY ( epstein-barr AND virus ) AND TITLE-ABS-KEY ( bhutan ) )  ( TITLE-ABS-KEY ( prevalence ) AND TITLE-ABS-KEY ( epstein-barr AND virus ) AND TITLE-ABS-KEY ( maldives ) )  ( TITLE-ABS-KEY ( prevalence ) AND TITLE-ABS-KEY ( epstein-barr AND virus ) AND TITLE-ABS-KEY ( afghanistan ) )  ( TITLE-ABS-KEY ( prevalence ) AND TITLE-ABS-KEY ( hpv ) AND TITLE-ABS-KEY ( south AND asia ) )  ( TITLE-ABS-KEY ( prevalence ) AND TITLE-ABS-KEY ( hpv ) AND TITLE-ABS-KEY ( bangladesh ) )  ( TITLE-ABS-KEY ( prevalence ) AND TITLE-ABS-KEY ( hpv ) AND TITLE-ABS-KEY ( india ) )  ( TITLE-ABS-KEY ( prevalence ) AND TITLE-ABS-KEY ( hpv ) AND TITLE-ABS-KEY ( sri AND lanka ) )  ( TITLE-ABS-KEY ( prevalence ) AND TITLE-ABS-KEY ( hpv ) AND TITLE-ABS-KEY ( nepal ) )  ( TITLE-ABS-KEY ( prevalence ) AND TITLE-ABS-KEY ( hpv ) AND TITLE-ABS-KEY ( pakistan ) )  ( TITLE-ABS-KEY ( prevalence ) AND TITLE-ABS-KEY ( hpv ) AND TITLE-ABS-KEY ( myanmar ) )  ( TITLE-ABS-KEY ( prevalence ) AND TITLE-ABS-KEY ( hpv ) AND TITLE-ABS-KEY ( bhutan ) )  ( TITLE-ABS-KEY ( prevalence ) AND TITLE-ABS-KEY ( hpv ) AND TITLE-ABS-KEY ( maldives ) )  ( TITLE-ABS-KEY ( prevalence ) AND TITLE-ABS-KEY ( hpv ) AND TITLE-ABS-KEY ( afghanistan ) )  ( TITLE-ABS-KEY ( hsv 1 ) AND TITLE-ABS-KEY ( seroprevalence ) AND TITLE-ABS-KEY ( south AND asia ) )  ( TITLE-ABS-KEY ( hsv 1 ) AND TITLE-ABS-KEY ( seroprevalence ) AND TITLE-ABS-KEY ( bangladesh ) )  ( TITLE-ABS-KEY ( hsv 1 ) AND TITLE-ABS-KEY ( seroprevalence ) AND TITLE-ABS-KEY ( india ) )  ( TITLE-ABS-KEY ( hsv 1 ) AND TITLE-ABS-KEY ( seroprevalence ) AND TITLE-ABS-KEY ( sri AND lanka ) )  ( TITLE-ABS-KEY ( hsv 1 ) AND TITLE-ABS-KEY ( seroprevalence ) AND TITLE-ABS-KEY ( nepal ) )  ( TITLE-ABS-KEY ( hsv 1 ) AND TITLE-ABS-KEY ( seroprevalence ) AND TITLE-ABS-KEY ( pakistan ) )  ( TITLE-ABS-KEY ( hsv 1 ) AND TITLE-ABS-KEY ( seroprevalence ) AND TITLE-ABS-KEY ( myanmar ) )  ( TITLE-ABS-KEY ( hsv 1 ) AND TITLE-ABS-KEY ( seroprevalence ) AND TITLE-ABS-K( TITLE-ABS-KEY ( incidence ) AND TITLE-ABS-KEY ( hsv ) AND TITLE-ABS-KEY ( india ) )  ( TITLE-ABS-KEY ( incidence ) AND TITLE-ABS-KEY ( hsv ) AND TITLE-ABS-KEY ( south AND asia ) )  ( TITLE-ABS-KEY ( incidence ) AND TITLE-ABS-KEY ( hsv ) AND TITLE-ABS-KEY ( bangladesh ) )  ( TITLE-ABS-KEY ( incidence ) AND TITLE-ABS-KEY ( hsv ) AND TITLE-ABS-KEY ( sri AND lanka ) )  ( TITLE-ABS-KEY ( incidence ) AND TITLE-ABS-KEY ( hsv ) AND TITLE-ABS-KEY ( nepal ) )  ( TITLE-ABS-KEY ( incidence ) AND TITLE-ABS-KEY ( hsv ) AND TITLE-ABS-KEY ( pakistan ) )  ( TITLE-ABS-KEY ( incidence ) AND TITLE-ABS-KEY ( hsv ) AND TITLE-ABS-KEY ( myanmar ) )  ( TITLE-ABS-KEY ( incidence ) AND TITLE-ABS-KEY ( hsv ) AND TITLE-ABS-KEY ( bhutan ) )  ( TITLE-ABS-KEY ( incidence ) AND TITLE-ABS-KEY ( hsv ) AND TITLE-ABS-KEY ( maldives ) )  ( TITLE-ABS-KEY ( incidence ) AND TITLE-ABS-KEY ( hsv ) AND TITLE-ABS-KEY ( afghanistan ) )EY ( afghanistan ) ) | ("Epstein Barr virus"):ti,ab,kw AND ("pregnant"):ti,ab,kw AND ("South Asia"):ti,ab,kw (Word variations have been searched)  ("Epstein Barr virus"):ti,ab,kw AND ("pregnant women"):ti,ab,kw AND ("South Asia"):ti,ab,kw (Word variations have been searched)  ("Epstein Barr virus"):ti,ab,kw AND ("pregnant women"):ti,ab,kw AND ("Bangladesh"):ti,ab,kw (Word variations have been searched)  ("Epstein Barr virus"):ti,ab,kw AND ("pregnant women"):ti,ab,kw AND ("India"):ti,ab,kw (Word variations have been searched)  ("Epstein Barr virus"):ti,ab,kw AND ("pregnant women"):ti,ab,kw AND ("Pakistan"):ti,ab,kw (Word variations have been searched)  ("Epstein Barr virus"):ti,ab,kw AND ("pregnant women"):ti,ab,kw AND ("Sri Lanka"):ti,ab,kw (Word variations have been searched)  ("Epstein Barr virus"):ti,ab,kw AND ("pregnant women"):ti,ab,kw AND ("Nepal"):ti,ab,kw (Word variations have been searched)  ("Epstein Barr virus"):ti,ab,kw AND ("pregnant women"):ti,ab,kw AND ("Bhutan"):ti,ab,kw (Word variations have been searched)  ("Epstein Barr virus"):ti,ab,kw AND ("pregnant women"):ti,ab,kw AND ("Maldives"):ti,ab,kw (Word variations have been searched)  ("Epstein Barr virus"):ti,ab,kw AND ("pregnant women"):ti,ab,kw AND ("Afghanistan"):ti,ab,kw (Word variations have been searched)  ("EBV"):ti,ab,kw AND ("pregnant women"):ti,ab,kw AND ("South Asia"):ti,ab,kw (Word variations have been searched)  ("EBV"):ti,ab,kw AND ("pregnant women"):ti,ab,kw AND ("Bangladesh"):ti,ab,kw (Word variations have been searched)  ("EBV"):ti,ab,kw AND ("pregnant women"):ti,ab,kw AND ("India"):ti,ab,kw (Word variations have been searched)  ("EBV"):ti,ab,kw AND ("pregnant women"):ti,ab,kw AND ("Pakistan"):ti,ab,kw (Word variations have been searched)  ("EBV"):ti,ab,kw AND ("pregnant women"):ti,ab,kw AND ("Sri Lanka"):ti,ab,kw (Word variations have been searched)  ("EBV"):ti,ab,kw AND ("pregnant women"):ti,ab,kw AND ("nepal"):ti,ab,kw (Word variations have been searched)  ("EBV"):ti,ab,kw AND ("pregnant women"):ti,ab,kw AND ("Bhutan"):ti,ab,kw (Word variations have been searched)  ("EBV"):ti,ab,kw AND ("pregnant women"):ti,ab,kw AND ("Maldives"):ti,ab,kw (Word variations have been searched)  ("EBV"):ti,ab,kw AND ("pregnant women"):ti,ab,kw AND ("Afghanistan"):ti,ab,kw (Word variations have been searched)  ("Epstein Barr virus"):ti,ab,kw AND ("Seroprevalence"):ti,ab,kw AND ("South Asia"):ti,ab,kw (Word variations have been searched)  ("Epstein Barr virus"):ti,ab,kw AND ("Seroprevalence"):ti,ab,kw AND ("Bangladesh"):ti,ab,kw (Word variations have been searched)  ("Epstein Barr virus"):ti,ab,kw AND ("Seroprevalence"):ti,ab,kw AND ("India"):ti,ab,kw (Word variations have been searched)  ("Epstein Barr virus"):ti,ab,kw AND ("Seroprevalence"):ti,ab,kw AND ("Pakistan"):ti,ab,kw (Word variations have been searched)  ("Epstein Barr virus"):ti,ab,kw AND ("Seroprevalence"):ti,ab,kw AND ("Sri Lanka"):ti,ab,kw (Word variations have been searched)  ("Epstein Barr virus"):ti,ab,kw AND ("Seroprevalence"):ti,ab,kw AND ("Nepal"):ti,ab,kw (Word variations have been searched)  ("Epstein Barr virus"):ti,ab,kw AND ("Seroprevalence"):ti,ab,kw AND ("Bhutan"):ti,ab,kw (Word variations have been searched)  ("Epstein Barr virus"):ti,ab,kw AND ("Seroprevalence"):ti,ab,kw AND ("Maldives"):ti,ab,kw (Word variations have been searched)  ("Epstein Barr virus"):ti,ab,kw AND ("Seroprevalence"):ti,ab,kw AND ("Afghanistan"):ti,ab,kw (Word variations have been searched)  (Seroprevalence):ti,ab,kw AND (HSV):ti,ab,kw AND (South Asia):ti,ab,kw (Word variations have been searched)  (Seroprevalence):ti,ab,kw AND (HSV):ti,ab,kw AND (Bangladesh):ti,ab,kw (Word variations have been searched)  (Seroprevalence):ti,ab,kw AND (HSV):ti,ab,kw AND (India):ti,ab,kw (Word variations have been searched)  (Seroprevalence):ti,ab,kw AND (HSV):ti,ab,kw AND (Sri Lanka):ti,ab,kw (Word variations have been searched)  (Seroprevalence):ti,ab,kw AND (HSV):ti,ab,kw AND (Nepal):ti,ab,kw (Word variations have been searched)  (Seroprevalence):ti,ab,kw AND (HSV):ti,ab,kw AND (Pakistan):ti,ab,kw (Word variations have been searched)  (Seroprevalence):ti,ab,kw AND (HSV):ti,ab,kw AND (Bhutan):ti,ab,kw (Word variations have been searched)  (Seroprevalence):ti,ab,kw AND (HSV):ti,ab,kw AND (Maldives):ti,ab,kw (Word variations have been searched)  (Seroprevalence):ti,ab,kw AND (HSV):ti,ab,kw AND (Afghanistan):ti,ab,kw (Word variations have been searched)  (Seroprevalence):ti,ab,kw AND (HPV):ti,ab,kw AND (South Asia):ti,ab,kw (Word variations have been searched)  (Seroprevalence):ti,ab,kw AND (HPV):ti,ab,kw AND (Bangladesh):ti,ab,kw (Word variations have been searched)  (Seroprevalence):ti,ab,kw AND (HPV):ti,ab,kw AND (India):ti,ab,kw (Word variations have been searched)  (Seroprevalence):ti,ab,kw AND (HPV):ti,ab,kw AND (Sri Lanka):ti,ab,kw (Word variations have been searched)  (Seroprevalence):ti,ab,kw AND (HPV):ti,ab,kw AND (Nepal):ti,ab,kw (Word variations have been searched)  (Seroprevalence):ti,ab,kw AND (HPV):ti,ab,kw AND (Pakistan):ti,ab,kw (Word variations have been searched)  (Seroprevalence):ti,ab,kw AND (HPV):ti,ab,kw AND (Bhutan):ti,ab,kw (Word variations have been searched)  (Seroprevalence):ti,ab,kw AND (HPV):ti,ab,kw AND (Maldives):ti,ab,kw (Word variations have been searched)  (Seroprevalence):ti,ab,kw AND (HPV):ti,ab,kw AND (Afghanistan):ti,ab,kw (Word variations have been searched) | ((“Prevalence”) OR (“Seroprevalence”)) AND ((Cytomegalovirus) OR (CMV)) AND (healthy adults) AND (South Asia)  ((“Prevalence”) OR (“Seroprevalence”)) AND ((Epstein-Barr virus) OR (EBV)) AND (healthy adults) AND (South Asia)  ((“Prevalence”) OR (“Seroprevalence”)) AND ((Human papilloma virus) OR (HPV)) AND (healthy adults) AND (South Asia)  ((“Prevalence”) OR (“Seroprevalence”)) AND ((Herpes Simplex virus) OR (HSV)) AND (healthy adults) AND (South Asia)  ((“Prevalence”) OR (“Seroprevalence”)) AND ((Cytomegalovirus) OR (CMV)) AND (healthy adults) AND (Bangladesh)  ((“Prevalence”) OR (“Seroprevalence”)) AND ((Epstein-Barr virus) OR (EBV)) AND (healthy adults) AND (India)  ((“Prevalence”) OR (“Seroprevalence”)) AND ((Human papilloma virus) OR (HPV)) AND (healthy adults) AND (Pakistan)  ((“Prevalence”) OR (“Seroprevalence”)) AND ((Herpes Simplex virus) OR (HSV)) AND (healthy adults) AND (Sri lanka)  ((“Prevalence”) OR (“Seroprevalence”)) AND ((Cytomegalovirus) OR (CMV)) AND (healthy adults) AND (Nepal)  ((“Prevalence”) OR (“Seroprevalence”)) AND ((Epstein-Barr virus) OR (EBV)) AND (healthy adults) AND (Bhutan)  ((“Prevalence”) OR (“Seroprevalence”)) AND ((Human papilloma virus) OR (HPV)) AND (healthy adults) AND (Afghanistan)  ((“Prevalence”) OR (“Seroprevalence”)) AND ((Herpes Simplex virus) OR (HSV)) AND (healthy adults) AND (Maldives) |
