# Supplementary material for: Prevalence of CMV, EBV, HPV, and HSV among South Asian healthy population: A systematic review and meta-analysis
Source: PLOS Glob Public Health. 2026 Jan 7;6(1):e0005728. doi: 10.1371/journal.pgph.0005728 (PMC12779128; doi:10.1371/journal.pgph.0005728)
Supplement: S2 Table — (DOCX) [file pgph.0005728.s010.docx]

S2 Table: Characteristics of the selected studies

| **Reference** | **Study Location** | **Study Setting** | **Study Period** | **Sample type** |
| --- | --- | --- | --- | --- |
| Schneider et al – 2010 [50] | Andhra Pradesh | Community-based | September 2004 and September 2005 | Blood |
| Ray et al – 2008 [51] | Delhi | Hospital-based | March 2002 - April 2004 | Blood |
| Sgaier et al – 2015 [52] | Hyderabad, Bangalore, Chandigarh | Community-based | 2001-2006 | Blood |
| Dave et al – 2012 [53] | Surat city | Community-based | 2005 | Blood |
| Ghosh et al – 2019 [54] | Southern coastal Karnataka | Community-based | Not given | Cervical specimen |
| Adamson et al – 2011 [55] | Mysore | Hospital-based | November 2005 - March 2006 | Blood |
| Panchanadeswaran et al – 2006 [56] | Chennai, Tamil Nadu | Community-based | March and June 2001 | Blood |
| Schensul et al – 2007 [57] | Mumbai | Community-based | June - September 2003 | Blood |
| Patil et al – 2020 [58] | Uttar Pradesh | Community-based | November 2017 - April 2018 | Blood |
| Banandur et al – 2011 [59] | Karnataka | Community-based | Between 2006 and 2008 | Blood |
| Mir et al – 2009 [60] | Pakistan | Community-based | 2007 | Blood |
| Johnson et al – 2014 [61] | Western Nepal | Community-based | 2013 | Cervical specimen |
| Sharmin et al – 2021 [62] | Bangladesh | Hospital-based | February 2015 and June 2018 | Cervical specimen |
| Nahar et al – 2014 [63] | Dhaka | Community-based | July and December 2011 | Cervical specimen |
| Parwez et al – 2022 [64] | Bihar | Community-based | Not given | Urine |
| Sureshkumar et al – 2015 [65] | India | Community-based | January 2007 and December 2009 | Urine |
| Peedicayil et al – 2009 [66] | India | Hospital-based | Not given | Cervical specimen |
| Datta et al – 2010 [67] | New Delhi | Community-based | Not given | Cervical specimen |
| Dutta et al – 2012 [68] | Eastern India | Community-based | September 2007 - March 2010 | Cervical specimen |
| Sherpa et al – 2009 [69] | Nepal | Community-based | October 2006 and March 2007 | Cervical specimen |
| Thilagavathi et al – 2012 [70] | Tamilnadu | Community-based | August 2009 and July 2010 | Urine |
| Franceschi et al – 2005 [71] | India | Community-based | February and October 2003 | Cervical specimen |
| Mittal et al – 2015 [72] | Kolkata | Community-based | July 2010 and March 2015 | Cervical specimen |
| Silver et al – 2011 [73] | Hydrabad | Community-based | Not given | Cervical specimen |
| Hussain et al – 2012 [74] | India | Community-based | Not given | Urine |
| Johnson et al – 2016 [75] | Nepal | Community-based | July, 2013 | Cervical specimen |
| Aziz et al – 2023 [76] | Pakistan | Hospital-based | March 2017 and August 2019 | Cervical specimen |
| Shahid et al – 2015 [77] | Pakistan | Hospital-based | April 2012 - December 2012 | Cervical specimen |
| Baussano et al – 2017 [78] | Bhutan | Community-based | April-May 2016 | Cervical specimen |
| Becker et al – 2007 [79] | India | Community-based | April-September 2003 | Blood |
| Parvez et al – 2023 [80] | India | Community-based | Not given | Cervical specimen |
| Clifford et al – 2023 [81] | Bhutan | Community-based | April - May 2016 | Cervical specimen |
| Shakya et al – 2018 [82] | Nepal | Community-based | February 2012 - May 2013 | Cervical specimen |
| Todd et al – 2012 [83] | Kabul | Community-based | February 2010 and January 2011 | Blood |
| Ramesh et al – 2021 [84] | India | Hospital-based | March 2019 - December 2019 | Oral specimen |
| Subramanian et al – 2021 [85] | India | Community-based | February 2009 - February 2014, | Cervical specimen |
| Dakshinamurthy et al – 2023 [86] | India | Hospital-based | Not given | Cervical specimen |
| Mishra et al – 2022 [87] | India | Hospital-based | May 2019 - April 2021 | Cervical specimen |
| Shashidhar et al – 2021 [88] | India | Hospital-based | Not given | Cervical specimen |
| Bhattacharya et al – 2018 [89] | India | Hospital-based | Not given | Cervical specimen |
| Asiaf et al – 2012 [90] | Kashmir | Hospital-based | May 2010 and April 2011 | Cervical specimen |
| Sauvaget et al – 2011 [91] | India | Community-based | 2000 January | Cervical specimen |
| Sharma et al – 2015 [92] | India | Community-based | March-December 2012 | Urine |
| Srivastava et al – 2012 [93] | India | Community-based | October 2005 - December 2010 | Cervical specimen |
| Mapitigama et al – 2023 [94] | Sri Lanka | Hospital-based | February 2019 - July 2019 | Cervical specimen |
| Vinodhini et al – 2012 [95] | Tamil Nadu | Hospital-based | May 2009 and May 2011 | Cervical specimen |
| Khanna et al – 2009 [96] | India | Hospital-based | 2 years | Normal mucosa |
| Naushad et al – 2017 [97] | Pakistan | Hospital-based | 2012-2014 | Blood |
| Gunasekera et al – 2015 [98] | Sri Lanka | Hospital-based | Not given | Blood |
| Saranath et al – 2001 [99] | India | Hospital-based | Not given | Cervical specimen |
| Gopalkrishna et al – 2000 [100] | New Delhi | Hospital-based | Not given | Cervical specimen |
| Pandit et al – 2013 [101] | Mumbai, Mangalore | Hospital-based | Not given | Blood |
| Lourembam et al – 2015 [102] | Northeastern India | Hospital-based | April 2011 - November 2013 | Blood |
| Janani et al – 2015 [103] | Chennai | Hospital-based | Not given | Blood |
| Janani et al – 2015 [104] | Chennai | Hospital-based | August 2010 - July 2013 | Blood |
| Sinha et al – 2015 [105] | South India | Hospital-based | 10 months | Blood |
| Ghosh et al – 2014 [106] | Northeast India | Hospital-based | Not given | Oral/Blood/Tissue |
| Noorali et al – 2004 [107] | Karachi | Hospital-based | January 01, 1992 - December 31, 2002 | Blood |
| Borthakur et al – 2016 [108] | Northeast India | Hospital-based | Not given | Tissue |
| Chatterjee et al – 2022 [109] | North-Eastern India | Hospital-based | May 2014 - April 2019 | Blood |
| Sangam et al – 2019 [110] | India | Hospital-based | Not given | Blood |
| Sachithanandham et al – 2013 [111] | South India | Hospital-based | Not given | Blood |
| Reddy et al – 2016 [112] | South India | Hospital-based | Not given | Tissue |
| Sharma et al – 2019 [113] | South India | Hospital-based | Not given | Oral specimen |
| Rizvi et al – 2011 [114] | North India | Hospital-based | June 2009 - March 2010 | Blood |
| Husseini et al – 2019 [115] | Main provinces | Community-based | Not given | Blood |
| Chakravarti et al – 2010 [116] | Delhi | Hospital-based | October 2007 - April 2009. | Blood |
| Das et al – 2014 [117] | Chandigarh | Community-based | January 2011 - July 2012 | Blood |
| Chaudhari et al – 2009 [1181] | India | Hospital-based | Not given | Blood |
| Surpam et al – 2005 [119] | India | Not given | Not given | Blood |
| Tewari et al – 2011 [120] | North India | Hospital-based | 2 years | Blood |
| Dubey et al – 2020 [121] | Mumbai | Hospital-based | Not given | Blood |
| Anuradha et al – 2011 [122] | Andhra Pradesh | Hospital-based | 6 months | Blood |
| Mujtaba et al – 2001 [123] | Chandigarh | Hospital-based | 1997-1999 | Blood |
| Kothari et al – 2002 [124] | Delhi | Hospital-based | 4 days | Blood |
| Kumar et al – 2008 [125] | India | Hospital-based | Not given | Blood |
| Sharma et al – 2007 [126] | Guwahati | Hospital-based | 1 year | Blood |
| Padmavati et al – 2012 [127] | Delhi | Hospital-based | 2000-2004 | Blood |
| Thapa et al – 2018 [128] | Jumla | Community-based | May 2016 - January 2017 | Cervical specimen |
| Perera et al – 2021 [129] | Kalutara | Community-based | 1 July 2018 - 30 November 2018 | Cervical specimen |
| Gibney et al – 2001 [130] | Tejgaon | Community-based | October 1998 - April 1999 | Blood |
| Ibrahim et al – 2016 [131] | Karachi | Community-based | July 2010 - June 2012 | Blood |
| Hawkes et al – 2002 [132] | Matlab | Community-based | August 1995 | Blood |
| Munir et al – 2023 [133] | Khyber Pakhtunkhwa | Hospital-based | 21 July 2022, 19 August 2023 | Blood |
| Perera et al - 2024 [134] | Kalutara | Community-based | 1 September 2018 - 31 January 2019 | Cervical specimen |
| Minhas et al - 2024 [135] | Lahore | Hospital-based | September 2018 - August 2020 | Cervical specimen |
| Mittal et al - 2024 [136] | Dehradun | Hospital-based | January 2021 - April 2022 | Cervical specimen |
| Oommen et al -2024 [137] | India | Community-based | December 2021 - July 2022 | Cervical specimen |
| Panta et al- 2024 [138] | Uttarakand | Community-based | Not given | Cervical specimen |
| Parvez et al - 2024 [139] | Andaman | Community-based | 2018-2022 | Cervical specimen |
| Munni et al - 2024 [140] | Maharashtra | Hospital-based | December 2020 - October 2022 | Cervical specimen |
| Chakroborty et al - 2024 [141] | Coastal districts | Community-based | January 2023 - December 2023 | Cervical specimen |
| Khoja et al - 2024 [142] | Semi-urban settings | Hospital-based | June 2021 - January 2022 | Cervical specimen |
| Deka et al -2024 [143] | Uttarakand | Hospital-based | May 2015 - December 2019 | Blood |
