# Supplementary material for: Prevalence of CMV, EBV, HPV, and HSV among South Asian healthy population: A systematic review and meta-analysis
Source: PLOS Glob Public Health. 2026 Jan 7;6(1):e0005728. doi: 10.1371/journal.pgph.0005728 (PMC12779128; doi:10.1371/journal.pgph.0005728)
Supplement: S3 Table — (DOCX) [file pgph.0005728.s011.docx]

S3 Table: Quality assessment of selected studies (Q1, Q2, Q3......Q9 denotes nine parameters described by Joanna Briggs Institute Prevalence Critical Appraisal Tool)

| **Reference** | **Q1** | **Q2** | **Q3** | **Q4** | **Q5** | **Q6** | **Q7** | **Q8** | **Q9** | **Total Score** | **Risk** |
| --- | --- | --- | --- | --- | --- | --- | --- | --- | --- | --- | --- |
| Schneider et al – 2010 [50] | 1 | 1 | 1 | 1 | 1 | 1 | 1 | 1 | 1 | 9 | Low |
| Ray et al – 2008 [51] | 1 | 0 | 1 | 1 | 1 | 1 | 1 | 1 | 1 | 8 | Low |
| Sgaier et al – 2015 [52] | 1 | 1 | 1 | 1 | 1 | 1 | 1 | 1 | 1 | 9 | Low |
| Dave et al – 2012 [53] | 0 | 1 | 1 | 1 | 1 | 1 | 1 | 1 | 1 | 8 | Low |
| Ghosh et al – 2019 [54] | 1 | 1 | 1 | 1 | 1 | 1 | 1 | 1 | 1 | 9 | Low |
| Adamson et al – 2011 [55] | 1 | 1 | 1 | 1 | 1 | 1 | 1 | 1 | 1 | 9 | Low |
| Panchanadeswaran et al – 2006 [56] | 1 | 1 | 1 | 1 | 1 | 1 | 1 | 1 | 1 | 9 | Low |
| Schensul et al – 2007 [57] | 1 | 1 | 1 | 1 | 1 | 1 | 1 | 1 | 1 | 9 | Low |
| Patil et al – 2020 [58] | 1 | 1 | 1 | 1 | 1 | 1 | 1 | 1 | 1 | 9 | Low |
| Banandur et al – 2011 [59] | 1 | 1 | 1 | 1 | 1 | 1 | 1 | 1 | 1 | 9 | Low |
| Mir et al – 2009 [60] | 1 | 1 | 1 | 1 | 1 | 1 | 1 | 1 | 1 | 9 | Low |
| Johnson et al – 2014 [61] | 1 | 1 | 1 | 1 | 1 | 1 | 1 | 1 | 0 | 8 | Low |
| Sharmin et al – 2021 [62] | 1 | 0 | 1 | 1 | 1 | 1 | 1 | 1 | 1 | 8 | Low |
| Nahar et al – 2014 [63] | 1 | 1 | 1 | 1 | 1 | 1 | 1 | 1 | 1 | 9 | Low |
| Parwez et al – 2022 [64] | 1 | 0 | 1 | 1 | 1 | 1 | 1 | 1 | 1 | 8 | Low |
| Sureshkumar et al – 2015 [65] | 1 | 1 | 1 | 1 | 1 | 1 | 1 | 1 | 1 | 9 | Low |
| Peedicayil et al – 2009 [66] | 1 | 1 | 0 | 1 | 1 | 1 | 1 | 1 | 1 | 8 | Low |
| Datta et al – 2010 [67] | 1 | 1 | 1 | 1 | 1 | 1 | 1 | 1 | 1 | 9 | Low |
| Dutta et al – 2012 [68] | 1 | 0 | 1 | 1 | 1 | 1 | 1 | 1 | 1 | 8 | Low |
| Sherpa et al – 2009 [69] | 1 | 1 | 1 | 1 | 1 | 1 | 1 | 1 | 1 | 9 | Low |
| Thilagavathi et al – 2012 [70] | 0 | 1 | 1 | 1 | 1 | 1 | 1 | 0 | 0 | 6 | Moderate |
| Franceschi et al – 2005 [71] | 1 | 1 | 1 | 1 | 1 | 1 | 1 | 1 | 1 | 9 | Low |
| Mittal et al – 2015 [72] | 1 | 1 | 1 | 1 | 1 | 1 | 1 | 1 | 0 | 8 | Low |
| Silver et al – 2011 [73] | 1 | 0 | 1 | 1 | 1 | 1 | 1 | 1 | 0 | 7 | Moderate |
| Hussain et al – 2012 [74] | 1 | 0 | 1 | 1 | 1 | 1 | 1 | 1 | 1 | 8 | Low |
| Johnson et al – 2016 [75] | 1 | 1 | 1 | 1 | 1 | 1 | 1 | 1 | 1 | 9 | Low |
| Aziz et al – 2023 [76] | 0 | 0 | 1 | 1 | 1 | 1 | 1 | 1 | 0 | 6 | Moderate |
| Shahid et al – 2015 [77] | 0 | 1 | 1 | 1 | 1 | 1 | 1 | 1 | 1 | 8 | Low |
| Baussano et al – 2017 [78] | 1 | 1 | 1 | 1 | 1 | 1 | 1 | 1 | 0 | 8 | Low |
| Becker et al – 2007 [79] | 1 | 1 | 1 | 1 | 1 | 1 | 1 | 1 | 1 | 9 | Low |
| Parvez et al – 2023 [80] | 1 | 1 | 1 | 1 | 1 | 1 | 1 | 1 | 1 | 9 | Low |
| Clifford et al – 2023 [81] | 1 | 1 | 1 | 1 | 1 | 1 | 1 | 1 | 1 | 9 | Low |
| Shakya et al – 2018 [82] | 1 | 1 | 1 | 1 | 1 | 1 | 1 | 1 | 1 | 9 | Low |
| Todd et al – 2012 [83] | 1 | 0 | 1 | 1 | 1 | 1 | 1 | 1 | 1 | 8 | Low |
| Ramesh et al – 2021 [84] | 1 | 1 | 1 | 1 | 1 | 1 | 1 | 1 | 1 | 9 | Low |
| Subramanian et al – 2021 [85] | 1 | 1 | 1 | 1 | 1 | 1 | 1 | 1 | 1 | 9 | Low |
| Dakshinamurthy et al – 2023 [86] | 1 | 0 | 1 | 1 | 1 | 1 | 1 | 1 | 1 | 8 | Low |
| Mishra et al – 2022 [87] | 1 | 0 | 1 | 1 | 1 | 1 | 1 | 1 | 1 | 8 | Low |
| Shashidhar et al – 2021 [88] | 1 | 0 | 0 | 1 | 1 | 1 | 1 | 1 | 1 | 7 | Moderate |
| Bhattacharya et al – 2018 [89] | 1 | 1 | 1 | 1 | 1 | 1 | 1 | 1 | 1 | 9 | Low |
| Asiaf et al – 2012 [90] | 1 | 0 | 1 | 1 | 1 | 1 | 1 | 1 | 1 | 8 | Low |
| Sauvaget et al – 2011 [91] | 1 | 1 | 1 | 1 | 1 | 1 | 1 | 1 | 1 | 9 | Low |
| Sharma et al – 2015 [92] | 1 | 1 | 1 | 1 | 1 | 1 | 1 | 1 | 1 | 9 | Low |
| Srivastava et al – 2012 [93] | 1 | 1 | 1 | 1 | 1 | 1 | 1 | 1 | 1 | 9 | Low |
| Mapitigama et al – 2023 [94] | 1 | 1 | 1 | 1 | 1 | 1 | 1 | 1 | 1 | 9 | Low |
| Vinodhini et al – 2012 [95] | 1 | 0 | 1 | 1 | 1 | 1 | 1 | 1 | 1 | 8 | Low |
| Khanna et al – 2009 [96] | 0 | 0 | 0 | 1 | 1 | 1 | 1 | 1 | 1 | 6 | Moderate |
| Naushad et al – 2017 [97] | 1 | 0 | 1 | 1 | 1 | 1 | 1 | 1 | 1 | 8 | Low |
| Gunasekera et al – 2015 [98] | 1 | 0 | 0 | 1 | 1 | 1 | 1 | 1 | 1 | 7 | Moderate |
| Saranath et al – 2001 [99] | 0 | 0 | 1 | 1 | 1 | 1 | 1 | 1 | 1 | 7 | Moderate |
| Gopalkrishna et al – 2000 [100] | 1 | 0 | 0 | 1 | 1 | 1 | 1 | 1 | 1 | 7 | Moderate |
| Pandit et al – 2013 [101] | 1 | 1 | 1 | 1 | 1 | 1 | 1 | 1 | 1 | 9 | Low |
| Lourembam et al – 2015 [102] | 1 | 1 | 1 | 1 | 1 | 1 | 1 | 1 | 1 | 9 | Low |
| Janani et al – 2015 [103] | 1 | 0 | 0 | 1 | 1 | 1 | 1 | 1 | 1 | 7 | Moderate |
| Janani et al – 2015 [104] | 0 | 0 | 1 | 1 | 1 | 1 | 1 | 1 | 1 | 7 | Moderate |
| Sinha et al – 2015 [105] | 0 | 1 | 1 | 1 | 1 | 1 | 1 | 0 | 1 | 7 | Moderate |
| Ghosh et al – 2014 [106] | 1 | 0 | 1 | 1 | 1 | 1 | 1 | 1 | 1 | 8 | Low |
| Noorali et al – 2004 [107] | 1 | 0 | 0 | 1 | 1 | 1 | 1 | 0 | 1 | 6 | Moderate |
| Borthakur et al – 2016 [108] | 1 | 0 | 0 | 1 | 1 | 1 | 1 | 1 | 1 | 7 | Moderate |
| Chatterjee et al – 2022 [109] | 1 | 1 | 1 | 1 | 1 | 1 | 1 | 1 | 1 | 9 | Low |
| Sangam et al – 2019 [110] | 0 | 0 | 0 | 1 | 1 | 1 | 1 | 1 | 1 | 6 | Moderate |
| Sachithanandham et al – 2013 [111] | 0 | 1 | 1 | 1 | 1 | 1 | 1 | 1 | 1 | 8 | Low |
| Reddy et al – 2016 [112] | 0 | 0 | 0 | 1 | 1 | 1 | 1 | 0 | 1 | 5 | High |
| Sharma et al – 2019 [113] | 1 | 1 | 1 | 1 | 1 | 1 | 1 | 1 | 1 | 9 | Low |
| Rizvi et al – 2011 [114] | 1 | 0 | 1 | 1 | 1 | 1 | 1 | 0 | 1 | 7 | Moderate |
| Husseini et al – 2019 [115] | 1 | 1 | 1 | 1 | 1 | 1 | 1 | 0 | 0 | 7 | Moderate |
| Chakravarti et al – 2010 [116] | 1 | 0 | 0 | 1 | 1 | 1 | 1 | 0 | 1 | 6 | Moderate |
| Das et al – 2014 [117] | 0 | 0 | 1 | 1 | 1 | 1 | 1 | 1 | 1 | 7 | Moderate |
| Chaudhari et al – 2009 [1181] | 0 | 0 | 1 | 1 | 1 | 1 | 1 | 1 | 0 | 6 | Moderate |
| Surpam et al – 2005 [119] | 0 | 0 | 1 | 0 | 1 | 1 | 1 | 0 | 1 | 5 | High |
| Tewari et al – 2011 [120] | 0 | 1 | 1 | 1 | 1 | 1 | 1 | 1 | 1 | 8 | Low |
| Dubey et al – 2020 [121] | 0 | 1 | 0 | 1 | 1 | 1 | 1 | 1 | 1 | 7 | Moderate |
| Anuradha et al – 2011 [122] | 0 | 0 | 0 | 1 | 1 | 1 | 1 | 1 | 1 | 6 | Moderate |
| Mujtaba et al – 2001 [123] | 1 | 0 | 0 | 1 | 1 | 1 | 1 | 1 | 0 | 6 | Moderate |
| Kothari et al – 2002 [124] | 1 | 0 | 1 | 1 | 1 | 1 | 1 | 1 | 0 | 7 | Moderate |
| Kumar et al – 2008 [125] | 0 | 0 | 1 | 1 | 1 | 1 | 1 | 1 | 0 | 6 | Moderate |
| Sharma et al – 2007 [126] | 1 | 1 | 0 | 1 | 1 | 1 | 1 | 0 | 1 | 7 | Moderate |
| Padmavati et al – 2012 [127] | 1 | 0 | 1 | 1 | 1 | 1 | 1 | 1 | 1 | 8 | Low |
| Thapa et al – 2018 [128] | 1 | 1 | 1 | 1 | 1 | 1 | 1 | 1 | 1 | 9 | Low |
| Perera et al – 2021 [129] | 1 | 1 | 1 | 1 | 1 | 1 | 1 | 1 | 1 | 9 | Low |
| Gibney et al – 2001 [130] | 1 | 1 | 1 | 1 | 1 | 1 | 1 | 1 | 1 | 9 | Low |
| Ibrahim et al – 2016 [131] | 1 | 1 | 1 | 1 | 1 | 1 | 1 | 1 | 1 | 9 | Low |
| Hawkes et al – 2002 [132] | 1 | 1 | 1 | 1 | 1 | 1 | 1 | 1 | 1 | 9 | Low |
| Munir et al – 2023 [133] | 1 | 1 | 1 | 1 | 1 | 1 | 1 | 1 | 1 | 9 | Low |
| Perera et al – 2024 [134] | 1 | 1 | 1 | 1 | 1 | 1 | 1 | 1 | 1 | 9 | Low |
| Minhas et al – 2024 [135] | 1 | 1 | 1 | 1 | 1 | 1 | 1 | 1 | 1 | 9 | Low |
| Mittal et al – 2024 [136] | 1 | 1 | 1 | 1 | 1 | 1 | 1 | 1 | 1 | 9 | Low |
| Oommen et al -2024 [137] | 1 | 1 | 1 | 1 | 1 | 1 | 1 | 1 | 1 | 9 | Low |
| Panta et al- 2024 [138] | 1 | 1 | 1 | 1 | 1 | 1 | 1 | 1 | 1 | 9 | Low |
| Parvez et al – 2024 [139] | 1 | 1 | 1 | 1 | 1 | 1 | 1 | 1 | 1 | 9 | Low |
| Munni et al – 2024 [140] | 1 | 1 | 1 | 1 | 1 | 1 | 1 | 1 | 1 | 9 | Low |
| Chakroborty et al – 2024 [141] | 1 | 1 | 1 | 1 | 1 | 1 | 1 | 1 | 1 | 9 | Low |
| Khoja et al – 2024 [142] | 1 | 1 | 1 | 1 | 1 | 1 | 1 | 1 | 1 | 9 | Low |
| Deka et al -2024 [143] | 1 | 1 | 1 | 1 | 1 | 1 | 1 | 1 | 1 | 9 | Low |
