# Supplementary material for: Prevalence of CMV, EBV, HPV, and HSV among South Asian healthy population: A systematic review and meta-analysis
Source: PLOS Glob Public Health. 2026 Jan 7;6(1):e0005728. doi: 10.1371/journal.pgph.0005728 (PMC12779128; doi:10.1371/journal.pgph.0005728)
Supplement: Attachment — Submitted filename: PGPH-D-24-02429_R1.pdf [file pgph.0005728.s013.pdf]

PLOS Global Public Health

Prevalence of CMV, EBV, HPV, and HSV among South Asian Healthy Population: A Systematic Review and Meta-Analysis

--Manuscript Draft--

**Manuscript Number:**	PGPH-D-24-02429
**Article Type:**	Research Article
**Full Title:**	Prevalence of CMV, EBV, HPV, and HSV among South Asian Healthy Population: A Systematic Review and Meta-Analysis
**Short Title:**	Prevalence of CMV, EBV, HPV, and HSV among South Asian Healthy Population
**Corresponding Author:**	Akash Ahmed, MS<br>BRAC University<br>Dhaka, BANGLADESH
**Order of Authors:**	Akash Ahmed, MS
	Rifa Tamanna Subarna
	Zwad Al Saiyan
	Badhan Bhattacharjee
	Nabila Khan
	Nadia Sultana Deen
**Keywords:**	Prevalence; Cytomegalovirus; Epstein-Barr virus; Human Papillomavirus; Herpes simplex virus; South Asia; Healthy population; systematic review
**Abstract:**	Background<br>Cytomegalovirus (CMV), Epstein-Barr virus (EBV), Human papillomavirus (HPV), and Herpes simplex virus (HSV) are DNA viruses with high prevalence rates among the general population. These viruses can cause asymptomatic latent infection resulting in serious health issues such as cancer and autism but there are no studies regarding the pooled prevalence of these viruses among healthy individuals across South Asian countries.<br>Methods<br>A systematic search was performed using three databases (PubMed, Scopus, and Cochrane Library) and one search engine (Google Scholar) for original studies on the South Asian population (published from January 1, 2000, to December 31, 2023). Following the search, a random effect meta-analysis was performed to calculate the overall prevalence of CMV, EBV, HPV, and HSV in South Asia.<br>Results<br>Based on our eligibility criteria, we found 84 studies from 7 South Asian countries comprising 1,56,183 healthy individuals. The overall pooled prevalence of the four viruses was 22% [95% CI: 18% to 27%]. The prevalence of the studies ranged from 0% to 100% indicating a significant amount of heterogeneity (I^2 = 100%; p <0.01). The highest pooled prevalence was of CMV (57%; 95% CI: 21% to 89%) followed by EBV (17%; 95% CI: 5% to 34%), HPV (12%; 95% CI: 10% to 14%), and HSV (8%; 95% CI: 5% to 10%). Furthermore, country-wise analysis showed India to have the majority of the studies.<br>Conclusions<br>Our findings reveal that healthy individuals are infected with at least one of the four DNA viruses, emphasizing the widespread impact across different geographical regions. As these infections can lead to severe health complications, it is crucial to establish preventive guidelines and spread awareness among the healthy population.
**Additional Information:**	
**Question**	**Response**
**Financial Disclosure**	The research project did not get any fund.

Powered by Editorial Manager® and ProduXion Manager® from Aries Systems Corporation

Enter a financial disclosure statement that describes the sources of funding for the work included in this submission and the role the funder(s) played. This includes grants and any commercial funding of the work or authors.<br><br>This statement will be typeset if the manuscript is accepted for publication.<br><br>*Please review the submission guidelines and the instructions link below for detailed requirements and guidance.*	
**Competing Interests**<br><br>On behalf of all authors, disclose any competing interests that could be perceived to bias this work.<br><br>This statement will be typeset if the manuscript is accepted for publication.<br><br>*Please review the instructions link below and PLOS Global Public Health's competing interests policy to determine what information must be disclosed at submission.*	No competing interests to be declared.
**Data Availability**<br><br>Before publication, Authors are required to make fully available and without restriction all data underlying their findings. Please see our PLOS Data Policy page for detailed information on this policy.<br><br>A **Data Availability Statement**, detailing where the data can be accessed, is required at first submission. Insert your Data Availability Statement in the box below.	Raw data is available on reviewer request.

Please see the data reporting section of our submission guidelines for instructions on what you need to include in your Data Availability Statement.<br><br>This statement will be typeset if the manuscript is accepted for publication.<br><br>*PLOS allows rare exemptions to address legal and ethical concerns. If you have legal or ethical restrictions, please use the box below to detail these in full sentences for the Journal team to consider.*	

Powered by Editorial Manager® and ProduXion Manager® from Aries Systems Corporation

Manuscript
Click here to access/download;Manuscript;Corrected_Manuscript_V2.docx

Prevalence of CMV, EBV, HPV, and HSV among South Asian Healthy Population: A Systematic Review and Meta-Analysis

Akash Ahmed[1*], Rifa Tamanna Subarna[1], Zwad Al Saiyan[1], Badhan Bhattacharjee[1], Nabila Khan[1], Nadia Sultana Deen[1]

[1]Microbiology Program, Department of Mathematics & Natural Sciences, Brac University, Dhaka, Bangladesh

*Corresponding author: akash.ahmed@bracu.ac.bd

Abstract

Background

Cytomegalovirus (CMV), Epstein-Barr virus (EBV), Human papillomavirus (HPV), and Herpes simplex virus (HSV) are DNA viruses with high prevalence rates among the general population. These viruses can cause asymptomatic latent infection resulting in serious health issues such as cancer and autism but there are no studies regarding the pooled prevalence of these viruses among healthy individuals across South Asian countries.

Methods

A systematic search was performed using three databases (PubMed, Scopus, and Cochrane Library) and one search engine (Google Scholar) for original studies on the South Asian population (published from January 1, 2000, to December 31, 2023). Following the search, a random effect meta-analysis was performed to calculate the overall prevalence of CMV, EBV, HPV, and HSV in South Asia.

Results

Based on our eligibility criteria, we found 84 studies from 7 South Asian countries comprising ~~1,56,183~~ healthy individuals. The overall pooled prevalence of the four viruses was 22% [95% CI: 18% to 27%]. The prevalence of the studies ranged from 0% to 100% indicating a significant amount of heterogeneity ($I^2 = 100\%$; $p < 0.01$). The highest pooled prevalence was of CMV (57%; 95% CI: 21% to 89%) followed by EBV (17%; 95% CI: 5% to 34%), HPV (12%; 95% CI: 10% to 14%), and HSV (8%; 95% CI: 5% to 10%). Furthermore, country-wise analysis showed India to have the majority of the studies.

Conclusions

Our findings reveal that healthy individuals are infected with at least one of the four DNA viruses, emphasizing the widespread impact across different geographical regions. As these infections can lead to severe health complications, it is crucial to establish preventive guidelines and spread awareness among the healthy population.

Keywords: Prevalence, Cytomegalovirus, Epstein-Barr virus, Human papillomavirus, Herpes simplex virus, South Asia, Healthy population, Systematic review

Author Summary

DNA viruses like Cytomegalovirus (CMV), Epstein-Barr virus (EBV), Human papillomavirus (HPV), and Herpes simplex virus (HSV) are highly prevalent among the general population worldwide. These viruses can cause latent infections that may lead to serious health issues such as cancer and autism. Yet, there is a lack of comprehensive studies on their prevalence among healthy individuals in the South Asian region. We conducted a systematic review and meta-analysis to determine the overall prevalence of CMV, EBV, HPV, and HSV among healthy individuals in South Asia. The overall pooled prevalence of these four viruses was found to be 22% [95% CI: 18% to 27%]. CMV had the highest pooled prevalence at 57% [95% CI: 21% to 89%], followed

by EBV at 17% [95% CI: 5% to 34%], HPV at 12% [95% CI: 10% to 14%], and HSV at 8% [95% CI: 5% to 10%]. These findings underscore the widespread presence of these viruses among healthy individuals in South Asia and highlight the importance of preventive measures and public awareness to mitigate their potential health impacts.

Introduction

Cancer is the leading cause of death globally, where viruses account for 13-20% of all cancer cases [1, 2]. While Epstein-Barr virus (EBV) and Human Papillomavirus (HPV) are established oncoviruses [2], Herpes simplex virus (HSV) and Cytomegalovirus (CMV) are also considered risk factors for cancer [3, 4]. These viruses can directly spread through bodily fluids such as saliva, blood, semen, and breast milk [5-8]. Primary infection with these viruses might be asymptomatic but they can integrate their genetic material into the host chromosome, leading to potential reactivation at any point [9, 10]. This suggests a pressing need for monitoring the prevalence of these viruses among healthy individuals.

Viral cancers typically take 15-40 years to develop, rather than emerging immediately after infection [11]. However, the association between viral infections and cancer development has become increasingly evident through extensive research. For instance, studies have found a high prevalence of CMV in breast, colon and prostate cancer, as well as in hepatocellular carcinoma [12]. Similarly, EBV has been associated with epithelial cancers and a number of lymphoid malignancies such as Burkitt's lymphoma, Hodgkin Lymphoma, and nasopharyngeal carcinoma [13, 14]. It is estimated that EBV is responsible for over a quarter million cases of cancer every year and nearly 2% of all cancer-related deaths are due to EBV-attributed malignancies [15]. In recent years, the connection between HPV, cervical cancer, and several types of squamous cell carcinomas has been well recognised [16]. Studies have found HPV DNA in over 95% of cervical cancers which are the third leading cause of cancer-related deaths among women globally [17].

HSV could be a risk factor for cancer as well since research suggests a potential role of HSV in cervical cancer, where it initiates the oncogenic process [18]. Moreover, according to a Mendelian randomization study, a significant association has been found between HSV infection and an increased risk of head and neck cancer (HNC) [19].

Apart from cancer, these viruses have been linked to neurodevelopmental issues. Viruses, such as CMV [20] and HSV [21], can cross the placental barrier during pregnancy, potentially disrupting brain development and increasing the risk of conditions like autism. Moreover, HPV has been found in placental trophoblasts [22, 23], and its presence has been associated with a greater likelihood of autism spectrum disorders (ASD) [24]. Similarly, EBV has been connected to neurodevelopmental impairments. Studies show that pediatric patients with multiple sclerosis, often positive for EBV, are more likely to experience cognitive challenges compared to their healthy peers [25-27].

The Centers for Disease Control and Prevention (CDC) and The World Health Organization (WHO) have reported a high prevalence of CMV, EBV, HPV, and HSV among the general population [28-31]. However, these prevalence rates differ among geographic regions. For example, in the United States, the prevalence rates are 50% (CMV) [32], 66.5% (EBV) [33], 40% (HPV) [34], and 12.1-48.1% (HSV) [35, 36] whereas Africa shows notable variation, with lower rates for EBV (20%) [37] and HPV (2-45%) [38] but higher rates for HSV (37.3-90%) [39, 40], and CMV (81.8%) [41]. Although extensive prevalence data for CMV, EBV, HPV, and HSV are available for the aforementioned regions, comprehensive prevalence studies for these four viruses are limited in South Asia.

South Asia consists of eight countries: Afghanistan, Bangladesh, Bhutan, India, Maldives, Nepal, Pakistan, and Sri Lanka [42]. These nations collectively have a population of approximately 2.4 billion people, accounting for more than one-fourth of the global population [43]. Various studies

have highlighted the prevalence of CMV, EBV, HPV, and HSV in these populations. For example, HPV prevalence among South Indian cervical cancer patients is 94% [44], while EBV prevalence in Bangladeshi Hodgkin Lymphoma patients is 68.1% [45]. Furthermore, among pregnant women in India, CMV and HSV prevalence rates are 8% and 6%, respectively [46]. The positive prevalence of these viral infections among these populations could indicate the presence of latent oncogenes, which may expose many individuals to an increased risk of cancer and autism. Therefore, a synthesis of these prevalence data would be important for treating asymptomatic CMV, EBV, HPV, and HSV infections as persistent contributors to the emergence of cancer-causing mutant genes rather than as latent infections.

This systematic review and meta-analysis aimed to estimate a pooled prevalence of CMV, EBV, HSV, and HPV among the healthy population of South Asia. Meta-analysis was done to explore the different prevalence rates of the four viruses. Country wise prevalence were also examined for a comprehensive understanding of the distribution and potential demographic disparities in the region. This prevalence data is crucial for informing policymakers and healthcare professionals to implement necessary measures aimed at reducing the transmission and risk associated with these DNA viruses.

Methods

We followed the latest recommendations and guidelines from the Preferred Reporting Items for Systematic Review and Meta-Analysis (PRISMA 2020) [47]. Additionally, this systematic review was registered in PROSPERO (CRD42024510467).

Data source and search strategy

The initial search was conducted by one of the co-authors (AKS) to conceptualize the idea and identify the knowledge gap. Afterward, the eligibility criteria were developed by all the co-authors.

Three co-authors (RTS, ZAS, and NMN) systematically searched articles indexed in PubMed, Scopus, Cochrane Library, and Google Scholar. The searches were performed independently (from 17th November 2023 to 20th February 2024), after logging out from all Google accounts. The search strategy aimed to retrieve articles published between 1st January 2000 and 31st December 2023, focusing on the prevalence of CMV, EBV, HPV, and HSV among healthy individuals in South Asian countries.

Our search terms included combinations of keywords [e.g., "Herpes simplex virus," "Human Papillomavirus," "Cytomegalovirus," "EBV," "HSV", "HPV", "CMV", "Seroprevalence", "Incidence," "Prevalence," "Frequency" "Distribution", "Healthy", "South Asia," "Bangladesh," "India," "Pakistan," "Nepal," "Sri Lanka," "Bhutan," "Maldives," and "Afghanistan"] to cover all the relevant articles as per our eligibility criteria. The detailed search strategy, list of original MeSH terms, and alternative terms used in this study are given in the S1 Table.

To maximize the search efficiency, we also went through similar articles suggested by the World Health Organization (WHO) and the Centers for Disease Control and Prevention (CDC) websites to gather relevant articles on CMV, EBV, HPV, and HSV.

Eligibility criteria

We selected articles that fulfilled the following inclusion criteria: 1. Studies published in English, with full-text availability, and reporting the prevalence of any of these viruses CMV, EBV, HPV, and HSV; 2. Original, peer-reviewed observational studies (cross-sectional, retrospective, prospective, pilot, and case-control studies using only the control groups); 3. Healthy individuals (with minor illnesses unrelated to chronic diseases); 4. Conducted in South Asian countries (Afghanistan, Bangladesh, Bhutan, India, Maldives, Nepal, Pakistan, and Sri Lanka); 5. Articles published from 2000 to 2023.

Conversely, the following were the exclusion criteria: 1. Secondary sources (Review articles, letters to the editor, and studies published before 2000); 2. Individuals with chronic medical conditions (Cancer, chronic kidney disease, coronary artery disease, HIV) or group of population (pregnant women, and sex workers); 3. Studies that did not report the prevalence of viruses; 4. Studies that met the inclusion criteria but the full text could not be retrieved from the authors after requests.

The Mendeley Desktop software (version 1.19.4) was used to organize the references and remove the duplicates. Studies were independently verified by two co-authors (AKS and BB) before final inclusion in the meta-analysis. Disagreements were resolved through group discussions between all co-authors.

Data extraction

Three of the co-authors independently used an Excel table to systematically extract information from all eligible studies. The information that was extracted for each study were: publication details [e.g., first author, publication date]; study setting (community, population, or hospital-based); population and study design [e.g., country, study location, study period, and sample size]; ethical statements and consent (written, oral, or verbal), characteristics of participants [e.g., gender, age] and key findings (e.g., testing methods, sample type, and viral prevalence). The accuracy of the extracted data was verified by all co-authors through multiple revisions of the included studies. In cases where viral prevalence was reported as a percentage, conversions to decimal format were performed to ensure consistency across the dataset.

Evaluation of study quality

Study quality was evaluated by following the 9-item tool explicitly developed by Joanna Briggs Institute for prevalence study [48]. Based on the critical appraisal tool scoring system, if the answer

was "Yes", then 1 was implied and if the answer was "No" or "Unclear" then 0 was implied. Each study was categorized based on its total score as either "low-risk bias" (8-9), "moderate risk bias" (6-7), or "high-risk bias" (0-5). Quality assessment was conducted for all selected articles independently by two co-authors (RTS, ZAS). Disagreements among co-authors (RTS, ZAS) during the assessment were resolved through discussions with the project supervisor (AKS) to uphold consistency.

Statistical analysis

The prevalence of CMV, EBV, HPV, and HSV were considered as summary measurements. We used a random-effect model to obtain pooled prevalence with a 95% confidence interval. Country-wise and gender-wise analysis was also performed according to the prevalence of the participants. Heterogeneity was assessed using Cochran's Q test and the I^2 statistics. Substantial heterogeneity was indicated with an I^2 of more than 75% [49]. Publication bias was also examined using a funnel plot. All statistical analyses were performed using Microsoft Excel version 16.

Result

We found 6,307 studies using the search strategy mentioned earlier. Among these, 6,223 articles were excluded, and finally, 84 studies comprising 1, 56,183 healthy individuals were included in this systematic review and meta-analysis [50-133]. The details of the study articles selection process are shown in Fig 1.

Out of 84 studies, 61 were conducted in India with a population of 13, 4079. Among the rest, 7 were conducted in Pakistan (3,125 participants), 5 in Nepal (3,711 participants), 4 in Bangladesh (3,011 individuals), 3 in Sri Lanka (1,885 individuals), 2 in Bhutan (5,137 individuals), and 2 in Afghanistan (5,235 individuals). We found that more than half of the studies were hospital-based 44 out of 84), and the rest were community-based (39 out of 84). Additionally, 1 did not specify

the study setting. Nearly half of the studies did not mention their study design (33 out of 84) [59, 67-74, 76, 79, 82, 86, 89, 90, 93, 96, 97, 99-101, 107, 108, 111, 114-116, 119, 122-124, 126, 127] and the remaining studies consisted of cross-sectional (37 out of 84), case-control (11 out of 84), and cohort (3 out of 84). To detect the presence of CMV, EBV, HPV, and HSV the studies used several kinds of testing methods. Among these polymerase chain reaction (PCR) (40 out of 84) and enzyme-linked immunosorbent assay (ELISA) (30 out of 84) were mostly used, only 3 studies mentioned the use of both PCR and ELISA. Other methods included Hybrid-capture 2 (HC2) (3 out of 84), APTIMA HPV (2 out of 84), CareHPV Test (2 out of 84), immunohistochemistry (IHC) (2 out of 84), and chemiluminescence immunoassay (CLIA) (1 out of 84) and Southern blot (1 out of 84). A maximum number of studies used blood samples (42 out of 84) for detecting viral infection, while other studies utilized cervical specimens (32 out of 84), urine (5 out of 84), tissue (2 out of 84), oral specimens (2 out of 84) or normal mucosa (1 out of 84). A table summarizing the characteristics of the selected studies is presented in Table 1.

The overall pooled prevalence of these viruses was 22% [95% CI: 18% to 27%]. There was a significant amount of heterogeneity in the prevalence of the 4 viruses ($I^2 = 100\%$; $p<0.01$). The overall prevalence is shown in the Forest plot (Fig 2).

Analysis according to virus

This study included the prevalence of four viruses (CMV, EBV, HPV, and HSV) among healthy populations in seven South Asian countries: Afghanistan, Bangladesh, Bhutan, India, Pakistan, Nepal, and Sri Lanka. No studies were found from the Maldives regarding the prevalence of any of these viruses.

CMV

We retrieved 17 studies, with a total of 12,453 healthy individuals reporting the prevalence of CMV infection. Country-wise analysis showed that most of the studies were conducted in India (15 out of 17) with a pooled prevalence of 48% [95% CI: 12% to 85%] (S1 Fig). Single studies were conducted in Afghanistan and Pakistan (prevalence ranging from 91% to 100%). All these studies were either hospital-based (11 out of 17) or community-based (5 out of 17), with 1 study did not mention the study setting. The study designs was either cross-sectional (5 out of 17), or case-control (2 out of 17). Most of the studies did not mention study design (10 out of 17). A larger number of studies did not have gender-specific prevalence, while 2 studies were conducted only in males [132, 133]. The pooled prevalence of CMV was 57% [95% CI: 21% to 89%] with a significant amount of heterogeneity ($I^2 = 100\%$; $p<0.01$) (Fig 3).

EBV

We found 18 studies involving 3,914 healthy individuals reporting on the prevalence of EBV infection. Among them, 15 studies were carried out in India with a prevalence of 22% [95% CI: 8%-40%] (S2 Fig), and the remaining 3 studies were conducted in Pakistan (prevalence ranging from 0% to 16%). Most of the studies were hospital-based (16 out of 18), while 2 were community-based. The study designs varied, including cross-sectional (5 out of 18), case-control (6 out of 18), and not mentioned (7 out of 18). The majority of the studies included both male and female participants, while 3 studies were only conducted on males. The overall prevalence of EBV was 17% [95% CI: 5% to 34%] with a high degree of heterogeneity ($I^2 =99\%$; $p<0.01$) (Fig 4).

HPV

We found 42 studies comprising ~~1, 05, 205~~ healthy individuals that reported HPV infection. The majority of these studies were conducted in India (27 out of 42), revealing a pooled prevalence rate of 13% [95% CI: 10% to 16%] (S3 Fig). Furthermore, we found 5 studies from Nepal

(prevalence ranging from 5% to 22%), 2 from Bhutan (prevalence ranging from 9% to 11%), 2 from Bangladesh (prevalence ranging from 6% to 34%), 3 from Pakistan (prevalence ranging from 0% to 16%), and 3 from Sri Lanka (prevalence ranging from 4% to 25%). The study settings of these studies were either hospital-based (17 out of 42) or community-based (25 out of 42). While most of the studies were cross-sectional (18 out of 42), the remaining were case-control (3 out of 42), cohort (3 out of 42) or not mentioned (18 out of 42). Out of 42 studies, 37 studies were conducted only on female participants with a prevalence rate of 12% [95% CI: 10% to 14%] (S4 Fig) while the rest included both males and females. The overall prevalence of HPV infection was 12% [95% CI: 10% to 14%] with a significant heterogeneity ($I^2 = 99\%$; $p<0.01$) (Fig 5).

HSV

We found 17 studies reporting the prevalence of HSV infection, involving a total of 42,688 healthy individuals. Among these, 13 studies were conducted in India with a pooled prevalence of 8% [95% CI: 5% to 10%] (S5 Fig), while 2 were conducted in Bangladesh (prevalence ranging from 3% to 30%), and single studies were conducted in Afghanistan and Pakistan (prevalence ranging from 3% to 4%). The majority of the study settings were either hospital-based (3 out of 17) or community-based (13 out of 17) and 1 study did not specify the study setting. The study designs included cross-sectional (12 out of 17), case-control (2 out of 17) and not mentioned (3 out of 17). Out of these studies, 5 focused solely on male participants with a prevalence of 10% [95% CI: 6% to 14%] (S6 Fig), and 4 studies on females with a prevalence rate of 8% [95% CI: 5% to 12%] (S7 Fig). The overall prevalence of HSV infection was 8% [95% CI: 5% to 10%] with a significant amount of heterogeneity ($I^2 = 100\%$; $p<0.01$) (Fig 6).

Quality Assessment and Publication bias

Critical Appraisal Tool for prevalence study developed by the Joanna Briggs Institute was used for the evaluation of selected study articles. Out of 84 selected articles, 57 showed a low risk of

bias, 25 had a moderate risk of bias, and only 2 were found to contain a high risk of bias. Additionally, funnel plots indicating the existence of asymmetry and publication bias for the overall prevalence of these viruses (HSV, HPV, CMV, and EBV) are presented in a supporting file; S6 Fig.

Discussion

Our findings highlighted that the overall pooled prevalence of these viruses is 22% [95% CI: 18% to 27%]. Among these four viruses, CMV infection had the highest prevalence 57% [95% CI: 21% to 89%] and HSV had the lowest 8% [95% CI: 5% to 10%]. This result indicates that compared to the other viruses, CMV transmission is more widespread in South Asia, highlighting the need for targeted interventions and further research into the factors contributing to its high prevalence. Our result also indicates that any healthy individual is likely to encounter one of these viruses and develop a lifelong infection due to their latent infectious capabilities. Therefore, a comprehensive approach is essential to accurately determine the burden of these viruses in South Asia and design targeted public health strategies.

A study conducted in Poland reported the prevalence of the same four viruses and found that 30% of healthy individuals were infected with at least one of them [134]. The relatively lower prevalence in our study could be attributed to the diverse sample sizes across South Asian countries, in contrast to the smaller and more homogeneous samples from Poland. Additionally, our study showed that in India the prevalence of CMV, EBV, HPV, and HSV was 48%, 22%, 13%, and 8%, respectively. When compared with similar studies across the globe, CMV shows a prevalence of 86% in the WHO Southeast Asian region among the general population and EBV shows a prevalence of 95.4% among healthy adults in China [135, 136]. Our estimate for HPV aligns more closely with Northern American women with normal cytology (11.3%) whereas Europe shows a lower prevalence (8.1%) [137]. In Australia, a nationwide population survey

reported a broader range of HSV prevalence (12-76%) compared to our findings [138]. These variations highlight regional differences that might be influenced by factors such as healthcare access, and cultural practices. Studies also support this argument by demonstrating that regional disparities in viral infections can arise from cultural norms that influence vaccination intentions [139], as well as from inequalities in healthcare access, including insurance coverage and regular medical care [140].

A concerning finding from our study was that among healthy females in South Asia, the prevalence of HPV was 12%, which contradicts the 4.4% prevalence reported in individuals with normal cytology in the same region according to the Human Papillomavirus and Related Diseases Report [141]. A plausible explanation might be that healthy females in the South Asian general population might not have undergone regular screening for HPV. As a result, a higher prevalence was observed when they were eventually tested. Another gender-specific finding from our study was that HSV prevalence was higher among men (10%) compared to women (8%), which contrasts with the reported fact that HSV infection is typically twice as common in women compared to men [8]. This difference could be attributed to socio-cultural factors, such as socio-economic conditions and cultural behaviors within specific racial or educational groups [142-144], influencing the varying rates of HSV infection [145].

Although to the best of our knowledge, this is the first systematic review and meta-analysis that combined determines the prevalence of CMV, EBV, HPV, and HSV in the South Asian healthy population, the study also has few limitations. We found that a large number of included studies were conducted in India and there were no studies from Maldives suggesting the need for conducting similar research across other South Asian countries. Also, most of the studies included in this review were female-focused, so we could not perform gender-based meta-analysis for all the viruses. Moreover, data for types of viruses were not available in all the

studies which restricted us from doing analysis on virus types. These factors reflected a lack of sub-group analysis in our systematic review. In addition, our meta-analysis showed substantial heterogeneity (I^2 = 100%; p<0.01) due to a number of factors. Some of our included studies demonstrated 0% [84, 97, 105, 107, 108, 111, 114, 125] and 100% [92, 115] prevalence rates which is mainly due to variations in sample size, study design, and the specific populations being studied. These variabilities influenced the overall weighting of prevalence rates in our statistical analysis. Lastly, using different methods for detecting the viral infection across different studies could introduce assay bias to our findings. However, this limitation is inherent in all similar studies and was, therefore, unavoidable.

The ability of these viruses to persist and reactivate in the host poses a significant challenge for healthcare, necessitating ongoing research into prevention and treatment strategies. For CMV, prevention of congenital infection involves educating pregnant women about transmission risks and promoting hygienic practices. Currently, there are no established antiviral treatments or vaccines for CMV [146]. However, recent advances include the development of replication-defective virus vaccines, which have shown promising immune responses in phase 1 trials [147]. For EBV, prevention primarily focuses on reducing the risk of transmission through measures such as avoiding intimate contact with individuals shedding the virus, particularly during periods of illness, and promoting good hygiene practices [148]. HPV prevention efforts are largely centered on vaccination, targeting young adolescents to reduce the transmission and subsequent development of HPV-related cancers. Additionally, regular screening and treatment of precancerous lesions in women are crucial components of HPV control programs [149]. In the case of HSV, preventive measures involve educating individuals about transmission risks, encouraging disclosure of infection status to partners, practicing safe sex, and considering suppressive therapy in certain cases [150]. Overall, a comprehensive approach to prevention involves a combination of vaccination, education, behavioral interventions, and public health

initiatives aimed at reducing transmission and mitigating the burden of these viral infections on global health.

It is estimated that, on average, every person can be simultaneously infected with 8 to 12 chronic viral infections, whether caused by DNA or RNA viruses [151]. While EBV and CMV can mimic the common cold, HPV and HSV can cause painless lumps or be completely asymptomatic [152-155]. Recent reports have confirmed that after having infections with CMV, and EBV people can develop Guillain-Barré syndrome (GBS) [156]. Moreover, infections with HPV and HSV have detrimental consequences especially in women as HPV infection can lead to cervical cancer and HSV can be transmitted from pregnant women to their neonates [157, 158]. Hence, in our study, the prevalence rates of the four DNA (CMV, EBV, HPV, and HSV) viruses suggest the need for regular medical screening.

In conclusion, our study confirms that 22 out of 100 healthy individuals are infected with one of these DNA viruses, emphasizing the widespread impact across different geographical regions in South Asia. Hence, it is crucial to establish preventive guidelines and spread awareness, as these infections can lead to severe health complications. The findings of our study, while offering insights into the prevalence of four viruses in South Asia, can be implemented in public health strategies globally.

Supporting information

S1 Table. Search strategy used in each database. (DOCX)

S2 Table. Characteristics of the selected studies. (DOCX)

S3 Table. Quality assessment of selected studies. (DOCX)

S1 Fig. Forest plot of CMV infection in India.

S2 Fig. Forest plot of EBV infection in India.

S3 Fig. Forest plot of HPV infection in India.

S4 Fig. Forest plot of HPV infection in the female population.

S5 Fig. Forest plot of HSV infection in India.

S6 Fig. Forest plot of HSV infection in the male population.

S7 Fig. Forest plot of HSV infection in the female population.

Acknowledgments

Author Contributions

Conceptualization: Akash Ahmed

Data curation:Nabila Khan, Rifa Tamanna Subarna & Zwad Al Saiyan

Formal analysis: Rifa Tamanna Subarna, Zwad Al Saiyan & Badhan Bhattachariya

Investigation: Rifa Tamanna Subarna, Zwad Al Saiyan & Nabila Khan

Methodology: Zwad Al Saiyan, Rifa Tamanna Subarna & Nabila Khan

Project administration: Akash Ahmed

Software: Zwad Al Saiyan & Badhan Bhattachariya

Supervision: Akash Ahmed

Visualization: Nadia Sulatana Deen, Zwad Al Saiyan & Nabila Khan

Writing – original draft: Akash Ahmed, Rifa Tamanna Subarna

Writing – review & editing: Akash Ahmed, Nadia Sulatana Deen & Badhan Bhattachariya

References

1 World Health Organization. Cancer. February 3, 2022. Available from: https://www.who.int/news-room/fact-sheets/detail/cancer. Accessed June 29, 2024.

2 Mui UN, Haley CT, Tyring SK. Viral oncology: Molecular biology and pathogenesis. In: Journal of Clinical Medicine, vol. 6. MDPI; 2017. doi: 10.3390/jcm6120111.

3 Sausen DG, Shechter O, Gallo ES, Dahari H, Borenstein R. Herpes Simplex Virus, Human Papillomavirus, and Cervical Cancer: Overview, Relationship, and Treatment Implications. Cancers. 2023;15(1):23. doi:10.3390/cancers15143692.

4 Yu C, He S, Zhu W, Ru P, Ge X, Govindasamy K. Human cytomegalovirus in cancer: the mechanism of HCMV-induced carcinogenesis and its therapeutic potential. Front Cell Infect Microbiol. 2023;13:123. doi: 10.3389/fcimb.2023.1202138.

5 About Epstein-Barr virus (EBV). Epstein-Barr Virus and Infectious Mononucleosis. May 9, 2024. Available from: https://www.cdc.gov/epstein-barr/about/?CDC_AAref_Val=https://www.cdc.gov/epstein-barr/about-ebv.html. Accessed June 29, 2024.

6 Clinical overview of CMV and congenital CMV. Cytomegalovirus (CMV) and Congenital CMV Infection. April 5, 2024. Available from: https://www.cdc.gov/cytomegalovirus/hcp/clinical-overview/?CDC_AAref_Val=https://www.cdc.gov/cmv/clinical/overview.html. Accessed June 29, 2024.

7 About genital HPV infection. Sexually Transmitted Infections (STIs). February 6, 2024. Available from: https://www.cdc.gov/sti/about/about-genital-hpv-

infection.html?CDC_AAref_Val=https://www.cdc.gov/std/hpv/stdfact-hpv.htm. Accessed June 29, 2024.

8 World Health Organization. Herpes simplex virus. April 5, 2023. Available from: https://www.who.int/news-room/fact-sheets/detail/herpes-simplex-virus. Accessed June 29, 2024.

9 Potasman I. Asymptomatic Infections: The Hidden Epidemic. Int J Clin Res Trials. 2017 Nov 22;2(2). doi: 10.15344/2456-8007/2017/118.

10 Traylen CM, Patel HR, Fondaw W, Mahatme S, Williams JF, Walker LR, et al. Virus reactivation: A panoramic view in human infections. Future Virol. 2011;6:451-63. doi: 10.2217/fvl.11.21.

11 Hausen HZ. The search for infectious causes of human cancers: Where and why. Virology. 2009;392(1):1-10. doi: 10.1016/j.virol.2009.06.001.

12 Nauclér CS, Geisler J, Vetvik K. The emerging role of human cytomegalovirus infection in human carcinogenesis: a review of current evidence and potential therapeutic implications. Oncotarget. 2019;10(42):4333-47. doi: 10.18632/oncotarget.27016.

13 Farrell PJ. Epstein–Barr virus and cancer. Annu Rev Pathol Mech Dis. 2019;14(1):29-53. doi: 10.1146/annurev-pathmechdis-012418-013023.

14 Thorley-Lawson DA, Gross A. Persistence of the Epstein–Barr virus and the origins of associated lymphomas. N Engl J Med. 2004;350(13):1328-37. doi: 10.1056/nejmra032015.

15 Yu H, Robertson ES. Epstein–Barr virus history and pathogenesis. Viruses. 2023;15(3):714. doi: 10.3390/v15030714.

16 Cubie HA. Diseases associated with human papillomavirus infection. Virology. 2013;445(1-2):21-34. doi: 10.1016/j.virol.2013.06.007.

17 Hebner CM, Laimins LA. Human papillomaviruses: basic mechanisms of pathogenesis and oncogenicity. Rev Med Virol. 2005;16(2):83-97. doi: 10.1002/rmv.488.

18 Sausen D, Shechter O, Gallo E, Dahari H, Borenstein R. Herpes simplex virus, human papillomavirus, and cervical cancer: Overview, relationship, and treatment implications. Cancers. 2023;15(14):3692. doi: 10.3390/cancers15143692.

19 Yan M, Xiao L, Gosau M, Smeets R, Feng H, Burg S, et al. The role of herpes simplex virus infection in the etiology of head and neck cancer–a Mendelian randomization study. Front Immunol. 2024;15:1278327. doi: 10.3389/fimmu.2024.1278327.

20 Cordeiro CN, Tsimis M, Burd I. Infections and brain development. Obstet Gynecol Surv. 2015;70(10):644-55. doi: 10.1097/ogx.0000000000000236.

21 Al-Beltagi M, Saeed NK, Elbeltagi R, Bediwy AS, Aftab SaS, Alhawamdeh R. Viruses and autism: A bi-mutual cause and effect. World J Virol. 2023;12(3):172-92. doi: 10.5501/wjv.v12.i3.172.

22 You H, Liu Y, Agrawal N, Prasad CK, Edwards JL, Osborne AF, et al. Multiple human papillomavirus types replicate in 3A trophoblasts. Placenta. 2008;29(1):30-8. doi: 10.1016/j.placenta.2007.08.005.

23 Walker CK, Anderson KW, Milano KM, Ye S, Tancredi DJ, Pessah IN, et al. Trophoblast inclusions are significantly increased in the placentas of children in families at risk for autism. Biol Psychiatry. 2013;74(3):204-11. doi: 10.1016/j.biopsych.2013.03.006.

24 Godar DE, Merrill SJ. Untangling the most probable role for vitamin D3 in autism. Dermatoendocrinol. 2017;9(1). doi: 10.1080/19381980.2017.1387702.

25 Alotaibi S. Epstein-Barr virus in pediatric multiple sclerosis. JAMA. 2004;291(15):1875. doi: 10.1001/jama.291.15.1875.

26 Houen G, Trier NH, Frederiksen JL. Epstein-Barr virus and multiple sclerosis. Front Immunol. 2020;11:587078. doi: 10.3389/fimmu.2020.587078.

27 Ekmekci O. Pediatric multiple sclerosis and cognition: A review of clinical, neuropsychologic, and neuroradiologic features. Behav Neurol. 2017;2017:1-11. doi: 10.1155/2017/1463570.

28 Clinical overview of CMV and congenital CMV. Cytomegalovirus (CMV) and Congenital CMV Infection. 2024 Apr 5. Available from: https://www.cdc.gov/cytomegalovirus/hcp/clinical-overview/index.html. Accessed June 29, 2024.

29 About Epstein-Barr virus (EBV). Epstein-Barr Virus and Infectious Mononucleosis. 2024 May 9. Available from: https://www.cdc.gov/epstein-barr/about/index.html. Accessed June 29, 2024.

30 World Health Organization. Sexually transmitted infections (STIs). May 21, 2024. Available from: https://www.who.int/news-room/fact-sheets/detail/sexually-transmitted-infections-(stis)?gad_source=1&gclid=Cj0KCQjwxqayBhDFARIsAANWRnTiy9f6AIKXgEkEYMr4ohq2ffmHcHF5RPSp1z397WOT2WadLLMky_8aArjPEALw_wcB. Accessed June 29, 2024.

31 World Health Organization. Herpes simplex virus. 2023 Apr 5. Available from: https://www.who.int/news-room/fact-sheets/detail/herpes-simplex-virus. Accessed June 29, 2024.

32 Centers for Disease Control and Prevention. About cytomegalovirus. Cytomegalovirus (CMV) and congenital CMV infection. [Internet]. Available from: https://www.cdc.gov/cytomegalovirus/about/index.html#:~:text=CMV%20is%20a%20common%20virus,for%20life%20and%20can%20reactivate

33 Dowd JB, Palermo T, Brite J, McDade TW, Aiello A. Seroprevalence of Epstein-Barr Virus Infection in U.S. Children Ages 6-19, 2003-2010. PLoS One. 2013 May 22;8(5). doi: 10.1371/journal.pone.0064921.

34 Lewis RM, Laprise JF, Gargano JW, Unger ER, Querec TD, Chesson HW, et al. Estimated prevalence and incidence of disease-associated human papillomavirus types among 15- to 59-year-olds in the United States. Sex Transm Dis. 2021 Apr 1;48(4):273-277. doi: 10.1097/OLQ.0000000000001356.

35 Tayyar R, Ho D. Herpes Simplex Virus and Varicella Zoster Virus Infections in Cancer Patients. Viruses. 2023;15(1). doi: 10.3390/v15020439.

36 McQuillan G, Kruszon-Moran D, Flagg EW, Paulose-Ram R. Prevalence of Herpes Simplex Virus Type 1 and Type 2 in Persons Aged Key findings Data from the National Health and Nutrition Examination Survey [Internet]. 2015. Available from: https://www.cdc.gov/nchs/data/databriefs/db304_table.pdf#3. Accessed June 29, 2024.

37 Adjei AA, Armah HB, Gbagbo F, Boamah I, Adu-Gyamfi C, Asare I. Seroprevalence of HHV-8, CMV, and EBV among the general population in Ghana, West Africa. BMC Infect Dis. 2008 Aug 18;8:111. doi: 10.1186/1471-2334-8-111.

38 Kombe AJK, Li B, Zahid A, Mengist HM, Bounda G, Zhou Y, et al. Epidemiology and burden of human papillomavirus and related diseases, molecular pathogenesis, and vaccine evaluation. Front Public Health. 2021;8:552028. doi: 10.3389/fpubh.2020.552028.

39 Harfouche M, Abu-Hijleh FM, James C, Looker KJ, Abu-Raddad LJ. Epidemiology of herpes simplex virus type 2 in sub-Saharan Africa: Systematic review, meta-analyses, and meta-regressions. EClinicalMedicine. 2021 May 1;35. doi: 10.1016/j.eclinm.2021.100876.

40 Harfouche M, Chemaitelly H, Abu-Raddad LJ. Herpes simplex virus type 1 epidemiology in Africa: Systematic review, meta-analyses, and meta-regressions. J Infect. 2019 Oct 1;79(4):289-299. doi: 10.1016/j.jinf.2019.07.012.

41 Bates M, Brantsaeter AB. Human cytomegalovirus (CMV) in Africa: a neglected but important pathogen. 2016. doi: 10.5772/54907.

42 South Asia. World Bank. Available from: https://www.worldbank.org/en/region/sar. Accessed June 29, 2024.

43 Population of Southern Asia (2024) - Worldometer. Worldometer. Available from: https://www.worldometers.info/world-population/southern-asia-population/. Accessed June 29, 2024.

44 Baskran K, Kumar PK, Santha K, Sivakamasundari II. Cofactors and their association with cancer of the uterine cervix in women infected with high-risk human papillomavirus in South India. Asian Pac J Cancer Prev. 2019;20(11):3415-9. doi: 10.31557/apjcp.2019.20.11.3415.

45 Hashmi AA, Hussain ZF, Hashmi KA, Zafar MI, Edhi MM, Faridi N, et al. Latent membrane protein 1 (LMP1) expression in Hodgkin lymphoma and its correlation with clinical and histologic parameters. World J Surg Oncol. 2017;15(1):60. doi: 10.1186/s12957-017-1147-y.

46 Tanneeru S, Mahendra J, Shaik MV. Evaluation of microflora (viral and bacterial) in subgingival and placental samples of pregnant women with preeclampsia with and without

periodontal disease: A cross-sectional study. J Int Soc Prev Community Dent. 2020;10(2):171-9. doi: 10.4103/jispcd.jispcd_341_19.

47 Page MJ, McKenzie JE, Bossuyt PM, Boutron I, Hoffmann TC, Mulrow CD, et al. The PRISMA 2020 statement: An updated guideline for reporting systematic reviews. BMJ. 2021;372. doi: 10.1136/bmj.n71.

48 Munn Z, Moola S, Riitano D, Lisy K. The development of a critical appraisal tool for use in systematic reviews addressing questions of prevalence. Int J Health Policy Manag. 2014;3(3):123-128. doi: 10.15171/ijhpm.2014.71.

49 Higgins JPT, Thompson SG, Deeks JJ, Altman DG. Measuring inconsistency in meta-analyses Testing for heterogeneity. BMJ. 2003;327(7414):557-560. doi: 10.1136/bmj.327.7414.557.

50 Schneider JA, Lakshmi V, Dandona R, Kumar GA, Sudha T, Dandona L. Population-based seroprevalence of HSV-2 and syphilis in Andhra Pradesh state of India. BMC Infect Dis. 2010;10(1):59. doi: 10.1186/1471-2334-10-59.

51 Ray K, Bala M, Bhattacharya M, Muralidhar S, Kumari M, Salhan S. Prevalence of RTI/STI agents and HIV infection in symptomatic and asymptomatic women attending peripheral health set-ups in Delhi, India. Epidemiol Infect. 2008;136(10):1432-1440. doi: 10.1017/S0950268807000088.

52 Sgaier SK, Mony P, Jayakumar S, McLaughlin C, Arora P, Kumar R, et al. Prevalence and correlates of Herpes Simplex Virus-2 and syphilis infections in the general population in India. Sex Transm Infect. 2011 Mar;87(2):94-100. doi: 10.1136/sti.2010.043687.

53 Dave SS, Copas A, Richens J, White RG, Kosambiya JK, Desai VK, et al. HIV and STI prevalence and determinants among male migrant workers in India. PLoS One. 2012 Aug 27;7(8). doi: 10.1371/journal.pone.0043576.

54 Ghosh S, Shetty RS, Pattanshetty SM, Mallya SD, Pandey D, Kabekkodu SP, et al. Human papilloma and other DNA virus infections of the cervix: A population based comparative study among tribal and general population in India. PLoS One. 2019 Jun 1;14(6). doi: 10.1371/journal.pone.0219173.

55 Adamson PC, Krupp K, Freeman AH, Klausner JD, Reingold AL, Madhivanan P. Prevalence & correlates of primary infertility among young women in Mysore, India. PubMed Central (PMC). Published October 1, 2011. PMID: 22089604.

56 Panchanadeswaran S, Johnson SC, Mayer KH, Srikrishnan AK, Sivaram S, Zelaya CE, et al. Gender differences in the prevalence of sexually transmitted infections and genital symptoms in an urban setting in southern India. Sex Transm Infect. 2006 Dec;82(6):491–5. doi: 10.1136/sti.2006.020768.

57 Schensul SL, Hawkes S, Saggurti N, Verma RK, Narvekar SS, Nastasi BK, et al. Sexually transmitted infections in men in Mumbai slum communities: The relationship of prevalence to risk behavior. Sex Transm Dis. 2007 Jul;34(7):444–50. doi: 10.1097/01.olq.0000249776.92490.32.

58 Patil S, Rao A, Pathak P, Kurle S, Mane A, Nirmalkar A, et al. Unsterile injection equipment associated with HIV outbreak and an extremely high prevalence of HCV—A case-control investigation from Unnao, India. PLoS One. 2020 Dec 1;15(12 December). doi: 10.1371/journal.pone.0243534.

59 Banandur P, Rajaram S, Mahagaonkar SB, Bradley J, Ramesh BM, Washington RG, Blanchard JF, Moses S, Lowndes CM, Alary M. Heterogeneity of the HIV epidemic in the general population of Karnataka state, south India. BMC Public Health. 2011;11(Suppl 6). doi: 10.1186/1471-2458-11-S6-S13.

60 Mir AM, Wajid A, Reichenbach L, Khan M. STI prevalence and associated factors among urban men in Pakistan. Sex Transm Infect. 2009 Jun;85(3):199–200. doi: 10.1136/sti.2008.034165.

61 Johnson DC, Bhatta MP, Smith JS, Kempf MC, Broker TR, Vermund SH, et al. Assessment of high-risk human papillomavirus infections using clinician- And self-collected cervical sampling methods in rural women from far Western Nepal. PLoS One. 2014 Jun 30;9(6). doi: 10.1371/journal.pone.0101255.

62 Sharmin S, Sabikunnahar B, Aditya A, Khan MAAK, Nessa A, Ahsan CR, et al. Genotypic distribution and prevalence of human papillomavirus infection in an apparently healthy female population in Bangladesh. IJID Regions. 2021 Dec 1;1:130–4. doi: 10.1016/j.ijregi.2021.10.005.

63 Nahar Q, Sultana F, Alam A, Islam JY, Rahman M, Khatun F, et al. Genital human papillomavirus infection among women in Bangladesh: Findings from a population-based survey. PLoS One. 2014 Oct 1;9(10). doi: 10.1371/journal.pone.0107675.

64 Parwez A, Singh S, Kumar R, Kumari R, Kumar V, Prakash V, Ali M. Determination and evaluation of HR-HPV genotype in different communities of Bihar, India. PubMed Central (PMC). Published October 1, 2022. PMID: 36101850.

65 Sureshkumar BT, Shanmughapriya S, Das BC, Natarajaseenivasan K. A population-based study of the prevalence of HPV in three districts of Tamil Nadu, India. International Journal of Gynecology and Obstetrics. 2015 Apr 1;129(1):58–61. doi: 10.1016/j.ijgo.2014.10.025.

66 Peedicayil A, Thiyagarajan K, Gnanamony M, Pulimood SA, Jeyaseelan V, Kannangai R, Lionel J, Abraham OC, Abraham P. Prevalence and risk factors for human papillomavirus and cervical intraepithelial neoplasia among HIV-Positive women at a tertiary level hospital in India. J Low Genit Tract Dis. 2009;13(3):159-164. doi: 10.1097/LGT.0b013e31818fb40d.

67 Datta P, Bhatla N, Dar L, Patro AR, Gulati A, Kriplani A, et al. Prevalence of human papillomavirus infection among young women in North India. Cancer Epidemiol. 2010 Apr;34(2):157–61. doi: 10.1016/j.canep.2009.12.016.

68 Dutta S, Begum R, Mazumder (Indra) D, Mandal SS, Mondal R, Biswas J, et al. Prevalence of Human Papillomavirus in Women Without Cervical Cancer: A Population-based Study in Eastern India. International Journal of Gynecological Pathology. 2012;31(2):178–83. doi: 10.1097/PGP.0b013e3182399391.

69 Sherpa ATL, Clifford GM, Vaccarella S, Shrestha S, Nygard M, Karki BS, et al. Human papillomavirus infection in women with and without cervical cancer in Nepal. Cancer Causes and Control. 2010 Mar;21(3):323–30. doi: 10.1007/s10552-009-9467-z.

70 Thilagavathi A, Shanmughapriya S, Vinodhini K, Das BC, Natarajaseenivasan K. Prevalence of human papillomavirus (HPV) among college going girls using self collected urine samples from Tiruchirappalli, Tamilnadu. Arch Gynecol Obstet. 2012 Dec;286(6):1483–6. doi: 10.1007/s00404-012-2500-6.

71 Franceschi S, Rajkumar R, Snijders PJF, Arslan A, Mahé C, Plummer M, et al. Papillomavirus infection in rural women in southern India. Br J Cancer. 2005 Feb 14;92(3):601–6. doi: 10.1038/sj.bjc.6602348.

72 Mittal S, Mandal R, Banerjee D, Das P, Ghosh I, Panda C, et al. HPV detection-based cervical cancer screening program in low-resource setting: lessons learnt from a community-based demonstration project in India. Cancer Causes and Control. 2016 Mar 1;27(3):351–8. doi: 10.1007/s10552-015-0708-z.

73 Silver MI, Paul P, Sowjanya P, Ramakrishna G, Vedantham H, Kalpana B, et al. Shedding of epstein-barr virus and cytomegalovirus from the genital tract of women in a periurban community in Andhra Pradesh, India. J Clin Microbiol. 2011 Jul;49(7):2435–9. doi: 10.1128/JCM.02206-10.

74 Hussain S, Bharadwaj M, Nasare V, Kumari M, Sharma S, Hedau S, et al. Human papillomavirus infection among young adolescents in India: Impact of vaccination. J Med Virol. 2012 Feb;84(2):298–305. doi: 10.1002/jmv.22261.

75 Johnson DC, Lhaki P, Bhatta MP, Kempf MC, Smith JS, Bhattarai P, et al. Spousal migration and human papillomavirus infection among women in rural western Nepal. Int Health. 2016 Jul 1;8(4):261–8. doi: 10.1093/inthealth/ihw015.

76 Aziz H, Sattar AA, Mahmood H, Fatima S, Khurshid M, Faheem M. Prevalence of HPV types in HIV-positive and negative females with normal cervical cytology or dysplasia. J Clin Lab Anal. 2023 Apr 1;37(4). doi: 10.1002/jcla.24851.

77 Shahid M, Kazmi SU, Rehman A, Ainuddin J, Furqan S, Nazeer S. Cervical cancer screening and HPV genotype distribution among asymptomatic patients of Karachi Pakistan. Pak J Med Sci. 2015;31(3):493–8. doi: 10.12669/pjms.313.8004.

78 Baussano I, Tshering S, Choden T, Lazzarato F, Tenet V, Plummer M, et al. Cervical cancer screening in rural Bhutan with the care HPV test on self-collected samples: An ongoing cross-sectional, population-based study (REACH-Bhutan). BMJ Open. 2017 Jul 1;7(7). doi: 10.1136/bmjopen-2017-016309.

79 Becker ML, Ramesh B, Washington RG, Halli S, Blanchard JF, Moses S. Prevalence and determinants of HIV infection in South India: a heterogeneous, rural epidemic. AIDS. 2007;21(6):739-747. doi: 10.1097/QAD.0b013e328012b885.

80 Parvez R, Vijayachari P, Saha MK, Biswas L, Ramasamy J, Vins A, et al. Distribution of Human Papillomavirus Genotypes among the Women of South Andaman Island, India. Diagnostics. 2023 Sep 1;13(17). doi: 10.3390/diagnostics13172765.

81 Clifford GM, Baussano I, Heideman DAM, Tshering S, Choden T, Lazzarato F, et al. Human papillomavirus testing on self-collected samples to detect high-grade cervical lesions in rural Bhutan: The REACH-Bhutan study. Cancer Med. 2023 May 1;12(10):11828–37. doi: 10.1002/cam4.5851.

82 Shakya S, Thingulstad S, Syversen U, Nordbø SA, Madhup S, Vaidya K, et al. Prevalence of Sexually Transmitted Infections among Married Women in Rural Nepal. Infect Dis Obstet Gynecol. 2018;2018. doi: 10.1155/2018/4980396.

83 Todd CS, Nasir A, Mansoor GF, Sahibzada SM, Jagodzinski LL, Salimi F, Khateri MN, Hale BR, Barthel RV, Scott PT. Cross-sectional assessment of prevalence and correlates of blood-borne and sexually-transmitted infections among Afghan National Army recruits. BMC Infect Dis. 2012;12(1):196. doi: 10.1186/1471-2334-12-196.

84 Ramesh PS, Krishnamurthy S, Shrestha S, Nataraj SM, Devegowda D. Knowledge, awareness and prevalence of Human Papillomavirus among local University students and

Healthcare workers in South India: A cross-sectional study. Clin Epidemiol Glob Health. 2021 Oct 1;12:100864. doi: 10.1016/j.cegh.2021.100839.

85 Subramanian MJ, Rajaraman S, Vijayakumar V. A Community-Based Study on Prevalence, Genotype Distribution and Persistence of High-Risk Human Papilloma Virus Infection of Uterine Cervix in Rural South India. Indian J Gynecol Oncol. 2021 Mar 1;19(1). doi: 10.1007/s40944-021-00496-x.

86 Dakshinamurthy S, Racherla RG, Belagal P, Bharathi T, Sai Gopal DVR. Detection and Partial Molecular Characterization (E6–E7 Region-Early Genes) and Prevalence of Human Papillomavirus (HPV) Causing Cervical Cancer in and Around Tirupati Region, Andhra Pradesh. Indian J Gynecol Oncol. 2023 Jun 1;21(2). doi: 10.1007/s40944-022-00702-4.

87 Mishra R, Bisht D, Gupta M. Distribution and Prevalence of High-risk Human Papillomavirus Infection in Women of Western Uttar Pradesh, India: A Hospital-based Study. Journal of South Asian Federation of Obstetrics and Gynaecology. 2022 Mar 1;14(2):91–4. doi: 10.5005/jp-journals-10006-2013.

88 Shashidhar SC, Sonkusare S, Ramesh PS, Shetty AK, Shetty V, Devegowda D. Prevalence of High-Risk Human Papillomavirus in Women with Normal and Abnormal Pap Smear: A Cross Sectional Study from a Tertiary Hospital in South India. Indian J Gynecol Oncol. 2021 Dec 1;19(4). doi: 10.1007/s40944-021-00592-y.

89 Bhattacharya A, Sen S, Mandal P, Sharma Saha S, Sarkar S, Pathak OP, et al. Prevalence and age-wise distribution of Human Papillomavirus type 16/18 infections among hospital screened women of a peri-urban area in West Bengal: Impact of socio-demographic factors. Cancer Epidemiol. 2018 Jun 1;54:31–7. doi: 10.1016/j.canep.2018.03.005.

90 Asiaf A, Ahmad ST, Zargar MA, Mufti SM, Mir SH. Prevalence of human papillomavirus infection in a Kashmiri ethnic female population. Genet Test Mol Biomarkers. 2012 Aug 1;16(8):904–9. doi: 10.1089/gtmb.2011.0354.

91 Sauvaget C, Nene BM, Jayant K, Kelkar R, Malvi SG, Shastri SS, et al. Prevalence and determinants of high-risk human papillomavirus infection in middle-aged Indian women. Sex Transm Dis. 2011 Oct;38(10):902–6. doi: 10.1097/OLQ.0b013e318223be5f.

92 Sharma K, Kathait A, Jain A, Kujur K, Raghuwanshi S, Bharti AC, et al. Higher prevalence of human papillomavirus infection in adolescent and young adult girls belonging to different indian tribes with varied socio-Sexual lifestyle. PLoS One. 2015 May 1;10(5). doi: 10.1371/journal.pone.0125693.

93 Srivastava S, Gupta S, Roy JK. High prevalence of oncogenic HPV-16 in cervical smears of asymptomatic women of eastern Uttar Pradesh, India: A population-based study. J Biosci. 2012 Mar;37(1):63–72. doi: 10.1007/s12038-012-9181-y.

94 Mapitigama N, Moonesinghe LN, Punchihewa R, Perera C. A Descriptive Study of Different Methods of Cervical Cancer Screening among Ever-Married Women in 35-Year and 45-Year Cohorts in Kalutara District, Sri Lanka. Asian Pacific Journal of Cancer Prevention. 2023;24(5):1487–93. doi: 10.31557/APJCP.2023.24.5.1487.

95 Vinodhini K, Shanmughapriya S, Sanmugham S, Senthikumar G, Das BC, Natarajaseenivasan K. Prevalence of high-risk HPV and associated risk factors in cases of cervical carcinoma in Tamil Nadu, India. International Journal of Gynecology and Obstetrics. 2012;119(3):253–6. doi: 10.1016/j.ijgo.2012.06.019.

96 Khanna RK, Rao SK, Tiwary A, Rai S, Khanna AK, Khanna RG, et al. Detection of human papilloma virus 16 and 18 DNA sequences by Southern blot hybridization in oral leukoplakia and squamous cell carcinoma. Indian J Surg. 2009;71. doi: 10.1007/s12262-009-0019-2.

97 Naushad W, Surriya O, Sadia H. Prevalence of EBV, HPV and MMTV in Pakistani breast cancer patients: A possible etiological role of viruses in breast cancer. Infection, Genetics and Evolution. 2017 Oct 1;54:230–7. doi: 10.1016/j.meegid.2017.07.010.

98 Gunasekera SK, Perera KA, Fernando C, Udagama PV. A shifting paradigm in the aetiology of oral and pharyngeal cancer in Sri Lanka: A case-control study providing serologic evidence for the role of oncogenic HPV types 16 and 18. Infect Agent Cancer. 2015 Apr 1;10(1). doi: 10.1186/s13027-015-0007-z.

99 Saranath D, Khan Z, Tandle AT, Dedhia P, Sharma B, Contractor R, et al. HPV16/18 prevalence in cervical lesions/cancers and p53 genotypes in cervical cancer patients from India. Gynecol Oncol. 2002;86(2):157–62. doi: 10.1006/gyno.2002.6735.

100 Gopalkrishna V, Aggarwal N, Malhotra VL, Koranne R V., Mohan VP, Mittal VP, et al. Chlamydia trachomatis and human papillomavirus infection in Indian women with sexually transmitted diseases and cervical precancerous and cancerous lesions. Clinical Microbiology and Infection. 2000;6(2):88–93. doi: 10.1046/j.1469-0691.2000.00024.x.

101 Pandit L, Malli C, D’Cunha A, Shetty R, Singhal B. Association of Epstein-Barr virus infection with multiple sclerosis in India. J Neurol Sci. 2013 Feb 15;325(1–2):86–9. doi: 10.1016/j.jns.2012.12.004.

102 Lourembam DS, Singh AR, Sharma TD, Singh TS, Singh TR, Singh LS. Evaluation of risk factors for nasopharyngeal carcinoma in a high-risk area of India, the Northeastern region.

Asian Pacific Journal of Cancer Prevention. 2015;16(12):4927–35. doi: 10.7314/apjcp.2015.16.12.4927.

103 Janani MK, Malathi J, Rela M, Farouk M, Padmapriya J, Madhavan HN. Genotypic detection of Epstein-Barr Virus in pediatric transplant recipients from India. Indian Pediatr. 2015;52(11):946-950. doi: 10.1007/s13312-015-0750-7.

104 Janani MK, Malathi J, Appaswamy A, Singha NR, Madhavan HN. A hospital based pilot study on Epstein-Barr virus in suspected infectious mononucleosis pediatric patients in India. J Infect Dev Ctries. 2015 Oct 1;9(10):1133–8. doi: 10.3855/jidc.6199.

105 Sinha M, Rao CR, Shafiulla M, Shankaranand B, Viveka BK, Lakshmaiah KC, et al. Plasma Epstein Barr viral load in adult-onset Hodgkin lymphoma in South India. Hematology/ Oncology and Stem Cell Therapy. 2016 Mar 1;9(1):8–13. doi: 10.1016/j.hemonc.2015.11.004.

106 Ghosh SK, Singh AS, Mondal R, Kapfo W, Khamo V, Singh YI. Dysfunction of mitochondria due to environmental carcinogens in nasopharyngeal carcinoma in the ethnic group of Northeast Indian population. Tumor Biology. 2014;35(7):6715–24. doi: 10.1007/s13277-014-1897-x.

107 Noorali S, Pervez S, Yaqoob N, Moatter T, Nasir MI, Haroon S, et al. Prevalence and characterization of anaplastic large cell lymphoma and its association with Epstein-Barr virus in Pakistani patients. Pathol Res Pract. 2004 Dec 15;200(10):669–79. doi: 10.1016/j.prp.2004.08.004.

108 Borthakur P, Kataki K, Keppen C, Khamo, Medhi S, Deka M. Expression of Epstein Barr Virus Encoded EBNA1 and LMP1 Oncoproteins in Nasopharyngeal Carcinomas from

Northeast India. PubMed [Internet]. 2016 Jan 1;17(7):3411–6. Available from: https://pubmed.ncbi.nlm.nih.gov/27509984. PMID: 27509984.

109 Chatterjee K, Roy SD, Chakraborty K, Haque A, Chakrabarti S, Mukherjee S, et al. Lifestyle, Epstein-Barr virus infection, and other factors could impede nasopharyngeal cancer survivorship: a five-year cross-sectional study in North Eastern India. Virusdisease. 2022 Dec 1;33(4):371–82. doi: 10.1007/s13337-022-00789-5.

110 Sangam K, Kumar Y, Minz RW, Varma N, Varma S, Anand S. Patients with Plasma Cell Disorders Have High EBV DNA in Peripheral Blood than the General Population. Pathology and Oncology Research. 2020 Oct 1;26(4):2789–94. doi: 10.1007/s12253-019-00640-1.

111 Sachithanandham J, Kannangai R, Pulimood SA, Desai A, Abraham AM, Abraham OC, et al. Significance of Epstein-Barr virus (HHV-4) and CMV (HHV-5) infection among subtype-C human immunodeficiency virus-infected individuals. Indian J Med Microbiol. 2014;32(3):261–9. doi: 10.4103/0255-0857.136558.

112 Reddy SS, Sharma S, Mysorekar V. Expression of Epstein–Barr virus among oral potentially malignant disorders and oral squamous cell carcinomas in the South Indian tobacco-chewing population. Journal of Oral Pathology and Medicine. 2017 Jul 1;46(6):454–9. doi: 10.1111/jop.12508.

113 Sharma U, Singhal P, Bandil K, Patle R, kumar A, Neyaz K, et al. Genetic variations of TLRs and their association with HPV/EBV, co-infection along with nicotine exposure in the development of premalignant/malignant lesions of the oral cavity in Indian population. Cancer Epidemiol. 2019 Aug 1;61:38–49. doi: 10.1016/j.canep.2019.05.003.

114 Rizvi M, Azam M, Shukla I, Malik A, Ajmal MR. Leptospirosis: An emerging disease in cryptogenic hepatitis patients in north India. Sultan Qaboos Univ House Expert. 2011.

Available from: https://squ.elsevierpure.com/en/publications/leptospirosis-an-emerging-disease-in-cryptogenic-hepatitis-patien

115 Husseini AA, Fidan I, Saeed KMI, Bozdayı AM. Seroprevalence of Cytomegaloviruses in Afghanistan. Journal of Microbiology and Infectious Diseases. 2019 Jun 15;78–82. doi: 10.5799/jmid.574566.

116 Chakravarti A, Tewari S, Bhalla P. Human cytomegalovirus infection among patients living with AIDS in a tertiary level hospital in India. J Int Assoc Physicians AIDS Care. 2010 Mar;9(2):94–7. doi: 10.1177/1545109710366180.

117 Das B, Kaur G, Basu S. Seroprevalence of cytomegalovirus antibodies among blood donors and Multitransfused recipients - A study from north India. Transfusion and Apheresis Science. 2014;50(3):438–42. doi: 10.1016/j.transci.2014.02.022.

118 Chaudhari CN, Bindra MS. Seroprevalence of cytomegalovirus among voluntary blood donors. Med J Armed Forces India. 2009;65(3):252–4. doi: 10.1016/S0377-1237(09)80016-6.

119 Surpam RB, Kamlakar UP, Khadse R, Qazi M, Jalgaonkar SV, Jalgaonkar SV. Serological study for TORCH infections in women with bad obstetric history. J Obstet Gynecol India. 2006;56:60-3.

120 Tewari R, Nijhawan VS, Mishra MN, Dudeja P, Salopal TK. Prevalence of helicobacter pylori, cytomegalovirus, and chlamydia pneumonia immunoglobulin seropositivity in coronary artery disease patients and normal individuals in north indian population. Med J Armed Forces India. 2012;68(1):53–7. doi: 10.1016/S0377-1237(11)60121-4.121 Dubey S, Rodrigues C, Nikam C, Samant R. Cytomegalovirus in Indian systemic lupus

erythematosus patients: trouble maker or onlooker? Pan Afr Med J. 2020;37. doi:10.11604/pamj.2020.37.38.18836. doi: 10.11604/pamj.2020.37.38.18836.

122 Anuradha, B, Pratibha M M, Vijayadurga S. The reactivation of the cytomegalovirus (CMV) infection in HIV infected patients. Journal of Clinical and Diagnostic Research [Internet]. 2011 Aug;5(4):749–51. Available from: https://jcdr.net/article_abstract.asp?issn=0973709x&year=2011&volume=5&issue=4&page=749&issn=0973-709x&id=1413

123 Mujtaba S, Mujtaba S, Varma S, Sehgal S. Article in The Indian Journal of Medical Research. 2003 Mar [Internet]. Vol. 117. Available from: https://www.researchgate.net/publication/9041490. doi: 10.4236/wja.2014.41007.

124 Kothari J. Seroprevalence of Cytomegalovirus among Voluntary Blood Donors in Delhi, India. J Health Popul Nutr [Internet]. 2002;20(4):348–51. Available from: https://www.researchgate.net/publication/10836990. PMID: 12659416.

125 Kumar H, Gupta PK, Kumar S, Sarkar RS. Is seroprevalence of anti-IGM CMV among blood donors relevant in India? Indian J Pathol Microbiol. 2008 Jul-Sep;51(3). Available from: http://www.ijpmonline.org

126 Sharma A, Rasul E, Hazarika N. A serological study of cytomegalovirus infection in pregnant and non-pregnant women at Gauhati Medical College and Hospital. J Indian Med Assoc. 2007;105(6):320, 322-3. PubMed. 2007 Jun 1. Available from: https://pubmed.ncbi.nlm.nih.gov/18232177/. PMID: 18232177.

127 Padmavati S, Gupta U, Agarwal H. Chronic infections & coronary artery disease with special reference to Chlamydia pneumoniae. PubMed Central (PMC). 2012 Feb 1. Available from: https://www.ncbi.nlm.nih.gov/pmc/articles/PMC3336855/. PMID: 22446866.

128 Thapa N, Maharjan M, Shrestha G, Maharjan N, Petrini MA, Zuo N, et al. Prevalence and type-specific distribution of human papillomavirus infection among women in mid-western rural, Nepal- A population-based study. BMC Infect Dis. 2018 Jul 20;18(1). doi: 10.1186/s12879-018-3175-9.

129 Perera KCM, Mapitigama N, Abeysena H. The feasibility of new HPV/DNA test as a primary cervical cancer screening method among 35- years- old ever-married women in Kalutara district; a cross-sectional study. BMC Public Health. 2021 Dec 1;21(1). doi: 10.1186/s12889-021-10190-4.

130 Gibney L. STD in Bangladesh's trucking industry: prevalence and risk factors. Sex Transm Infect. 2002;78(1):31-36. doi: 10.1136/sti.78.1.31.

131 Ibrahim S, Siddiqui AA, Siddiqui AR, Ahmed W, Moss PAH, Lalani ENMA. Sociodemographic factors associated with IgG and IgM seroprevalence for human cytomegalovirus infection in adult populations of Pakistan: A seroprevalence survey. Vol. 16, BMC Public Health. BioMed Central; 2016. doi:10.1186/s12889-016-3772-8.

132 Hawkes S, Morison L, Chakraborty J, Gausia K, Ahmed F, Islam SS, et al. Reproductive tract infections: prevalence and risk factors in rural Bangladesh. Bull World Health Organ. 2002;80(3):180-7. PMID: 11984603.

133 Munir A, Khan S, Khan S, Attaullah S, Munir M, Saleem A, et al. Frequency and association of Epstein-Barr Virus genotype in rheumatoid arthritis patients of Khyber Pakhtunkhwa, Pakistan. PLoS One. 2023 Dec 1;18(12 December). doi: 10.1371/journal.pone.0295124.

134 Dworzański J, Drop B, Kliszczewska E, Strycharz-Dudziak M, Polz-Dacewicz M. Prevalence of Epstein-Barr virus, human papillomavirus, cytomegalovirus and herpes simplex

virus type 1 in patients with diabetes mellitus type 2 in south-eastern Poland. PLoS One. 2019 Sep;14(9). doi: 10.1371/journal.pone.0222607.

135 Zuhair, M., Smit, G. S. A., Wallis, G., Jabbar, F., Smith, C., Devleesschauwer, B., & Griffiths, P. (2019). Estimation of the worldwide seroprevalence of cytomegalovirus: A systematic review and meta-analysis. Reviews in Medical Virology, 29(3). doi: 10.1002/rmv.2034.

136 Wen, L., Qiu, Y., Cheng, S., Jiang, X., Ma, Y., Fang, W., Wang, W., Cui, J., Ruan, Q., Zhao, F., Hu, F., & Luo, M. (2017). Serologic and viral genome prevalence of HSV, EBV, and HCMV among healthy adults in Wuhan, China. Journal of Medical Virology, 90(3), 571–581. doi: 10.1002/jmv.24989.

137 De Sanjosé, S., Diaz, M., Castellsagué, X., Clifford, G., Bruni, L., Muñoz, N., & Bosch, F. X. (2007). Worldwide prevalence and genotype distribution of cervical human papillomavirus DNA in women with normal cytology: a meta-analysis. Lancet Infectious Diseases, 7(7), 453–459. doi: 10.1016/s1473-3099(07)70158-5.

138 Cunningham AL, Taylor R, Taylor J, Marks C, Shaw J, Mindel A. Prevalence of infection with herpes simplex virus types 1 and 2 in Australia: a nationwide population based survey. Sex Transm Infect. 2006;82(2):164-168. doi: 10.1136/sti.2005.016899.

139 Lechuga J, Swain GR, Weinhardt LS. The cross-cultural variation of predictors of human papillomavirus vaccination intentions. J Womens Health. 2011;20(2):225-30. doi: 10.1089/jwh.2010.1993.

140 Goel K, Vasudevan L. Disparities in healthcare access and utilization and human papillomavirus (HPV) vaccine initiation in the United States. Hum Vaccin Immunother. 2021;17(12):5390-6. doi: 10.1080/21645515.2021.1989919.

141 HPV Centre. Global HPV Vaccination Coverage. [PDF]. Available from: https://hpvcentre.net/statistics/reports/XSX.pdf?t=1719851770434.

142 Fatahzadeh M, Schwartz RA. Human herpes simplex virus infections: Epidemiology, pathogenesis, symptomatology, diagnosis, and management. J Am Acad Dermatol. 2007;57(5):737-63. doi: 10.1016/j.jaad.2007.06.027.

143 Allsworth JE, Lewis VA, Peipert JF. Viral sexually transmitted infections and bacterial vaginosis: 2001–2004 National Health and Nutrition Examination Survey data. Sex Transm Dis. 2008;35(9):791-6. doi: 10.1097/olq.0b013e3181788301.

144 Ramaswamy M. The epidemiology of genital infection with herpes simplex virus types 1 and 2 in genitourinary medicine attendees in inner London. Sex Transm Infect. 2005;81(4):306-8. doi: 10.1136/sti.2004.011643.

145 Beydoun HA, Dail J, Ugwu B, Boueiz A, Beydoun MA. Socio-demographic and behavioral correlates of herpes simplex virus type 1 and 2 infections and co-infections among adults in the USA. Int J Infect Dis. 2010;14. doi: 10.1016/j.ijid.2009.12.007.

146 Johnson J, Anderson B, Pass RF. Prevention of maternal and congenital cytomegalovirus infection. Clin Obstet Gynecol. 2012 Jun;55(2):521–30. doi: 10.1097/GRF.0b013e3182510b7b.

147 Plotkin SA, Boppana SB. Vaccination against the human cytomegalovirus. Vaccine. 2019 Nov 28;37(50):7437–42. doi: 10.1016/j.vaccine.2018.02.089.

148 Smets F, Sokal EM. Prevention and treatment for epstein-barr virus infection and related cancers. Recent Results in Cancer Research. 2014;193:173–90. doi: 10.1007/978-3-642-38965-8_10.

149 Vorsters A, Bosch FX, Poljak M, Waheed D e. N, Stanley M, Garland SM. HPV prevention and control – The way forward. Vol. 156, Preventive Medicine. Academic Press Inc.; 2022. doi: 10.1016/j.ypmed.2022.106960.

150 Martín JM, Villalón G, Jordá E. Update on the treatment of genital herpes. Actas Dermosifiliogr. 2009;100(1):22–32. doi: 10.1016/s1578-2190(09)70006-x.

151 Gentile G, Micozzi A. Speculations on the clinical significance of asymptomatic viral infections. Clin Microbiol Infect. 2016;22(7):585-588. doi: 10.1016/j.cmi.2016.07.016

152 Pinninti S, Hough-Telford C, Pati S, Boppana S. Cytomegalovirus and Epstein-Barr virus infections. Pediatr Rev. 2016;37(6):223-234. doi: 10.1542/pir.2015-0072

153 World Health Organization: WHO. Human papillomavirus and cancer. 2024 Mar 5. Available from: https://www.who.int/news-room/fact-sheets/detail/human-papilloma-virus-and-cancer

154 World Health Organization: WHO. Herpes simplex virus. 2023 Apr 5. Available from: https://www.who.int/news-room/fact-sheets/detail/herpes-simplex-virus

155 Looker KJ, Magaret AS, Turner KME, Vickerman P, Gottlieb SL, Newman LM. Global estimates of prevalent and incident herpes simplex virus Type 2 infections in 2012. PLoS One. 2015;10(1). doi: 10.1371/journal.pone.0114989.

156 GBS (Guillain-Barré syndrome) and vaccines. Vaccine Safety. CDC. Available from: https://www.cdc.gov/vaccinesafety/concerns/guillain-barre-syndrome.html

157 World Health Organization: WHO. Cervical cancer. 2024 Mar 5. Available from: https://www.who.int/news-room/fact-sheets/detail/cervical-cancer

158 James SH, Sheffield JS, Kimberlin DW. Mother-to-child transmission of herpes simplex virus. J Pediatr Infect Dis Soc. 2014;3(Suppl 1). doi: 10.1093/jpids/piu050.

Fig 2. The forest plot represents the overall pooled prevalence of CMV, EBV, HPV, and CMV among South Asian healthy

Click here to access/download;Figure;Fig 2.jpg

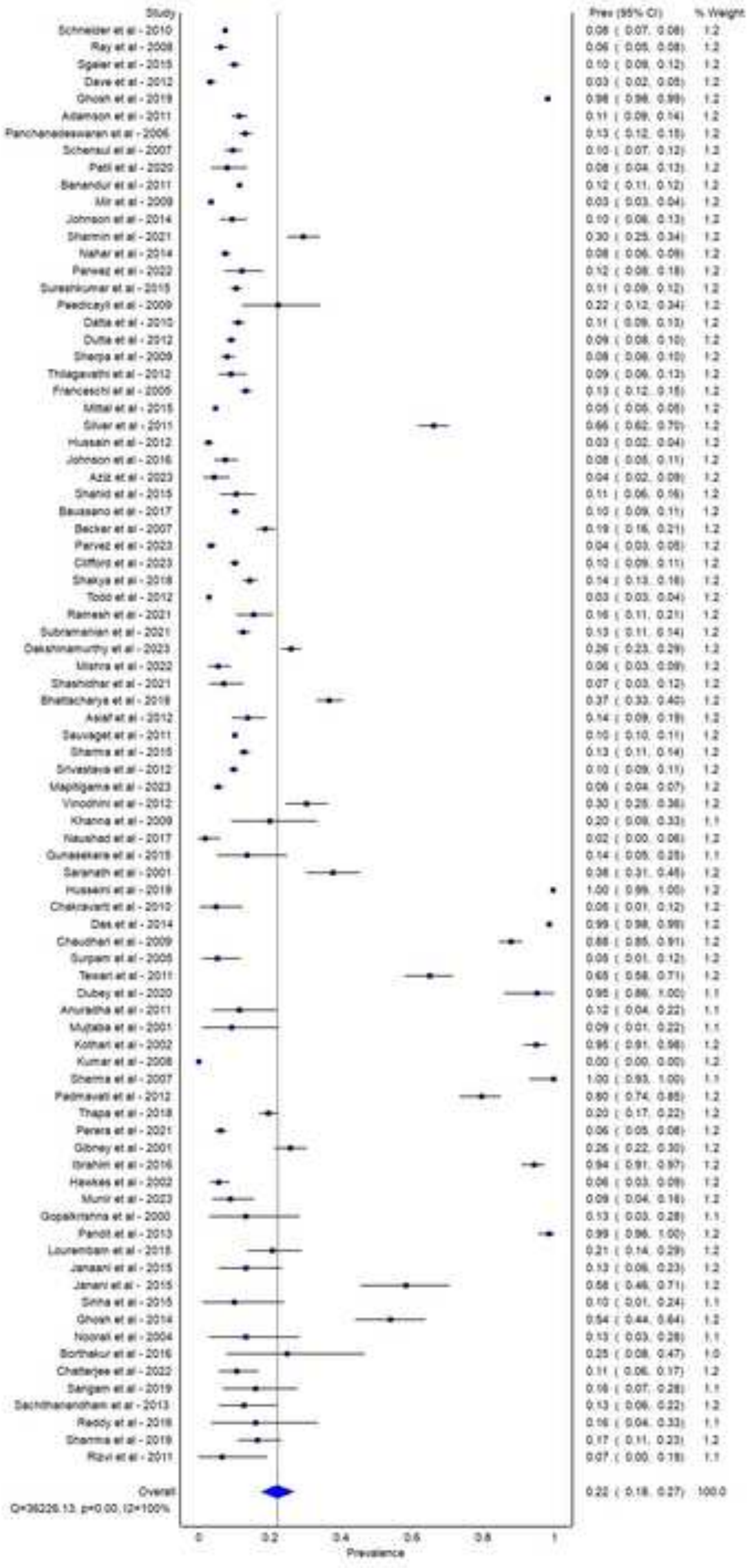

Fig 3. The Forest plot shows the prevalence of CMV infection among the South Asian healthy population. Each horizontal line represents a separate study, with the box

Click here to access/download;Figure;Fig 3.jpg

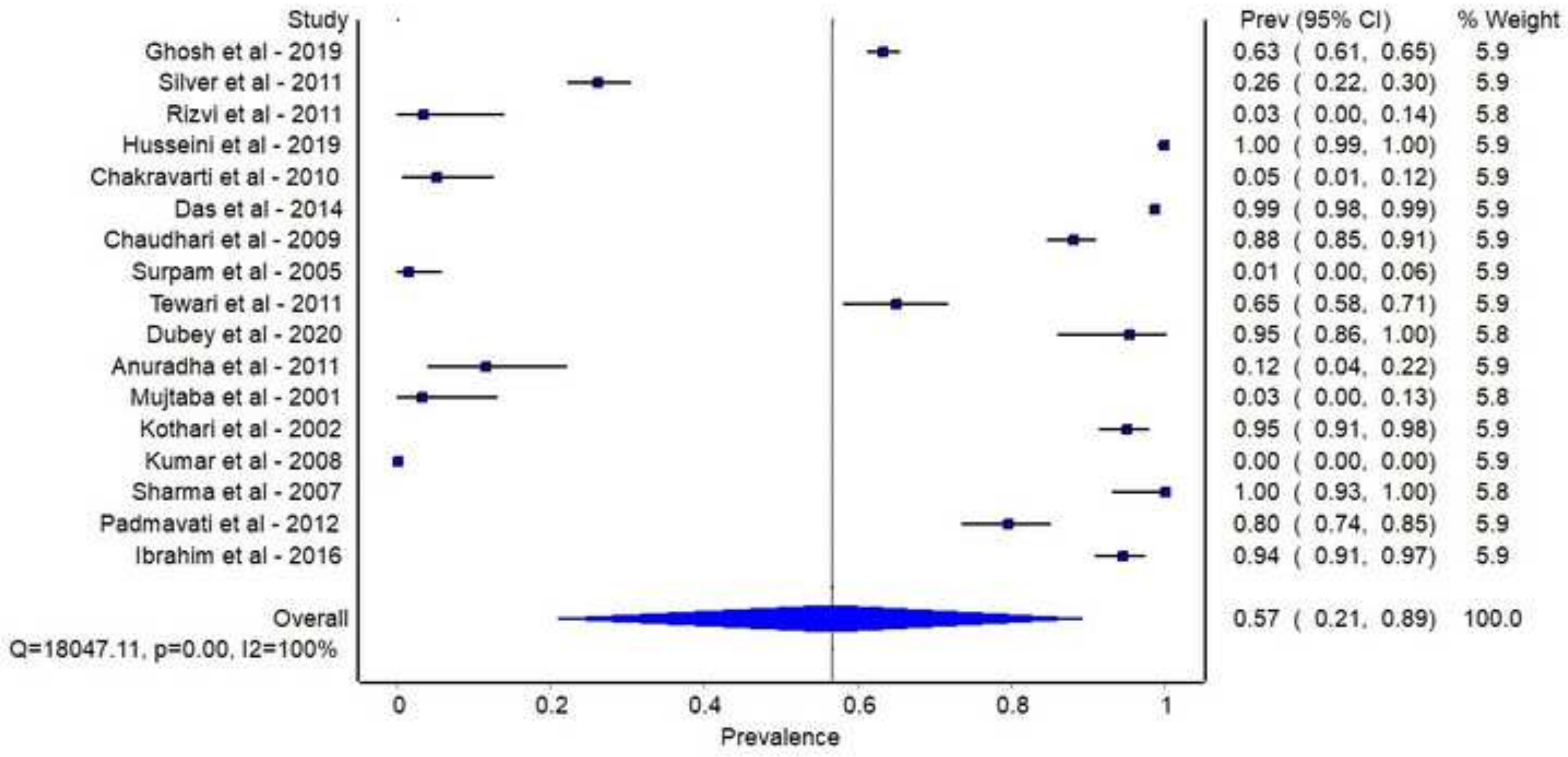

Fig 4. The Forest plot shows the prevalence of EBV infection among the South Asian healthy population. Each horizontal line represents a separate study, with the box

Click here to access/download;Figure;Fig 4.jpg

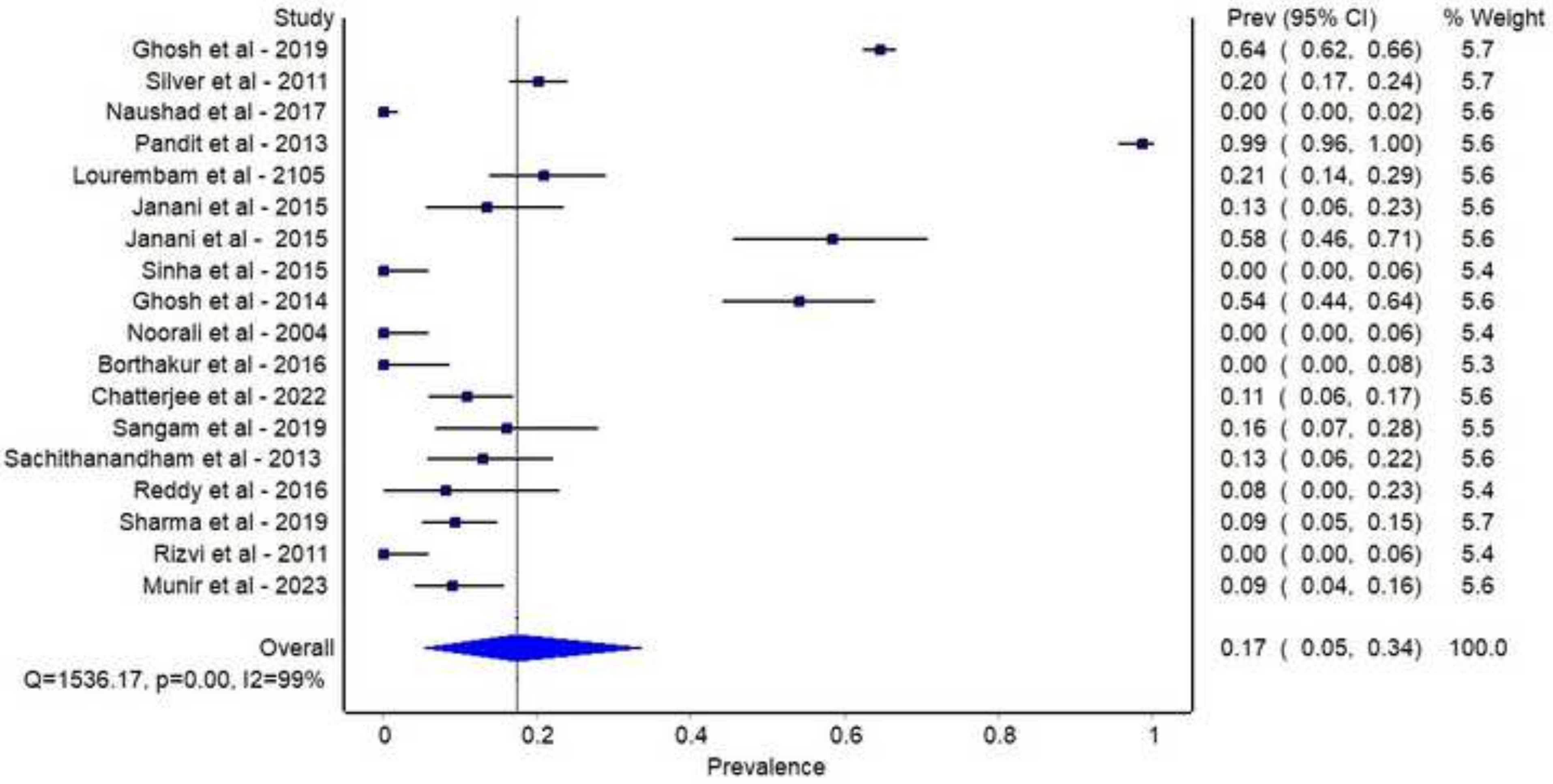

Fig 5. The Forest plot shows the prevalence of HPV infection among the South Asian healthy population. Each horizontal line represents a separate study, with the box

Click here to access/download;Figure;Fig 5.jpg

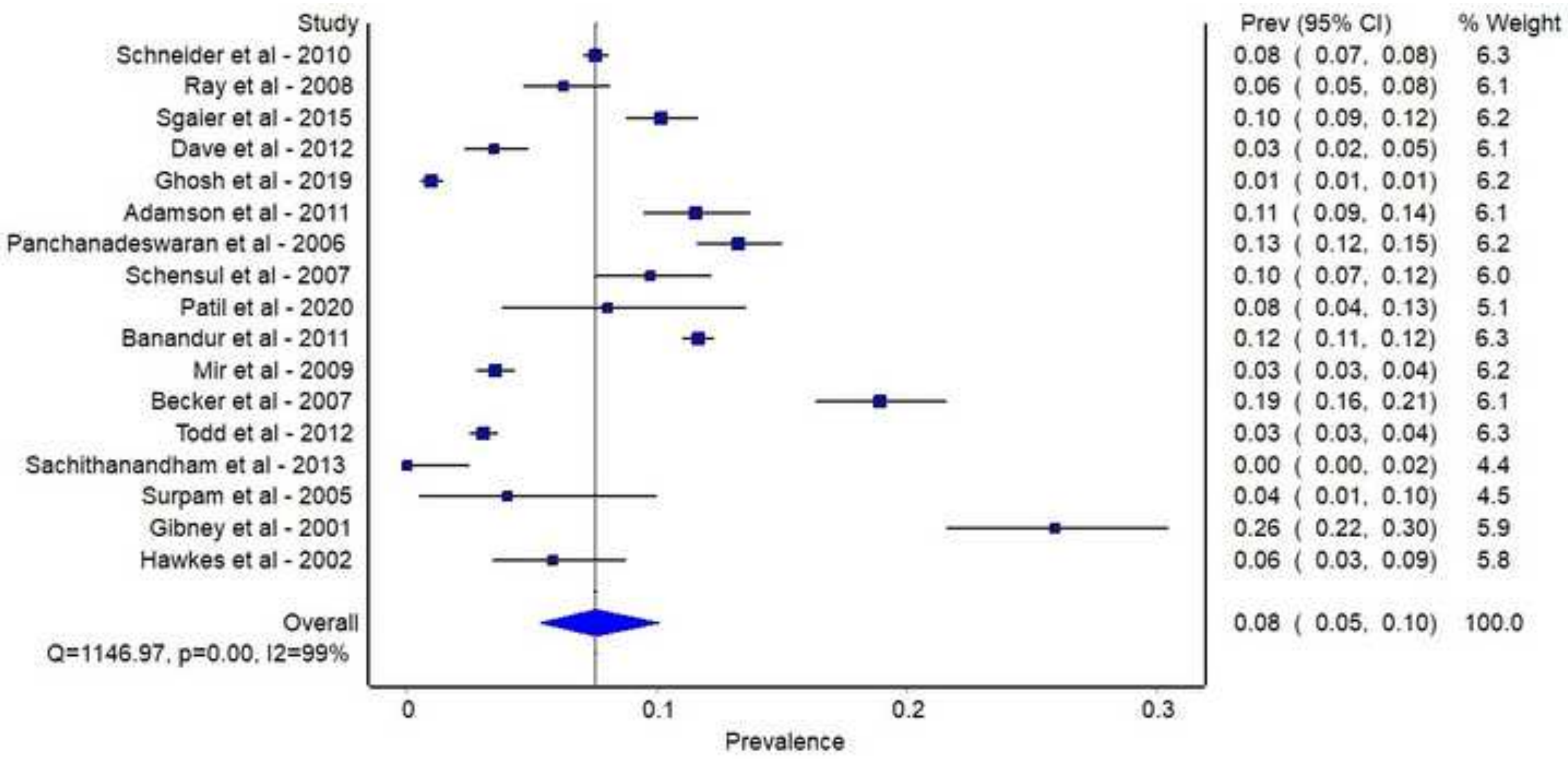

Fig 6. The Forest plot shows the prevalence of HSV infection among the South Asian healthy population. Each horizontal line

Click here to access/download;Figure;Fig 6.jpg

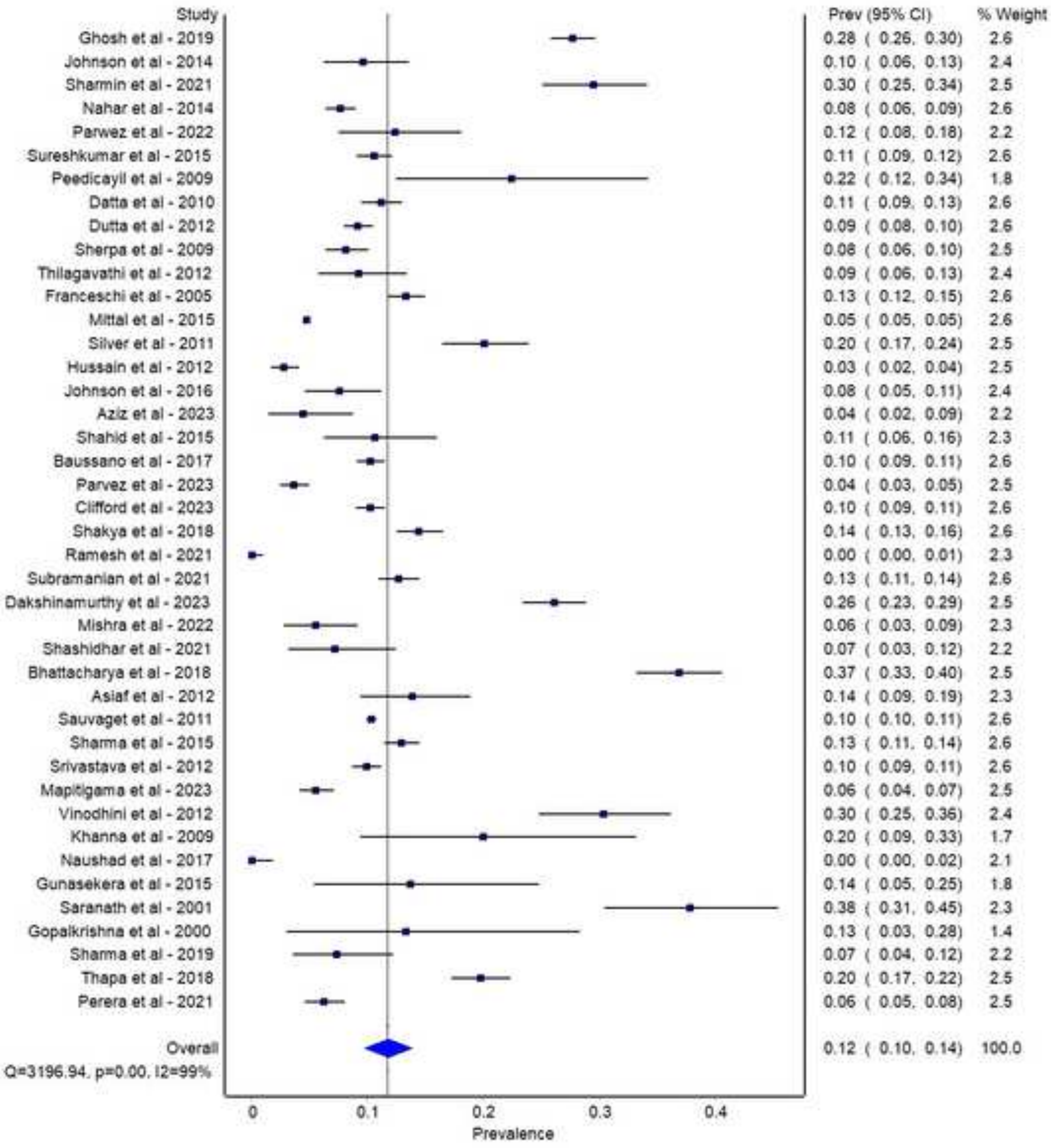

S1 Fig. The Forest plot shows the prevalence of CMV infection in India among the healthy population. Each horizontal line represents a separate study, with the box

Click here to access/download;Figure;S1 Fig.jpg

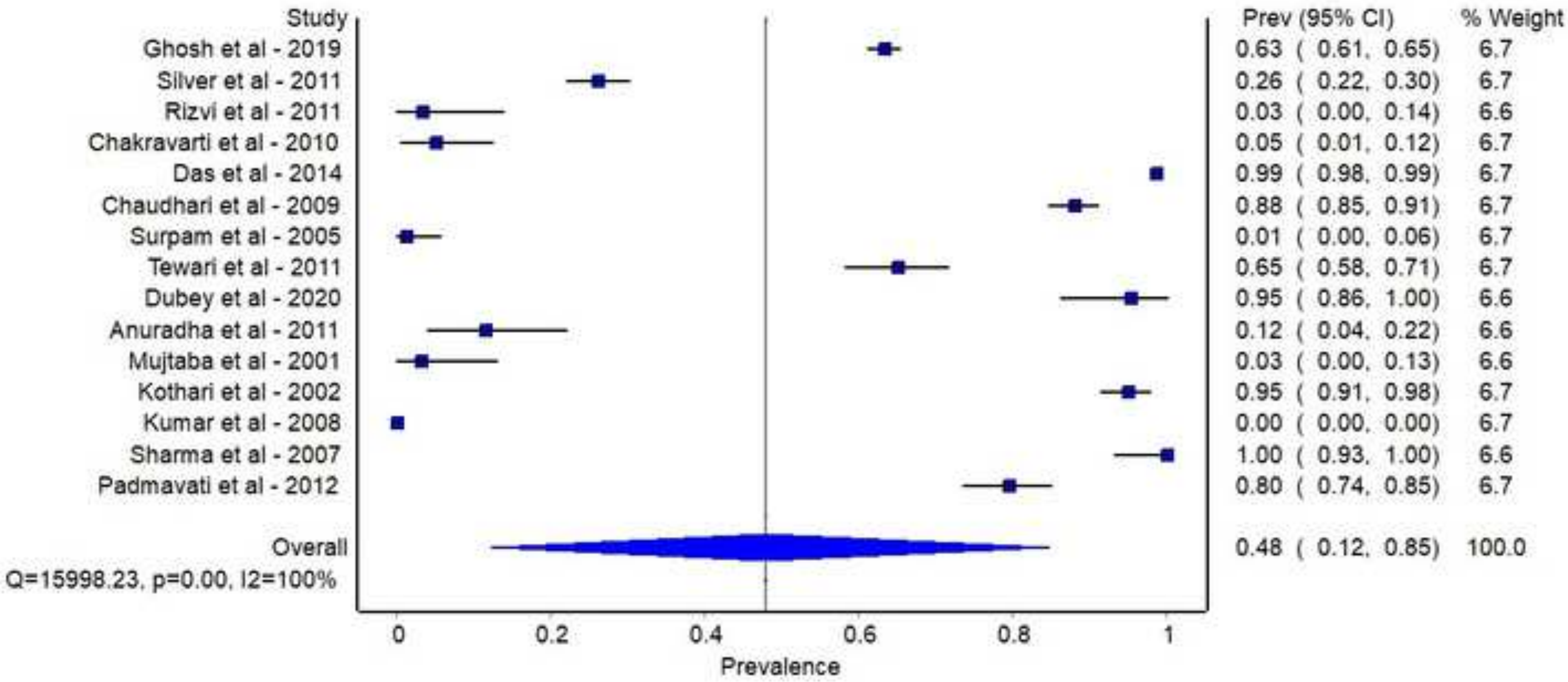

S2 Fig. The Forest plot shows the prevalence of EBV infection in India among the healthy population. Each horizontal line represents a separate study, with the box

Click here to access/download;Figure;S2 Fig.jpg

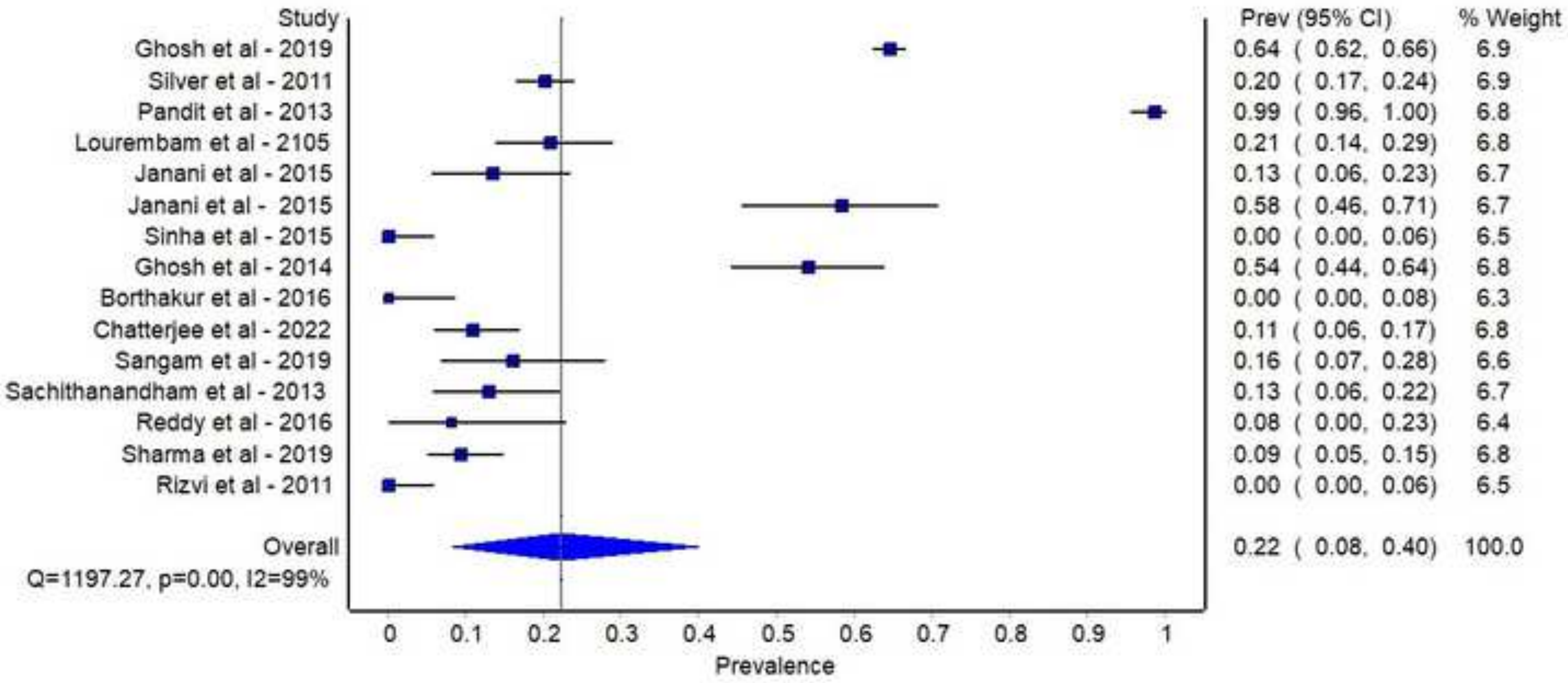

S3 Fig. The Forest plot shows the prevalence of HPV infection in India among the healthy population. Each horizontal line represents a separate study, with the box

Click here to access/download;Figure;S3 Fig.jpg

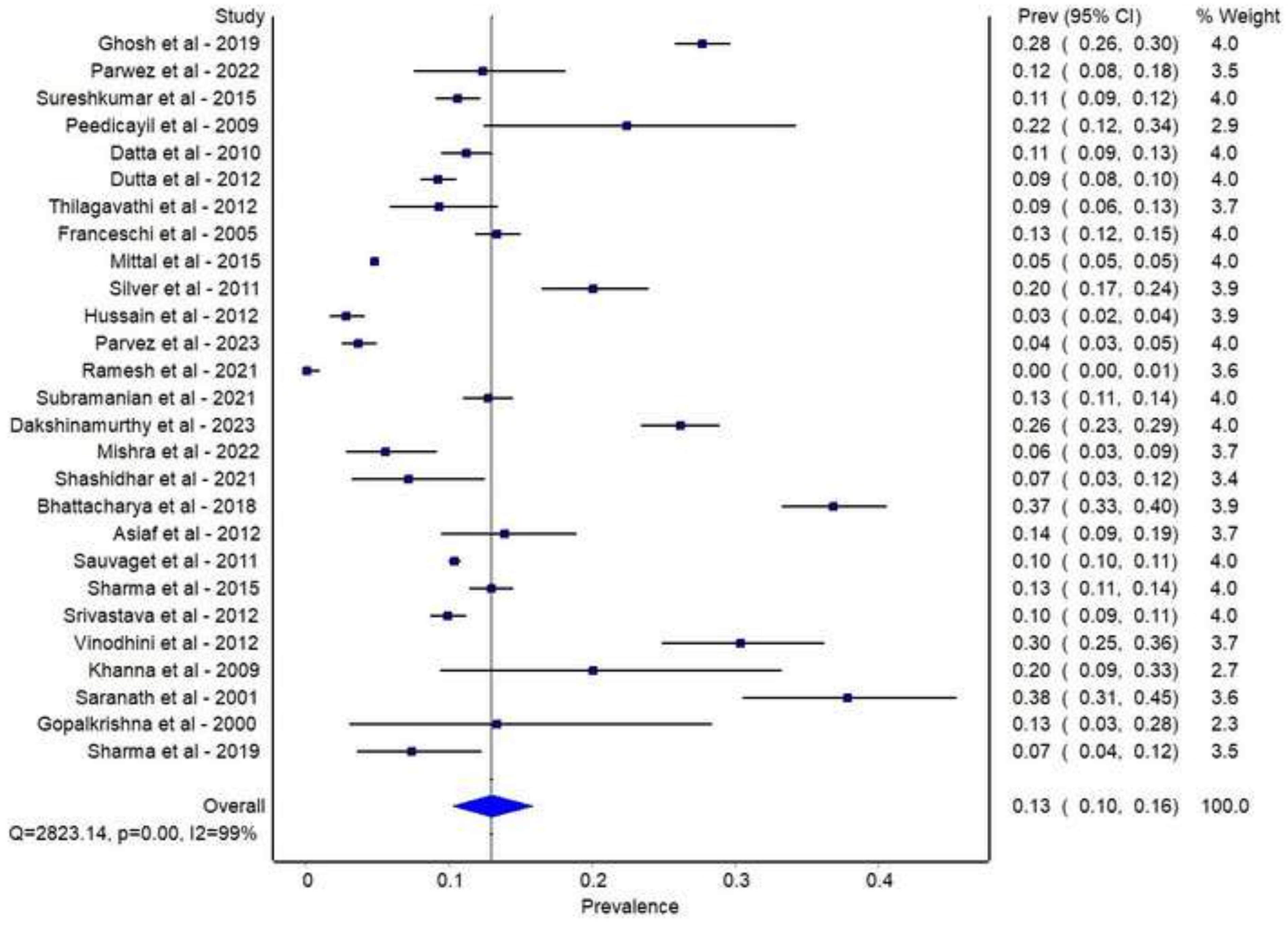

S4 Fig. The Forest plot shows the prevalence of HPV infection in the female population. Each horizontal line represents a separate study, with the box indicating

Click here to access/download;Figure;S4 Fig.jpg

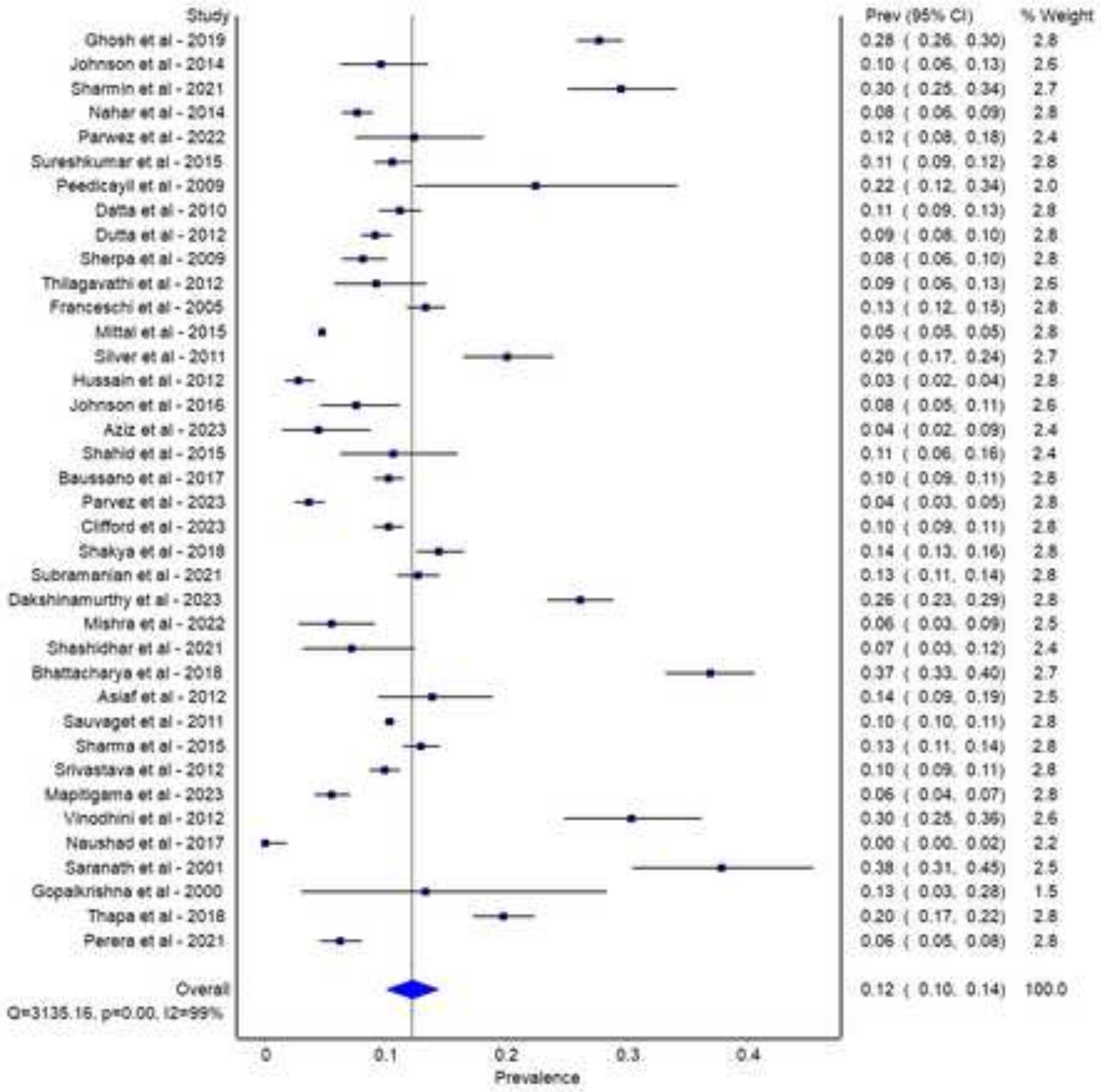

S5 Fig. The Forest plot shows the prevalence of HSV infection in India among the healthy population. Each horizontal line represents a separate study, with the box

Click here to access/download;Figure;S5 Fig.jpg

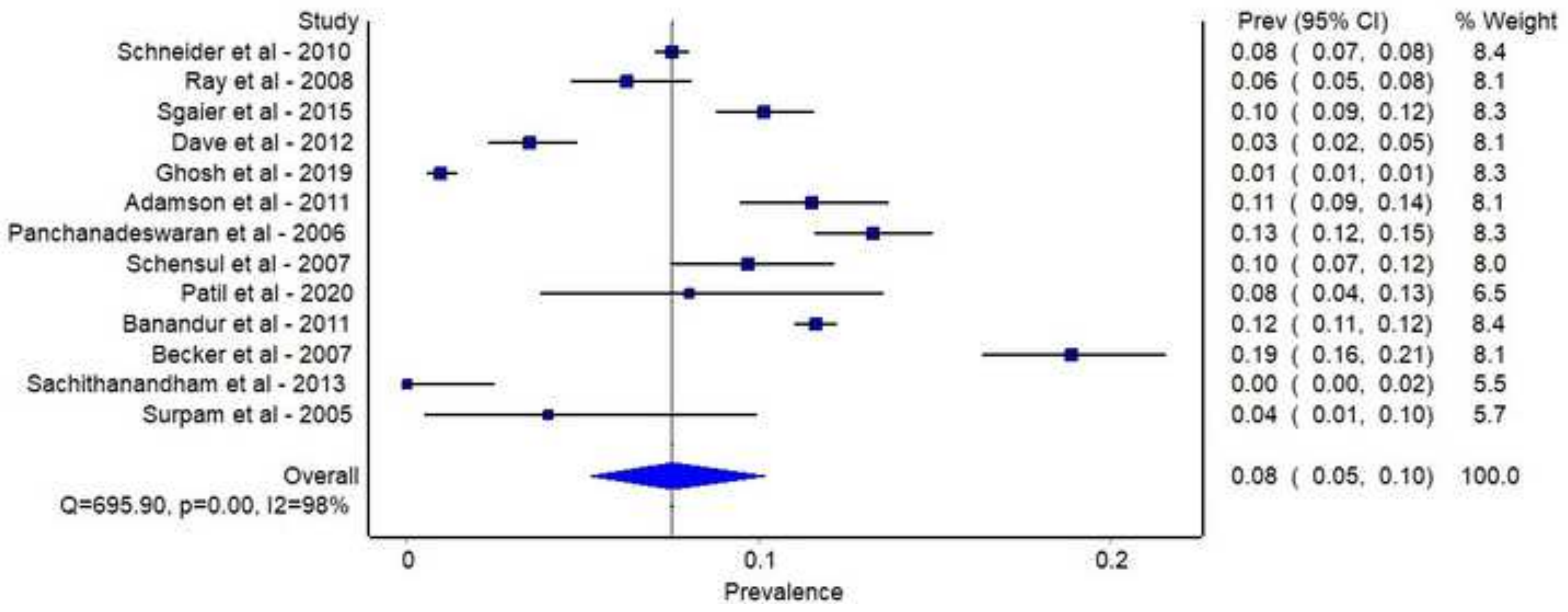

S6 Fig. The Forest plot shows the prevalence of HSV infection in the male population.
Each horizontal line represents a separate study, with the box indicating the

Click here to access/download;Figure;S6 Fig.jpg

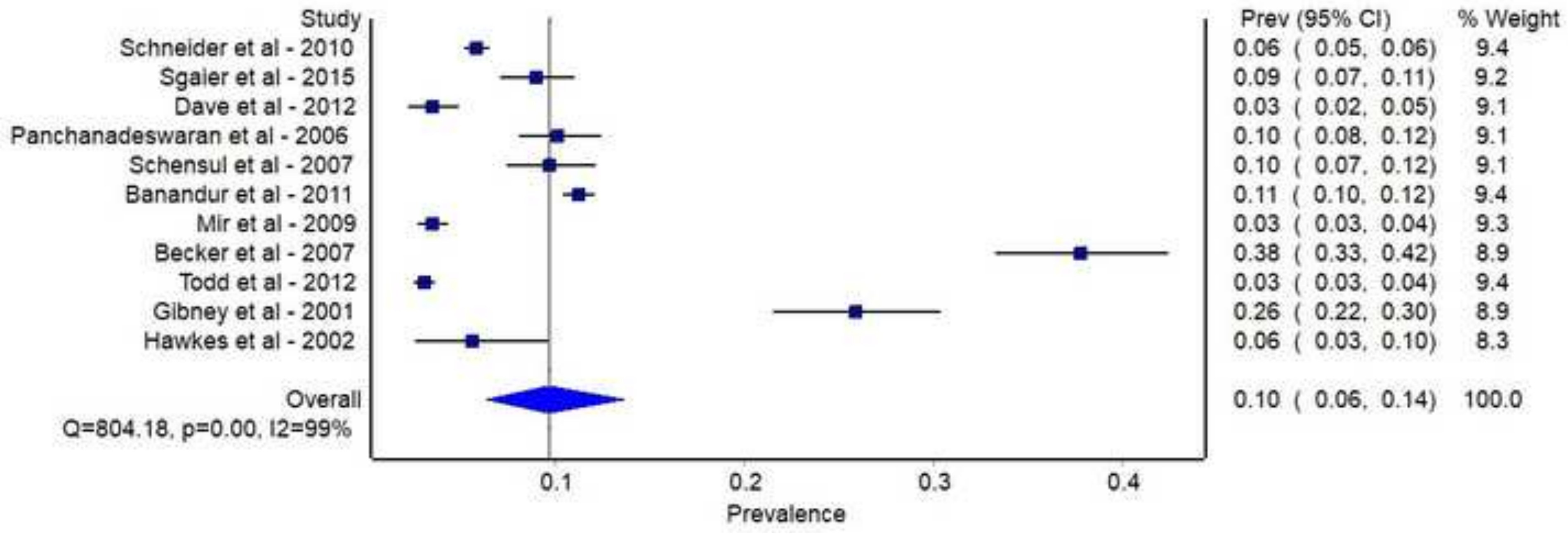

S7 Fig. The Forest plot shows the prevalence of HSV infection in the female population. Each horizontal line represents a separate study, with the box indicating

Click here to access/download;Figure;S7 Fig.jpg

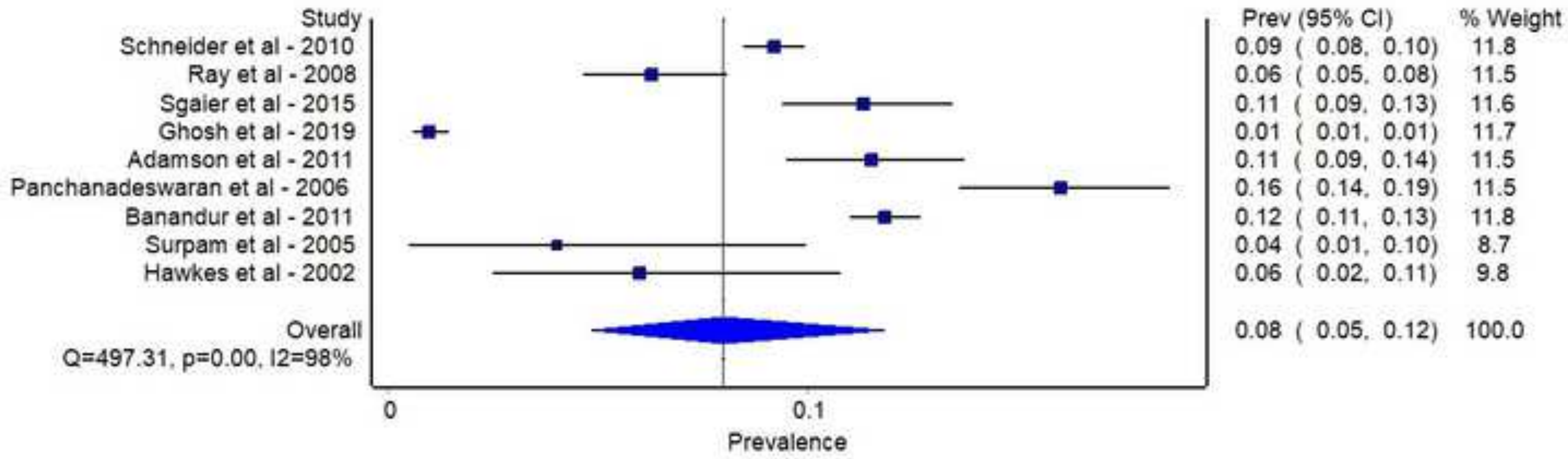

Fig 1. PRISMA flowchart outlining the methodology for literature search and the inclusion/exclusion criteria applied in this study

Click here to access/download;Figure;Fig 1.jpg

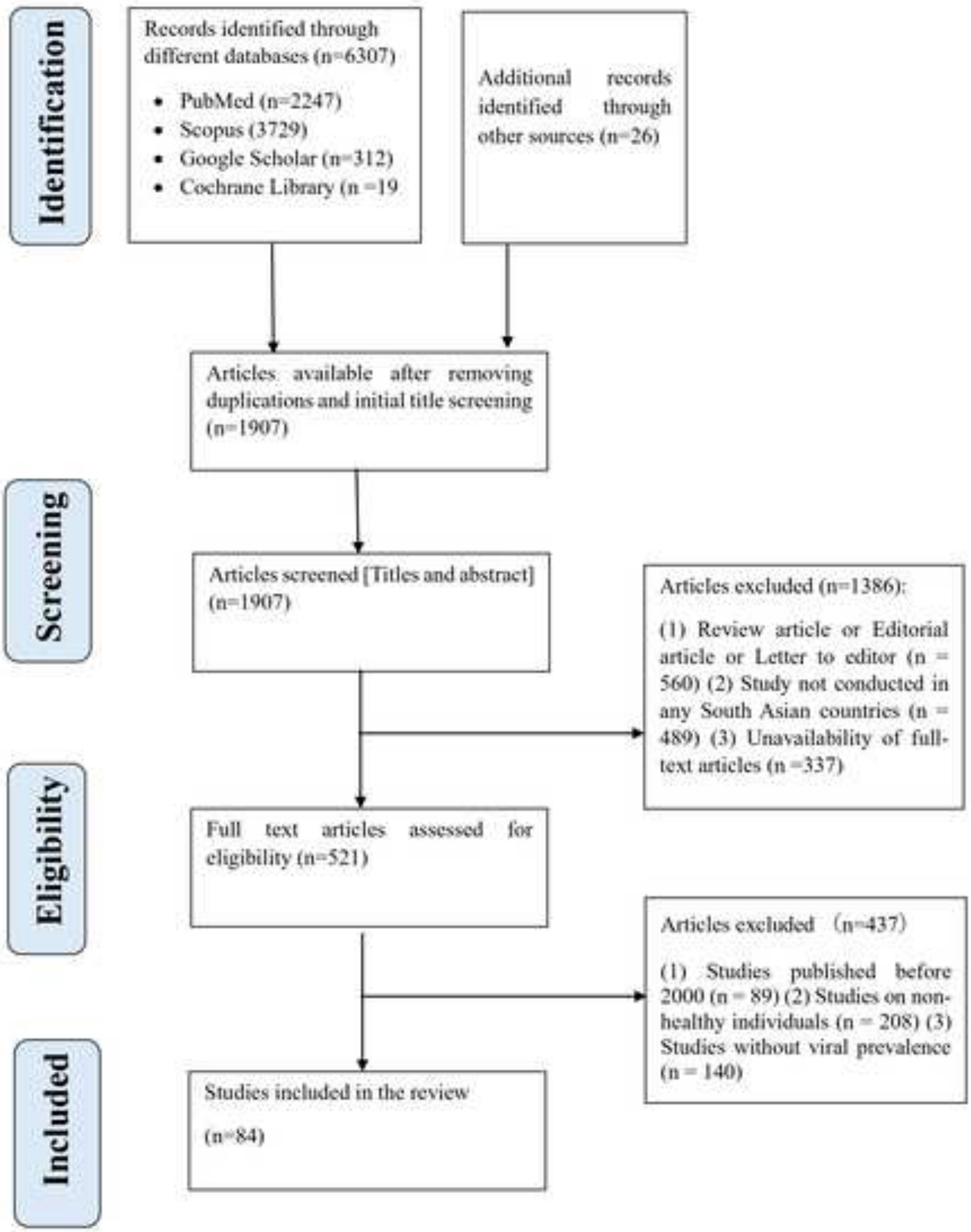

Click here to access/download;Table;Table 1. Characteristics of the selected studies (1).docx

Table 1: Characteristics of the selected studies

Reference	Country	Study Design	Virus Name	Testing Method	Sample Size	Prevalence			
						CMV	EBV	HPV	HSV
Schneider et al – 2010 [50]	India	Cross-sectional	HSV	ELISA	12617				947
Ray et al – 2008 [51]	India	Case-control	HSV	ELISA	801				50
Sgaier et al – 2015 [52]	India	Cross-sectional	HSV	ELISA	1848				187
Dave et al – 2012 [53]	India	Cross-sectional	HSV	ELISA	841				29
Ghosh et al – 2019 [54]	India	Cross-sectional	HSV, HPV, CMV, EBV	PCR	2240	1417	1444	620	21
Adamson et al – 2011 [55]	India	Cross-sectional	HSV	ELISA	897				103
Panchanadeswaran et al – 2006 [56]	India	Cross-sectional	HSV	ELISA	1620				214
Schensul et al – 2007 [57]	India	Cross-sectional	HSV	ELISA	641				62
Patil et al – 2020 [58]	India	Case-control	HSV	ELISA	125				10
Banandur et al – 2011 [59]	India	Not given	HSV	ELISA	12180				1413
Mir et al – 2009 [60]	Pakistan	Cross-sectional	HSV	ELISA	2383				83
Johnson et al – 2014 [61]	Nepal	Cross-sectional	HPV	APTIMA HPV assay	261			25	
Sharmin et al – 2021 [62]	Bangladesh	Cohort	HPV	PCR	410			121	
Nahar et al – 2014 [63]	Bangladesh	Cohort	HPV	PCR	1902			145	
Parwez et al – 2022 [64]	India	Cross-sectional	HPV	PCR	154			19	
Sureshkumar et al – 2015 [65]	India	Cross-sectional	HPV	PCR	1699			179	
Peedicayil et al – 2009 [66]	India	Cross-sectional	HPV	PCR	58			13	
Datta et al – 2010 [67]	India	Not given	HPV	PCR	1300			145	
Dutta et al – 2012 [68]	India	Not given	HPV	PCR	2313			212	
Sherpa et al – 2009 [69]	Nepal	Not given	HPV	PCR	898			73	
Thilagavathi et al – 2012 [70]	India	Not given	HPV	PCR	238			22	
Franceschi et al – 2005 [71]	India	Not given	HPV	PCR	1891			252	
Mittal et al – 2015 [72]	India	Not given	HPV	HC2	43313			2055	
Silver et al – 2011 [73]	India	Not given	HPV, EBV, CMV	PCR	464	121	93	93	
Hussain et al – 2012 [74]	India	Not given	HPV	PCR	800			22	
Johnson et al – 2016 [75]	Nepal	Cross-sectional	HPV	APTIMA HPV assay	265			20	
Aziz et al – 2023 [76]	Pakistan	Not given	HPV	PCR	135			6	
Shahid et al – 2015 [77]	Pakistan	Cross-sectional	HPV	PCR	160			17	
Baussano et al – 2017 [78]	Bhutan	Cross-sectional	HPV	careHPV test	2590			265	
Becker et al – 2007 [79]	India	Not given	HSV	ELISA	901				170
Parvez et al – 2023 [80]	India	Cross-sectional	HPV	PCR	1000			36	
Clifford et al – 2023 [81]	Bhutan	Cross-sectional	HPV	careHPV test	2547			260	
Shakya et al – 2018 [82]	Nepal	Not given	HPV	PCR	1289			186	
Todd et al – 2012 [83]	Afghanistan	Cross-sectional	HSV	ELISA	4750				144
Ramesh et al – 2021 [84]	India	Cross-sectional	HPV	PCR	200			0	
Subramanian et al – 2021 [85]	India	Cross-sectional	HPV	HC2	1523			193	
Dakshinamurthy et al – 2023 [86]	India	Not given	HPV	PCR	1035			270	
Mishra et al – 2022 [87]	India	Cross-sectional	HPV	PCR	217			12	

Shashidhar et al – 2021 [88]	India	Cross-sectional	HPV	PCR	126			9	
Bhattacharya et al – 2018 [89]	India	Not given	HPV	PCR	684			252	
Asiaf et al – 2012 [90]	India	Not given	HPV	PCR	210			29	
Sauvaget et al – 2011 [91]	India	Cross-sectional	HPV	HC2	27192			2812	
Sharma et al – 2015 [92]	India	Cohort	HPV	PCR	2034			262	
Srivastava et al – 2012 [93]	India	Not given	HPV	PCR	2424			240	
Mapitigama et al – 2023 [94]	Sri Lanka	Cross-sectional	HPV	PCR	1012			56	
Vinodhini et al – 2012 [95]	India	Case-control	HPV	PCR	257			78	
Khanna et al – 2009 [96]	India	Not given	HPV	Southern blot	45			9	
Naushad et al – 2017 [97]	Pakistan	Not given	HPV, EBV	PCR	100		0	0	
Gunasekera et al – 2015 [98]	Sri Lanka	Case-control	HPV	ELISA	51			7	
Saranath et al – 2001 [99]	India	Not given	HPV	PCR	164			62	
Gopalkrishna et al – 2000 [100]	India	Not given	HPV	PCR	30			4	
Pandit et al – 2013 [101]	India	Not given	EBV	ELISA	140		138		
Lourembam et al – 2015 [102]	India	Case-control	EBV	PCR	115		24		
Janani et al – 2015 [103]	India	Cross-sectional	EBV, HSV, CMV	PCR	60		8		
Janani et al – 2015 [104]	India	Cross-sectional	EBV, CMV	ELISA, PCR	60		35		
Sinha et al – 2015 [105]	India	Cross-sectional	EBV	PCR	30		0		
Ghosh et al – 2014 [106]	India	Case-control	EBV	PCR	100		54		
Noorali et al – 2004 [107]	Pakistan	Not given	EBV	PCR	30		0		
Borthakur et al – 2016 [108]	India	Not given	EBV	IHC	20		0		
Chatterjee et al – 2022 [109]	India	Cross-sectional	EBV	PCR	130		14		
Sangam et al – 2019 [110]	India	Case-control	EBV	PCR	50		8		
Sachithanandham et al – 2013 [111]	India	Not given	EBV, CMV	PCR	70		9		0
Reddy et al – 2016 [112]	India	Case-control	EBV	IHC	25		2		
Sharma et al – 2019 [113]	India	Case-control	EBV, HPV	PCR	150		14	11	
Rizvi et al – 2011 [114]	India	Not given	EBV, CMV	ELISA	30	1	0		
Husseini et al – 2019 [115]	Afghanistan	Not given	CMV	CLIA	485	484			
Chakravarti et al – 2010 [116]	India	Not given	CMV	ELISA	60	3			
Das et al – 2014 [117]	India	Cross-sectional	CMV	ELISA	2100	2070			
Chaudhari et al – 2009 [1181]	India	Cross-sectional	CMV	ELISA	431	379			
Surpam et al – 2005 [119]	India	Not given	CMV, HSV	ELISA	75	1			3
Tewari et al – 2011 [120]	India	Case-control	CMV	ELISA	200	130			
Dubey et al – 2020 [121]	India	Case-control	CMV	ELISA, PCR	42	40			
Anuradha et al – 2011 [122]	India	Not given	CMV	ELISA	52	6			
Mujtaba et al – 2001 [123]	India	Not given	CMV	ELISA, PCR	32	1			
Kothari et al – 2002 [124]	India	Not given	CMV	ELISA	200	190			
Kumar et al – 2008 [125]	India	Cross-sectional	CMV	ELISA	5600	4			
Sharma et al – 2007 [126]	India	Not given	CMV	ELISA	25	25			
Padmavati et al – 2012 [127]	India	Not given	CMV	ELISA	200	159			
Thapa et al – 2018 [128]	Nepal	Cross-sectional	HPV	PCR	998			197	
Perera et al – 2021 [129]	Sri Lanka	Cross-sectional	HPV	PCR	822			51	

Gibney et al – 2001 [130]	Bangladesh	Cross-sectional	HSV	ELISA	387					100
Ibrahim et al – 2016 [131]	Pakistan	Cross-sectional	CMV	ELISA	217	205				
Hawkes et al – 2002 [132]	Bangladesh	Cross-sectional	HSV	ELISA	312					18
Munir et al – 2023 [133]	Pakistan	Cross-sectional	EBV	PCR	100		9			

Abbreviations: *ELISA* Enzyme-linked Immunosorbent Assay, *PCR* Polymerase Chain Reaction, *HC2* Hybrid-Capture 2, *IHC* Immunohistochemistry, *CLIA* Chemiluminescence Immunoassay.

Click here to access/download;Table;S1 Table. Searching detail, MeSH terms.docx

S1 Table: Searching detail, MeSH terms, and alternative terms used in this study

PubMed	Scopus	Cochrane	Google Scholar
((CMV[Title/Abstract]) AND (Seroprevalence[Title/Abstract])) AND (Afghanistan[Title/Abstract])<br>((CMV[Title/Abstract]) AND (Seroprevalence[Title/Abstract])) AND (Sri Lanka[Title/Abstract])<br>((CMV[Title/Abstract]) AND (Seroprevalence[Title/Abstract])) AND (Nepal[Title/Abstract])<br>((CMV[Title/Abstract]) AND (Seroprevalence[Title/Abstract])) AND (Bhutan[Title/Abstract])<br>((CMV[Title/Abstract]) AND (Seroprevalence[Title/Abstract])) AND (Maldives[Title/Abstract])<br>((CMV[Title/Abstract]) AND (Seroprevalence[Title/Abstract])) AND (Pakistan[Title/Abstract])<br>((CMV[Title/Abstract]) AND (Seroprevalence[Title/Abstract])) AND (India[Title/Abstract])<br>((CMV[Title/Abstract]) AND (Seroprevalence[Title/Abstract])) AND (Bangladesh[Title/Abstract])<br>((CMV[Title/Abstract]) AND (seroprevalence[Title/Abstract])) AND (South Asia[Title/Abstract])<br>"Incidence"[Title/Abstract] AND "Epstein-Barr"[Title/Abstract] AND "Afghanistan"[Title/Abstract]<br>"Incidence"[Title/Abstract] AND "Epstein-Barr"[Title/Abstract] AND "Maldives"[Title/Abstract]<br>"Incidence"[Title/Abstract] AND "Epstein-Barr"[Title/Abstract] AND "Bhutan"[Title/Abstract]<br>"Incidence"[Title/Abstract] AND "Epstein-Barr"[Title/Abstract] AND "Nepal"[Title/Abstract]<br>"Incidence"[Title/Abstract] AND "Epstein-Barr"[Title/Abstract] AND "Nepal"[Title/Abstract]<br>"Incidence"[Title/Abstract] AND "Epstein-Barr"[Title/Abstract] AND "sri lanka"[Title/Abstract]<br>"Incidence"[Title/Abstract] AND "Epstein-Barr"[Title/Abstract] AND "Pakistan"[Title/Abstract]<br>"Incidence"[Title/Abstract] AND "Epstein-Barr"[Title/Abstract] AND "India"[Title/Abstract]<br>"Incidence"[Title/Abstract] AND "Epstein-Barr"[Title/Abstract] AND "Bangladesh"[Title/Abstract]	(TITLE-ABS-KEY (seroprevalence) AND TITLE-ABS-KEY (cytomegalovirus) AND TITLE-ABS-KEY (india))<br>(TITLE-ABS-KEY (seroprevalence) AND TITLE-ABS-KEY (cytomegalovirus) AND TITLE-ABS-KEY (bangladesh))<br>(TITLE-ABS-KEY (seroprevalence) AND TITLE-ABS-KEY (cytomegalovirus) AND TITLE-ABS-KEY (pakistan))<br>(TITLE-ABS-KEY (seroprevalence) AND TITLE-ABS-KEY (cytomegalovirus) AND TITLE-ABS-KEY (nepal))<br>(TITLE-ABS-KEY (seroprevalence) AND TITLE-ABS-KEY (cytomegalovirus) AND TITLE-ABS-KEY (sri AND lanka))<br>(TITLE-ABS-KEY (seroprevalence) AND TITLE-ABS-KEY (cytomegalovirus) AND TITLE-ABS-KEY (afghanistan))<br>(TITLE-ABS-KEY (seroprevalence) AND TITLE-ABS-KEY (cytomegalovirus) AND TITLE-ABS-KEY (maldives))<br>(TITLE-ABS-KEY (seroprevalence) AND TITLE-ABS-KEY (cytomegalovirus) AND TITLE-ABS-KEY (bhutan))<br>(TITLE-ABS-KEY (seroprevalence) AND TITLE-ABS-KEY (cytomegalovirus) AND TITLE-ABS-KEY (south AND asia))<br>(TITLE-ABS-KEY (prevalence) AND TITLE-ABS-KEY (epstein-barr AND virus) AND TITLE-ABS-KEY (south AND asia))<br>(TITLE-ABS-KEY (prevalence) AND TITLE-ABS-KEY (epstein-barr AND virus) AND TITLE-ABS-KEY (bangladesh))<br>(TITLE-ABS-KEY (prevalence) AND TITLE-ABS-KEY (epstein-barr AND virus) AND TITLE-ABS-KEY (india))<br>(TITLE-ABS-KEY (prevalence) AND TITLE-ABS-KEY (epstein-barr AND virus) AND TITLE-ABS-KEY (pakistan))<br>(TITLE-ABS-KEY (prevalence) AND TITLE-ABS-KEY (epstein-barr AND virus) AND TITLE-ABS-KEY (sri AND lanka))<br>(TITLE-ABS-KEY (prevalence) AND TITLE-ABS-KEY (epstein-barr AND virus) AND TITLE-ABS-KEY (nepal))	("Epstein Barr virus"):ti,ab,kw AND ("pregnant"):ti,ab,kw AND ("South Asia"):ti,ab,kw (Word variations have been searched)<br>("Epstein Barr virus"):ti,ab,kw AND ("pregnant women"):ti,ab,kw AND ("South Asia"):ti,ab,kw (Word variations have been searched)<br>("Epstein Barr virus"):ti,ab,kw AND ("pregnant women"):ti,ab,kw AND ("Bangladesh"):ti,ab,kw (Word variations have been searched)<br>("Epstein Barr virus"):ti,ab,kw AND ("pregnant women"):ti,ab,kw AND ("India"):ti,ab,kw (Word variations have been searched)<br>("Epstein Barr virus"):ti,ab,kw AND ("pregnant women"):ti,ab,kw AND ("Pakistan"):ti,ab,kw (Word variations have been searched)<br>("Epstein Barr virus"):ti,ab,kw AND ("pregnant women"):ti,ab,kw AND ("Sri Lanka"):ti,ab,kw (Word variations have been searched)<br>("Epstein Barr virus"):ti,ab,kw AND ("pregnant women"):ti,ab,kw AND ("Nepal"):ti,ab,kw (Word variations have been searched)<br>("Epstein Barr virus"):ti,ab,kw AND ("pregnant women"):ti,ab,kw AND ("Bhutan"):ti,ab,kw (Word variations have been searched)<br>("Epstein Barr virus"):ti,ab,kw AND ("pregnant women"):ti,ab,kw AND ("Maldives"):ti,ab,kw (Word variations have been searched)<br>("Epstein Barr virus"):ti,ab,kw AND ("pregnant women"):ti,ab,kw AND ("Afghanistan"):ti,ab,kw (Word variations have been searched)<br><br>("EBV"):ti,ab,kw AND ("pregnant women"):ti,ab,kw AND ("South Asia"):ti,ab,kw (Word variations have been searched)<br>("EBV"):ti,ab,kw AND ("pregnant women"):ti,ab,kw AND ("Bangladesh"):ti,ab,kw (Word variations have been searched)<br>("EBV"):ti,ab,kw AND ("pregnant women"):ti,ab,kw AND ("India"):ti,ab,kw (Word variations have been searched)<br>("EBV"):ti,ab,kw AND ("pregnant women"):ti,ab,kw AND ("Pakistan"):ti,ab,kw (Word variations have been searched)<br>("EBV"):ti,ab,kw AND ("pregnant women"):ti,ab,kw AND ("Sri Lanka"):ti,ab,kw (Word variations have been searched)	((“Prevalence”) OR (“Seroprevalence”)) AND ((Cytomegalovirus) OR (CMV)) AND (healthy adults) AND (South Asia)<br>((“Prevalence”) OR (“Seroprevalence”)) AND ((Epstein-Barr virus) OR (EBV)) AND (healthy adults) AND (South Asia)<br>((“Prevalence”) OR (“Seroprevalence”)) AND ((Human papilloma virus) OR (HPV)) AND (healthy adults) AND (South Asia)<br>((“Prevalence”) OR (“Seroprevalence”)) AND ((Herpes Simplex virus) OR (HSV)) AND (healthy adults) AND (South Asia)<br>((“Prevalence”) OR (“Seroprevalence”)) AND ((Cytomegalovirus) OR (CMV)) AND (healthy adults) AND (Bangladesh)<br>((“Prevalence”) OR (“Seroprevalence”)) AND ((Epstein-Barr virus) OR (EBV)) AND (healthy adults) AND (India)<br>((“Prevalence”) OR (“Seroprevalence”)) AND ((Human papilloma virus) OR (HPV)) AND (healthy adults) AND (Pakistan)<br>((“Prevalence”) OR (“Seroprevalence”)) AND ((Herpes Simplex virus) OR (HSV)) AND (healthy adults) AND (Sri lanka)<br>((“Prevalence”) OR (“Seroprevalence”)) AND ((Cytomegalovirus) OR (CMV)) AND (healthy adults) AND (Nepal)<br>((“Prevalence”) OR (“Seroprevalence”)) AND ((Epstein-Barr virus) OR (EBV)) AND (healthy adults) AND (Bhutan)<br>((“Prevalence”) OR (“Seroprevalence”)) AND ((Human papilloma virus) OR (HPV)) AND (healthy adults) AND (Afghanistan)<br>((“Prevalence”) OR (“Seroprevalence”)) AND ((Herpes Simplex virus) OR (HSV)) AND (healthy adults) AND (Maldives)

"Incidence"[Title/Abstract] AND "Epstein-Barr"[Title/Abstract] AND "south asia"[Title/Abstract]<br>"Seroprevalence"[Title/Abstract] AND "Epstein-Barr"[Title/Abstract] AND "Afghanistan"[Title/Abstract]<br>"Seroprevalence"[Title/Abstract] AND "Epstein-Barr"[Title/Abstract] AND "Maldives"[Title/Abstract]<br>"Seroprevalence"[Title/Abstract] AND "Epstein-Barr"[Title/Abstract] AND "Bhutan"[Title/Abstract]<br>"Seroprevalence"[Title/Abstract] AND "Epstein-Barr"[Title/Abstract] AND "Nepal"[Title/Abstract]<br>"Seroprevalence"[Title/Abstract] AND "Epstein-Barr"[Title/Abstract] AND "sri lanka"[Title/Abstract]<br>"Seroprevalence"[Title/Abstract] AND "Epstein-Barr"[Title/Abstract] AND "Pakistan"[Title/Abstract]<br>"Seroprevalence"[Title/Abstract] AND "Epstein-Barr"[Title/Abstract] AND "India"[Title/Abstract]<br>"Seroprevalence"[Title/Abstract] AND "Epstein-Barr"[Title/Abstract] AND "Bangladesh"[Title/Abstract]<br>"Seroprevalence"[Title/Abstract] AND "Epstein-Barr"[Title/Abstract] AND "south asia"[Title/Abstract]<br>"Seroprevalence"[Title/Abstract] AND "HPV"[Title/Abstract] AND "Afghanistan"[Title/Abstract]<br>"Seroprevalence"[Title/Abstract] AND "HPV"[Title/Abstract] AND "Maldives"[Title/Abstract]<br>"Seroprevalence"[Title/Abstract] AND "HPV"[Title/Abstract] AND "Bhutan"[Title/Abstract]<br>"Seroprevalence"[Title/Abstract] AND "HPV"[Title/Abstract] AND "Myanmar"[Title/Abstract]<br>"Seroprevalence"[Title/Abstract] AND "HPV"[Title/Abstract] AND "Pakistan"[Title/Abstract]<br>"Seroprevalence"[Title/Abstract] AND "HPV"[Title/Abstract] AND "Nepal"[Title/Abstract]<br>"Seroprevalence"[Title/Abstract] AND "HPV"[Title/Abstract] AND "sri lanka"[Title/Abstract]<br>"Seroprevalence"[Title/Abstract] AND "HPV"[Title/Abstract] AND "India"[Title/Abstract]<br>"Seroprevalence"[Title/Abstract] AND "HPV"[Title/Abstract] AND "Bangladesh"[Title/Abstract]<br>"Seroprevalence"[Title/Abstract] AND "HPV"[Title/Abstract] AND "south asia"[Title/Abstract]<br>"Prevalence"[Title/Abstract] AND "hsv 1"[Title/Abstract] AND "Afghanistan"[Title/Abstract]<br>"Prevalence"[Title/Abstract] AND "hsv 1"[Title/Abstract] AND "Maldives"[Title/Abstract]<br>"Prevalence"[Title/Abstract] AND "hsv 1"[Title/Abstract] AND "Bhutan"[Title/Abstract]	(TITLE-ABS-KEY (prevalence) AND TITLE-ABS-KEY (epstein-barr AND virus) AND TITLE-ABS-KEY (bhutan))<br>(TITLE-ABS-KEY (prevalence) AND TITLE-ABS-KEY (epstein-barr AND virus) AND TITLE-ABS-KEY (maldives))<br>(TITLE-ABS-KEY (prevalence) AND TITLE-ABS-KEY (epstein-barr AND virus) AND TITLE-ABS-KEY (afghanistan))<br>(TITLE-ABS-KEY (prevalence) AND TITLE-ABS-KEY (hpv) AND TITLE-ABS-KEY (south AND asia))<br>(TITLE-ABS-KEY (prevalence) AND TITLE-ABS-KEY (hpv) AND TITLE-ABS-KEY (bangladesh))<br>(TITLE-ABS-KEY (prevalence) AND TITLE-ABS-KEY (hpv) AND TITLE-ABS-KEY (india))<br>(TITLE-ABS-KEY (prevalence) AND TITLE-ABS-KEY (hpv) AND TITLE-ABS-KEY (sri AND lanka))<br>(TITLE-ABS-KEY (prevalence) AND TITLE-ABS-KEY (hpv) AND TITLE-ABS-KEY (nepal))<br>(TITLE-ABS-KEY (prevalence) AND TITLE-ABS-KEY (hpv) AND TITLE-ABS-KEY (pakistan))<br>(TITLE-ABS-KEY (prevalence) AND TITLE-ABS-KEY (hpv) AND TITLE-ABS-KEY (myanmar))<br>(TITLE-ABS-KEY (prevalence) AND TITLE-ABS-KEY (hpv) AND TITLE-ABS-KEY (bhutan))<br>(TITLE-ABS-KEY (prevalence) AND TITLE-ABS-KEY (hpv) AND TITLE-ABS-KEY (maldives))<br>(TITLE-ABS-KEY (prevalence) AND TITLE-ABS-KEY (hpv) AND TITLE-ABS-KEY (afghanistan))<br>(TITLE-ABS-KEY (hsv 1) AND TITLE-ABS-KEY (seroprevalence) AND TITLE-ABS-KEY (south AND asia))<br>(TITLE-ABS-KEY (hsv 1) AND TITLE-ABS-KEY (seroprevalence) AND TITLE-ABS-KEY (bangladesh))<br>(TITLE-ABS-KEY (hsv 1) AND TITLE-ABS-KEY (seroprevalence) AND TITLE-ABS-KEY (india))<br>(TITLE-ABS-KEY (hsv 1) AND TITLE-ABS-KEY (seroprevalence) AND TITLE-ABS-KEY (sri AND lanka))<br>(TITLE-ABS-KEY (hsv 1) AND TITLE-ABS-KEY (seroprevalence) AND TITLE-ABS-KEY (nepal))<br>(TITLE-ABS-KEY (hsv 1) AND TITLE-ABS-KEY (seroprevalence) AND TITLE-ABS-KEY (pakistan))<br>(TITLE-ABS-KEY (hsv 1) AND TITLE-ABS-KEY (seroprevalence) AND TITLE-ABS-KEY (myanmar))<br>(TITLE-ABS-KEY (hsv 1) AND TITLE-ABS-KEY (seroprevalence) AND TITLE-ABS-K(TITLE-ABS-KEY (incidence) AND TITLE-ABS-KEY (hsv) AND TITLE-ABS-KEY (india))	("EBV"):ti,ab,kw AND ("pregnant women"):ti,ab,kw AND ("nepal"):ti,ab,kw (Word variations have been searched)<br>("EBV"):ti,ab,kw AND ("pregnant women"):ti,ab,kw AND ("Bhutan"):ti,ab,kw (Word variations have been searched)<br>("EBV"):ti,ab,kw AND ("pregnant women"):ti,ab,kw AND ("Maldives"):ti,ab,kw (Word variations have been searched)<br>("EBV"):ti,ab,kw AND ("pregnant women"):ti,ab,kw AND ("Afghanistan"):ti,ab,kw (Word variations have been searched)<br><br>("Epstein Barr virus"):ti,ab,kw AND ("Seroprevalence"):ti,ab,kw AND ("South Asia"):ti,ab,kw (Word variations have been searched)<br>("Epstein Barr virus"):ti,ab,kw AND ("Seroprevalence"):ti,ab,kw AND ("Bangladesh"):ti,ab,kw (Word variations have been searched)<br>("Epstein Barr virus"):ti,ab,kw AND ("Seroprevalence"):ti,ab,kw AND ("India"):ti,ab,kw (Word variations have been searched)<br>("Epstein Barr virus"):ti,ab,kw AND ("Seroprevalence"):ti,ab,kw AND ("Pakistan"):ti,ab,kw (Word variations have been searched)<br>("Epstein Barr virus"):ti,ab,kw AND ("Seroprevalence"):ti,ab,kw AND ("Sri Lanka"):ti,ab,kw (Word variations have been searched)<br>("Epstein Barr virus"):ti,ab,kw AND ("Seroprevalence"):ti,ab,kw AND ("Nepal"):ti,ab,kw (Word variations have been searched)<br>("Epstein Barr virus"):ti,ab,kw AND ("Seroprevalence"):ti,ab,kw AND ("Bhutan"):ti,ab,kw (Word variations have been searched)<br>("Epstein Barr virus"):ti,ab,kw AND ("Seroprevalence"):ti,ab,kw AND ("Maldives"):ti,ab,kw (Word variations have been searched)<br>("Epstein Barr virus"):ti,ab,kw AND ("Seroprevalence"):ti,ab,kw AND ("Afghanistan"):ti,ab,kw (Word variations have been searched)<br>(Seroprevalence):ti,ab,kw AND (HSV):ti,ab,kw AND (South Asia):ti,ab,kw (Word variations have been searched)<br>(Seroprevalence):ti,ab,kw AND (HSV):ti,ab,kw AND (Bangladesh):ti,ab,kw (Word variations have been searched)<br>(Seroprevalence):ti,ab,kw AND (HSV):ti,ab,kw AND (India):ti,ab,kw (Word variations have been searched)	

"Prevalence"[Title/Abstract] AND "hsv 1"[Title/Abstract] AND "Myanmar"[Title/Abstract]<br>"Prevalence"[Title/Abstract] AND "hsv 1"[Title/Abstract] AND "Pakistan"[Title/Abstract]<br>"Prevalence"[Title/Abstract] AND "hsv 1"[Title/Abstract] AND "Nepal"[Title/Abstract]<br>"Prevalence"[Title/Abstract] AND "hsv 1"[Title/Abstract] AND "Bangladesh"[Title/Abstract]<br>"Prevalence"[Title/Abstract] AND "hsv 1"[Title/Abstract] AND "India"[Title/Abstract]<br>"Prevalence"[Title/Abstract] AND "hsv 1"[Title/Abstract] AND "Sri Lanka"[Title/Abstract]<br>"Prevalence"[Title/Abstract] AND "hsv 1"[Title/Abstract] AND "South Asia"[Title/Abstract]<br>"Frequency"[Title/Abstract] AND "herpes simplex virus"[Title/Abstract] AND "Afghanistan"[Title/Abstract]<br>"Frequency"[Title/Abstract] AND "herpes simplex virus"[Title/Abstract] AND "Maldives"[Title/Abstract]<br>"Frequency"[Title/Abstract] AND "herpes simplex virus"[Title/Abstract] AND "Bhutan"[Title/Abstract]<br>"Frequency"[Title/Abstract] AND "herpes simplex virus"[Title/Abstract] AND "Myanmar"[Title/Abstract]<br>"Frequency"[Title/Abstract] AND "herpes simplex virus"[Title/Abstract] AND "Pakistan"[Title/Abstract]<br>"Frequency"[Title/Abstract] AND "herpes simplex virus"[Title/Abstract] AND "Nepal"[Title/Abstract]<br>"Frequency"[Title/Abstract] AND "herpes simplex virus"[Title/Abstract] AND "sri lanka"[Title/Abstract]<br>"Frequency"[Title/Abstract] AND "herpes simplex virus"[Title/Abstract] AND "India"[Title/Abstract]<br>"Frequency"[Title/Abstract] AND "herpes simplex virus"[Title/Abstract] AND "Bangladesh"[Title/Abstract]<br>itle/Abstract] AND "herpes simplex virus"[Title/Abstract]<br>sia"[Title/Abstract]	(TITLE-ABS-KEY (incidence) AND TITLE-ABS-KEY (hsv) AND TITLE-ABS-KEY (south AND asia))<br>(TITLE-ABS-KEY (incidence) AND TITLE-ABS-KEY (hsv) AND TITLE-ABS-KEY (bangladesh))<br>(TITLE-ABS-KEY (incidence) AND TITLE-ABS-KEY (hsv) AND TITLE-ABS-KEY (sri AND lanka))<br>(TITLE-ABS-KEY (incidence) AND TITLE-ABS-KEY (hsv) AND TITLE-ABS-KEY (nepal))<br>(TITLE-ABS-KEY (incidence) AND TITLE-ABS-KEY (hsv) AND TITLE-ABS-KEY (pakistan))<br>(TITLE-ABS-KEY (incidence) AND TITLE-ABS-KEY (hsv) AND TITLE-ABS-KEY (myanmar))<br>(TITLE-ABS-KEY (incidence) AND TITLE-ABS-KEY (hsv) AND TITLE-ABS-KEY (bhutan))<br>(TITLE-ABS-KEY (incidence) AND TITLE-ABS-KEY (hsv) AND TITLE-ABS-KEY (maldives))<br>(TITLE-ABS-KEY (incidence) AND TITLE-ABS-KEY (hsv) AND TITLE-ABS-KEY (afghanistan))EY (afghanistan))	(Seroprevalence):ti,ab,kw AND (HSV):ti,ab,kw AND (Sri Lanka):ti,ab,kw (Word variations have been searched)<br>(Seroprevalence):ti,ab,kw AND (HSV):ti,ab,kw AND (Nepal):ti,ab,kw (Word variations have been searched)<br>(Seroprevalence):ti,ab,kw AND (HSV):ti,ab,kw AND (Pakistan):ti,ab,kw (Word variations have been searched)<br>(Seroprevalence):ti,ab,kw AND (HSV):ti,ab,kw AND (Bhutan):ti,ab,kw (Word variations have been searched)<br>(Seroprevalence):ti,ab,kw AND (HSV):ti,ab,kw AND (Maldives):ti,ab,kw (Word variations have been searched)<br>(Seroprevalence):ti,ab,kw AND (HSV):ti,ab,kw AND (Afghanistan):ti,ab,kw (Word variations have been searched)<br><br>(Seroprevalence):ti,ab,kw AND (HPV):ti,ab,kw AND (South Asia):ti,ab,kw (Word variations have been searched)<br>(Seroprevalence):ti,ab,kw AND (HPV):ti,ab,kw AND (Bangladesh):ti,ab,kw (Word variations have been searched)<br>(Seroprevalence):ti,ab,kw AND (HPV):ti,ab,kw AND (India):ti,ab,kw (Word variations have been searched)<br>(Seroprevalence):ti,ab,kw AND (HPV):ti,ab,kw AND (Sri Lanka):ti,ab,kw (Word variations have been searched)<br>(Seroprevalence):ti,ab,kw AND (HPV):ti,ab,kw AND (Nepal):ti,ab,kw (Word variations have been searched)<br>(Seroprevalence):ti,ab,kw AND (HPV):ti,ab,kw AND (Pakistan):ti,ab,kw (Word variations have been searched)<br>(Seroprevalence):ti,ab,kw AND (HPV):ti,ab,kw AND (Bhutan):ti,ab,kw (Word variations have been searched)<br>(Seroprevalence):ti,ab,kw AND (HPV):ti,ab,kw AND (Maldives):ti,ab,kw (Word variations have been searched)<br>(Seroprevalence):ti,ab,kw AND (HPV):ti,ab,kw AND (Afghanistan):ti,ab,kw (Word variations have been searched)	

Click here to access/download;Table;S2 Table. Characteristics of the selected studies.docx

S2 Table: Characteristics of the selected studies

Reference	Study Location	Study Setting	Study Period	Sample type
Schneider et al – 2010 [50]	Andhra Pradesh	Community-based	September 2004 and September 2005	Blood
Ray et al – 2008 [51]	Delhi	Hospital-based	March 2002 - April 2004	Blood
Sgaier et al – 2015 [52]	Hyderabad, Bangalore, Chandigarh	Community-based	2001-2006	Blood
Dave et al – 2012 [53]	Surat city	Community-based	2005	Blood
Ghosh et al – 2019 [54]	Southern coastal Karnataka	Community-based	Not given	Cervical specimen
Adamson et al – 2011 [55]	Mysore	Hospital-based	November 2005 - March 2006	Blood
Panchanadeswaran et al – 2006 [56]	Chennai, Tamil Nadu	Community-based	March and June 2001	Blood
Schensul et al – 2007 [57]	Mumbai	Community-based	June - September 2003	Blood
Patil et al – 2020 [58]	Uttar Pradesh	Community-based	November 2017 - April 2018	Blood
Banandur et al – 2011 [59]	Karnataka	Community-based	Between 2006 and 2008	Blood
Mir et al – 2009 [60]	Pakistan	Community-based	2007	Blood
Johnson et al – 2014 [61]	Western Nepal	Community-based	2013	Cervical specimen
Sharmin et al – 2021 [62]	Bangladesh	Hospital-based	February 2015 and June 2018	Cervical specimen
Nahar et al – 2014 [63]	Dhaka	Community-based	July and December 2011	Cervical specimen
Parwez et al – 2022 [64]	Bihar	Community-based	Not given	Urine
Sureshkumar et al – 2015 [65]	India	Community-based	January 2007 and December 2009	Urine
Peedicayil et al – 2009 [66]	India	Hospital-based	Not given	Cervical specimen
Datta et al – 2010 [67]	New Delhi	Community-based	Not given	Cervical specimen
Dutta et al – 2012 [68]	Eastern India	Community-based	September 2007 - March 2010	Cervical specimen
Sherpa et al – 2009 [69]	Nepal	Community-based	October 2006 and March 2007	Cervical specimen
Thilagavathi et al – 2012 [70]	Tamilnadu	Community-based	August 2009 and July 2010	Urine
Franceschi et al – 2005 [71]	India	Community-based	February and October 2003	Cervical specimen
Mittal et al – 2015 [72]	Kolkata	Community-based	July 2010 and March 2015	Cervical specimen
Silver et al – 2011 [73]	Hydrabad	Community-based	Not given	Cervical specimen
Hussain et al – 2012 [74]	India	Community-based	Not given	Urine
Johnson et al – 2016 [75]	Nepal	Community-based	July, 2013	Cervical specimen
Aziz et al – 2023 [76]	Pakistan	Hospital-based	March 2017 and August 2019	Cervical specimen
Shahid et al – 2015 [77]	Pakistan	Hospital-based	April 2012 - December 2012	Cervical specimen
Baussano et al – 2017 [78]	Bhutan	Community-based	April-May 2016	Cervical specimen
Becker et al – 2007 [79]	India	Community-based	April-September 2003	Blood
Parvez et al – 2023 [80]	India	Community-based	Not given	Cervical specimen
Clifford et al – 2023 [81]	Bhutan	Community-based	April - May 2016	Cervical specimen
Shakya et al – 2018 [82]	Nepal	Community-based	February 2012 - May 2013	Cervical specimen
Todd et al – 2012 [83]	Kabul	Community-based	February 2010 and January 2011	Blood
Ramesh et al – 2021 [84]	India	Hospital-based	March 2019 - December 2019	Oral specimen
Subramanian et al – 2021 [85]	India	Community-based	February 2009 - February 2014,	Cervical specimen
Dakshinamurthy et al – 2023 [86]	India	Hospital-based	Not given	Cervical specimen
Mishra et al – 2022 [87]	India	Hospital-based	May 2019 - April 2021	Cervical specimen
Shashidhar et al – 2021 [88]	India	Hospital-based	Not given	Cervical specimen
Bhattacharya et al – 2018 [89]	India	Hospital-based	Not given	Cervical specimen

Asiaf et al – 2012 [90]	Kashmir	Hospital-based	May 2010 and April 2011	Cervical specimen
Sauvaget et al – 2011 [91]	India	Community-based	2000 January	Cervical specimen
Sharma et al – 2015 [92]	India	Community-based	March-December 2012	Urine
Srivastava et al – 2012 [93]	India	Community-based	October 2005 - December 2010	Cervical specimen
Mapitigama et al – 2023 [94]	Sri Lanka	Hospital-based	February 2019 - July 2019	Cervical specimen
Vinodhini et al – 2012 [95]	Tamil Nadu	Hospital-based	May 2009 and May 2011	Cervical specimen
Khanna et al – 2009 [96]	India	Hospital-based	2 years	Normal mucosa
Naushad et al – 2017 [97]	Pakistan	Hospital-based	2012-2014	Blood
Gunasekera et al – 2015 [98]	Sri Lanka	Hospital-based	Not given	Blood
Saranath et al – 2001 [99]	India	Hospital-based	Not given	Cervical specimen
Gopalkrishna et al – 2000 [100]	New Delhi	Hospital-based	Not given	Cervical specimen
Pandit et al – 2013 [101]	Mumbai, Mangalore	Hospital-based	Not given	Blood
Lourembam et al – 2015 [102]	Northeastern India	Hospital-based	April 2011 - November 2013	Blood
Janani et al – 2015 [103]	Chennai	Hospital-based	Not given	Blood
Janani et al – 2015 [104]	Chennai	Hospital-based	August 2010 - July 2013	Blood
Sinha et al – 2015 [105]	South India	Hospital-based	10 months	Blood
Ghosh et al – 2014 [106]	Northeast India	Hospital-based	Not given	Oral/Blood/Tissue
Noorali et al – 2004 [107]	Karachi	Hospital-based	January 01, 1992 - December 31, 2002	Blood
Borthakur et al – 2016 [108]	Northeast India	Hospital-based	Not given	Tissue
Chatterjee et al – 2022 [109]	North-Eastern India	Hospital-based	May 2014 - April 2019	Blood
Sangam et al – 2019 [110]	India	Hospital-based	Not given	Blood
Sachithanandham et al – 2013 [111]	South India	Hospital-based	Not given	Blood
Reddy et al – 2016 [112]	South India	Hospital-based	Not given	Tissue
Sharma et al – 2019 [113]	South India	Hospital-based	Not given	Oral specimen
Rizvi et al – 2011 [114]	North India	Hospital-based	June 2009 - March 2010	Blood
Husseini et al – 2019 [115]	Main provinces	Community-based	Not given	Blood
Chakravarti et al – 2010 [116]	Delhi	Hospital-based	October 2007 - April 2009.	Blood
Das et al – 2014 [117]	Chandigarh	Community-based	January 2011 - July 2012	Blood
Chaudhari et al – 2009 [1181]	India	Hospital-based	Not given	Blood
Surpam et al – 2005 [119]	India	Not given	Not given	Blood
Tewari et al – 2011 [120]	North India	Hospital-based	2 years	Blood
Dubey et al – 2020 [121]	Mumbai	Hospital-based	Not given	Blood
Anuradha et al – 2011 [122]	Andhra Pradesh	Hospital-based	6 months	Blood
Mujtaba et al – 2001 [123]	Chandigarh	Hospital-based	1997-1999	Blood
Kothari et al – 2002 [124]	Delhi	Hospital-based	4 days	Blood
Kumar et al – 2008 [125]	India	Hospital-based	Not given	Blood
Sharma et al – 2007 [126]	Guwahati	Hospital-based	1 year	Blood
Padmavati et al – 2012 [127]	Delhi	Hospital-based	2000-2004	Blood
Thapa et al – 2018 [128]	Jumla	Community-based	May 2016 - January 2017	Cervical specimen
Perera et al – 2021 [129]	Kalutara	Community-based	1 July 2018 - 30 November 2018	Cervical specimen
Gibney et al – 2001 [130]	Tejgaon	Community-based	October 1998 - April 1999	Blood
Ibrahim et al – 2016 [131]	Karachi	Community-based	July 2010 - June 2012	Blood
Hawkes et al – 2002 [132]	Matlab	Community-based	August 1995	Blood
Munir et al – 2023 [133]	Khyber Pakhtunkhwa	Hospital-based	21 July 2022, 19 August 2023	Blood

Click here to access/download;Table;S3 Table. Quality assessment of selected studies.docx

S3 Table: Quality assessment of selected studies (Q1, Q2, Q3......Q9 denotes nine parameters described by Joanna Briggs Institute Prevalence Critical Appraisal Tool)

Reference	Q1	Q2	Q3	Q4	Q5	Q6	Q7	Q8	Q9	Total Score	Risk
Schneider et al – 2010 [50]	1	1	1	1	1	1	1	1	1	9	Low
Ray et al – 2008 [51]	1	0	1	1	1	1	1	1	1	8	Low
Sgaier et al – 2015 [52]	1	1	1	1	1	1	1	1	1	9	Low
Dave et al – 2012 [53]	0	1	1	1	1	1	1	1	1	8	Low
Ghosh et al – 2019 [54]	1	1	1	1	1	1	1	1	1	9	Low
Adamson et al – 2011 [55]	1	1	1	1	1	1	1	1	1	9	Low
Panchanadeswaran et al – 2006 [56]	1	1	1	1	1	1	1	1	1	9	Low
Schensul et al – 2007 [57]	1	1	1	1	1	1	1	1	1	9	Low
Patil et al – 2020 [58]	1	1	1	1	1	1	1	1	1	9	Low
Banandur et al – 2011 [59]	1	1	1	1	1	1	1	1	1	9	Low
Mir et al – 2009 [60]	1	1	1	1	1	1	1	1	1	9	Low
Johnson et al – 2014 [61]	1	1	1	1	1	1	1	1	0	8	Low
Sharmin et al – 2021 [62]	1	0	1	1	1	1	1	1	1	8	Low
Nahar et al – 2014 [63]	1	1	1	1	1	1	1	1	1	9	Low
Parwez et al – 2022 [64]	1	0	1	1	1	1	1	1	1	8	Low
Sureshkumar et al – 2015 [65]	1	1	1	1	1	1	1	1	1	9	Low
Peedicayil et al – 2009 [66]	1	1	0	1	1	1	1	1	1	8	Low
Datta et al – 2010 [67]	1	1	1	1	1	1	1	1	1	9	Low
Dutta et al – 2012 [68]	1	0	1	1	1	1	1	1	1	8	Low
Sherpa et al – 2009 [69]	1	1	1	1	1	1	1	1	1	9	Low
Thilagavathi et al – 2012 [70]	0	1	1	1	1	1	1	0	0	6	Moderate
Franceschi et al – 2005 [71]	1	1	1	1	1	1	1	1	1	9	Low
Mittal et al – 2015 [72]	1	1	1	1	1	1	1	1	0	8	Low
Silver et al – 2011 [73]	1	0	1	1	1	1	1	1	0	7	Moderate
Hussain et al – 2012 [74]	1	0	1	1	1	1	1	1	1	8	Low
Johnson et al – 2016 [75]	1	1	1	1	1	1	1	1	1	9	Low
Aziz et al – 2023 [76]	0	0	1	1	1	1	1	1	0	6	Moderate
Shahid et al – 2015 [77]	0	1	1	1	1	1	1	1	1	8	Low
Baussano et al – 2017 [78]	1	1	1	1	1	1	1	1	0	8	Low
Becker et al – 2007 [79]	1	1	1	1	1	1	1	1	1	9	Low
Parvez et al – 2023 [80]	1	1	1	1	1	1	1	1	1	9	Low
Clifford et al – 2023 [81]	1	1	1	1	1	1	1	1	1	9	Low
Shakya et al – 2018 [82]	1	1	1	1	1	1	1	1	1	9	Low
Todd et al – 2012 [83]	1	0	1	1	1	1	1	1	1	8	Low
Ramesh et al – 2021 [84]	1	1	1	1	1	1	1	1	1	9	Low
Subramanian et al – 2021 [85]	1	1	1	1	1	1	1	1	1	9	Low
Dakshinamurthy et al – 2023 [86]	1	0	1	1	1	1	1	1	1	8	Low
Mishra et al – 2022 [87]	1	0	1	1	1	1	1	1	1	8	Low
Shashidhar et al – 2021 [88]	1	0	0	1	1	1	1	1	1	7	Moderate
Bhattacharya et al – 2018 [89]	1	1	1	1	1	1	1	1	1	9	Low
Asiaf et al – 2012 [90]	1	0	1	1	1	1	1	1	1	8	Low
Sauvaget et al – 2011 [91]	1	1	1	1	1	1	1	1	1	9	Low
Sharma et al – 2015 [92]	1	1	1	1	1	1	1	1	1	9	Low

Srivastava et al – 2012 [93]	1	1	1	1	1	1	1	1	1	9	Low
Mapitigama et al – 2023 [94]	1	1	1	1	1	1	1	1	1	9	Low
Vinodhini et al – 2012 [95]	1	0	1	1	1	1	1	1	1	8	Low
Khanna et al – 2009 [96]	0	0	0	1	1	1	1	1	1	6	Moderate
Naushad et al – 2017 [97]	1	0	1	1	1	1	1	1	1	8	Low
Gunasekera et al – 2015 [98]	1	0	0	1	1	1	1	1	1	7	Moderate
Saranath et al – 2001 [99]	0	0	1	1	1	1	1	1	1	7	Moderate
Gopalkrishna et al – 2000 [100]	1	0	0	1	1	1	1	1	1	7	Moderate
Pandit et al – 2013 [101]	1	1	1	1	1	1	1	1	1	9	Low
Lourembam et al – 2015 [102]	1	1	1	1	1	1	1	1	1	9	Low
Janani et al – 2015 [103]	1	0	0	1	1	1	1	1	1	7	Moderate
Janani et al – 2015 [104]	0	0	1	1	1	1	1	1	1	7	Moderate
Sinha et al – 2015 [105]	0	1	1	1	1	1	1	0	1	7	Moderate
Ghosh et al – 2014 [106]	1	0	1	1	1	1	1	1	1	8	Low
Noorali et al – 2004 [107]	1	0	0	1	1	1	1	0	1	6	Moderate
Borthakur et al – 2016 [108]	1	0	0	1	1	1	1	1	1	7	Moderate
Chatterjee et al – 2022 [109]	1	1	1	1	1	1	1	1	1	9	Low
Sangam et al – 2019 [110]	0	0	0	1	1	1	1	1	1	6	Moderate
Sachithanandham et al – 2013 [111]	0	1	1	1	1	1	1	1	1	8	Low
Reddy et al – 2016 [112]	0	0	0	1	1	1	1	0	1	5	High
Sharma et al – 2019 [113]	1	1	1	1	1	1	1	1	1	9	Low
Rizvi et al – 2011 [114]	1	0	1	1	1	1	1	0	1	7	Moderate
Husseini et al – 2019 [115]	1	1	1	1	1	1	1	0	0	7	Moderate
Chakravarti et al – 2010 [116]	1	0	0	1	1	1	1	0	1	6	Moderate
Das et al – 2014 [117]	0	0	1	1	1	1	1	1	1	7	Moderate
Chaudhari et al – 2009 [1181]	0	0	1	1	1	1	1	1	0	6	Moderate
Surpam et al – 2005 [119]	0	0	1	0	1	1	1	0	1	5	High
Tewari et al – 2011 [120]	0	1	1	1	1	1	1	1	1	8	Low
Dubey et al – 2020 [121]	0	1	0	1	1	1	1	1	1	7	Moderate
Anuradha et al – 2011 [122]	0	0	0	1	1	1	1	1	1	6	Moderate
Mujtaba et al – 2001 [123]	1	0	0	1	1	1	1	1	0	6	Moderate
Kothari et al – 2002 [124]	1	0	1	1	1	1	1	1	0	7	Moderate
Kumar et al – 2008 [125]	0	0	1	1	1	1	1	1	0	6	Moderate
Sharma et al – 2007 [126]	1	1	0	1	1	1	1	0	1	7	Moderate
Padmavati et al – 2012 [127]	1	0	1	1	1	1	1	1	1	8	Low
Thapa et al – 2018 [128]	1	1	1	1	1	1	1	1	1	9	Low
Perera et al – 2021 [129]	1	1	1	1	1	1	1	1	1	9	Low
Gibney et al – 2001 [130]	1	1	1	1	1	1	1	1	1	9	Low
Ibrahim et al – 2016 [131]	1	1	1	1	1	1	1	1	1	9	Low
Hawkes et al – 2002 [132]	1	1	1	1	1	1	1	1	1	9	Low
Munir et al – 2023 [133]	1	1	1	1	1	1	1	1	1	9	Low

Click here to access/download
Supporting Information
PRISMA_2020_checklist.docx
