# Supplementary material for: Prevalence of CMV, EBV, HPV, and HSV among South Asian healthy population: A systematic review and meta-analysis
Source: PLOS Glob Public Health. 2026 Jan 7;6(1):e0005728. doi: 10.1371/journal.pgph.0005728 (PMC12779128; doi:10.1371/journal.pgph.0005728)
Supplement: Attachment — Submitted filename: Response to Reviewers.docx [file pgph.0005728.s015.docx]

| **Reviewer Comments** | **Author Response** |
| --- | --- |
| **General Comments – Reviewer 1** |  |
| 1. Major concerns regarding the inclusion and exclusion criteria need to be addressed, as they appear overly restrictive. | Thank you for your concern. We have revised the inclusion and exclusion criteria to ensure they are more appropriate and clearly defined. The updates have been made in response to your specific comments, and we have addressed all points of confusion accordingly. |
| 2. Figures are of poor resolution and must be improved for publication-quality presentation. | We have updated all figures to high-resolution versions to ensure they meet publication-quality standards. Thank you. |
| **General Comments – Reviewer 2** |  |
| The authors should be commended for their effort in coming up with this paper. I have found the document to be technically sound, although it would benefit from extensive review of typographical and grammatical/language errors some of which I have highlighted in the attached revised article. | Thank you for your kind words and constructive feedback. We sincerely appreciate your acknowledgement of our efforts and your careful review of the manuscript. We have thoroughly addressed the typographical and grammatical errors you highlighted and have made the necessary corrections throughout the revised version. |
| **Specific Comments – Reviewer 1** |  |
| **Abstract** |  |
| **Lines 12-13:** Remove the specified sentence as it does not add valuable information and does not fit the context of the abstract. | We have revised the sentence to ensure it adds value and aligns more appropriately with the context of the abstract. |
| **Line 30:** Revise the unclear and misleading sentence, “Healthy individuals are infected with at least one of the four DNA viruses.” This implies that most or all healthy individuals are infected, which contradicts the overall pooled prevalence reported in the review. | Thank you for your observation. The sentence has been revised to more accurately reflect the findings and is now phrased in a way that aligns with the reported pooled prevalence, ensuring clarity and precision (Line# 29-30) |
| **Introduction:** |  |
| **Lines 59-60:** Add a reference to this sentence. | Thank you for pointing this out. Regarding sentences (Line# 59–60), we have ensured that the relevant references were already included in the subsequent sentences (Line# 60–64) that support the statement. However, to enhance clarity and avoid any confusion, we have now added the appropriate references directly to the initial sentence as well. |
| **Line 75:** Ensure consistent wording for autism throughout the manuscript. Choose either “autism” or “autism spectrum disorders.” | Suggestion accepted. |
| **Lines 84-87:** Adjust the formatting for virus names and prevalence values. Avoid placing virus names in parentheses while simultaneously using parentheses for prevalence values. Maintain consistent formatting to reduce confusion. | Thank you for addressing out this inconsistency. The formatting of virus names and prevalence values has been adjusted to reduce confusion. |
| **Lines 92-93:** Add a reference to this sentence. | Reference added. |
| **Lines 94-95:** Avoid using “Indian” and “Bangladeshi.” Instead, use phrases like “among individuals in India” or “among individuals in Bangladesh”. | Suggestion accepted. |
| **Methods:** |  |
| **Line 111:** Add “as shown in S1 Table” to index this table in the text. | Suggestion accepted. |
| **Line 116:** Clarify how the three co-authors screened the retrieved records. Specify whether records were divided among them or if each record was screened by at least two reviewers. Provide additional details regarding the title/abstract screening process and the subsequent full-text screening. | Thank you for your helpful comments. We have clarified the screening process in the revised manuscript. We have specified that all retrieved records were independently screened by at least two of the three reviewers (RTS, ZAS, and NMN) during both the title/abstract and full-text screening stages, with discrepancies resolved through discussion or consultation with other co-authors. #Line 118-120 |
| **Lines 117-118:** Revise this unclear sentence. The statement about conducting a search over four months from November 2023 to December 2024 does not make sense and should be clarified. | Thank you for highlighting this inconsistency. The intended meaning was that the search for relevant articles was carried out during that period and continued up until the manuscript submission. However, to avoid any confusion, we have removed the specific time frame and revised the sentence accordingly. Line#121-122 |
| **Line 119:** Since the search is almost a year old, update it to include the most recent studies. | Suggestion accepted and the most recent studies have been included. (Number of new studies included = 10; References #134-143) |
| **Line 127 and S1 Table:** Clarify whether MeSH terms were used, as it currently appears that only keywords were utilized. | Thank you for highlighting this issue. We have used only keywords for this study, and both the sentence and the table have been revised accordingly. |
| **Line 129:** Avoid repeating the full forms of CDC and WHO as they were already introduced earlier. Use the abbreviations instead. | Suggestion accepted. |
| **Lines 128-130:** Provide details about accessing the list of suggested similar articles. Specify whether this was done through CDC and WHO websites. Indicate the number of relevant articles retrieved and include any links, keywords, and the detailed screening methods. | Thank you for your valuable comment. We went through the CDC and WHO websites for additional relevant articles. However, we found that the articles retrieved from these sources were already captured in our main search across the four databases (PubMed, Scopus, Cochrane Library and Google Scholar). Since no new studies were identified, we revised our manuscript to avoid confusion. Line# 115–118 |
| **Line 113:** Address whether grey literature was systematically searched. | Thank you for your comment. Yes, we conducted a systematic search of grey literature, including institutional and organizational websites such as OpenGrey, WHO, CDC, and (NIHR) However, this search did not yield any additional articles that met our inclusion criteria. Therefore, no grey literature was included in the final analysis. |
| **Line 133 and Lines 142-144:** Provide a justification for restricting the search to full-text articles, as prevalence data and population classifications are frequently reported in abstracts, especially in studies conducted in South Asian populations. | Thank you for your thoughtful observation.  Our primary objective was to extract detailed prevalence data of CMV, EBV, HPV, and HSV specifically among healthy individuals who lived in South Asia. While conducting an initial search, we observe that some basic prevalence estimates may appear in abstracts, crucial methodological details such as participant selection criteria, diagnostic methods, and population classification are typically only available in the full text. In this regard, we restrict the search to full-text articles only as access to these details was necessary to ensure that the included studies met our eligibility criteria and to maintain the accuracy and reliability of our findings. |
| **Lines 134-136:** Justify the exclusion of RCTs, considering that these studies can report baseline prevalence data, which is directly relevant to the objectives of this review. | Again, thank you for your observation.  Although, RCTs may include baseline prevalence data, we excluded them because from our initial searching, we observed that in RCTs, the main goal is usually to test an intervention, and the participants are often a very specific group, which can make the data less representative. Also, the baseline data in RCTs may not be detailed enough for our purposes. To keep our review consistent and reliable, we omit RCTs. |
| **Line 138:** Explain why studies before 2000 were excluded. Provide an epidemiological or methodological justification. | Thank you for your comment. Studies published before 2000 were excluded because of significant socio-economic and public health changes that have occurred in South Asia since then. Over the past two decades, improvements in healthcare infrastructure, vaccination programs, hygiene, education, and disease surveillance have likely influenced the epidemiology of viral infections in the region. Including older studies could introduce outdated prevalence estimates that do not reflect current conditions. To ensure that our findings are relevant and accurately represent the present-day burden of these infections, we limited our review to studies published from 2000 onward. |
| **Line 140:** Justify the exclusion of letters to editors, as they may contain relevant primary data on prevalence. | Thank you for raising this point.  We exclude them for the same reason we include only full-text articles in our review as they often lack critical methodological details such as sample size, study design, and population characteristics which are essential to maintain the accuracy and reliability of our findings. |
| **Line 131:** Define “healthy population” as used in this review. | In this review, the term ‘healthy population’ refers to individuals who do not have any chronic medical conditions or serious health issues that could influence immune function or viral susceptibility. This includes people with minor or self-limiting illnesses (e.g., common cold) but excludes those with chronic diseases (such as cancer, HIV, diabetes, cardiovascular disease and others), immunocompromised status, or conditions requiring long-term medical care. The aim was to focus on individuals who are not affected by underlying health conditions that might skew the prevalence of viral infections. |
| **Line 150:** Include the initials of the three co-authors who extracted data. Describe whether each study was extracted three times or divided among the co-authors to ensure double extraction. | Thank you for your suggestion. The eligible studies were divided among the three co-authors (RTS, ZAS, and NMN), with each author independently extracting data from their assigned studies. To ensure accuracy and consistency, the extracted data were then cross-checked by another co-author (BB). The sentences have been revised in the updated manuscript (Line# 150-152) |
| **Line 152:** Define what is meant by “population” in the context of study settings. | In this review, “population” refers to the group of individuals from whom data were collected for each of our selected study articles. This includes the demographic and geographic characteristics that define the participants, such as age, health status, and location (e.g., community-based residents, blood donors, or specific subgroups within South Asian countries). The term encompasses all participants who met the study’s inclusion criteria and from whom prevalence data were obtained. |
| **Line 168:** Specify whether any data transformation was performed to stabilize variance before pooling prevalence data. If not, justify this decision. | Thank you for your insightful comment. We used the DerSimonian-Laird random-effects model, which is widely applied in meta-analyses for estimating pooled prevalence and accounts for heterogeneity across studies. No data transformation was performed to stabilize variance before pooling the prevalence data, as the model itself accounts for variability by incorporating study-specific variances. Given this, additional transformations were not deemed necessary, as the model adequately handles the heterogeneity and variance stabilization. |
| **Line 170:** Specify the type of random-effects model used (e.g., DerSimonian-Laird, Bayesian). | Thank you so much for addressing this point; we used the DerSimonian-Laird random-effects model. In the updated version of our manuscript, we addressed it. Line# 174 |
| **Results:** |  |
| **Figure 1:** Ensure the PRISMA flowchart follows the original published template, as the current version appears to be a modified and non-standard representation. Provide a more detailed description of the “other sources” section, including the inclusion and exclusion process. Carefully check for typos (e.g., inconsistent use of parentheses and n=). | We appreciate the suggestion, and the PRISMA flowchart has been revised accordingly to follow the original published template. |
| **Table 1:** The numbers presented in the prevalence column are not percentages and do not accurately represent prevalence. Please clarify this, as the current format is incorrect and highly confusing. | Again, thank you so much for your effort in reviewing every detail of our manuscript. We understand that the prevalence column in Table 1 may create some confusion. Therefore, we replace the word “Prevalence” with “Total number of infected individuals,” which more precisely represents the number of individuals infected with at least one of the four viruses (CMV, EBV, HPV, HSV) across the selected studies. |
| **Lines 183-184:** Explain how more than half of the population is classified as “hospital-based” if the inclusion criterion is healthy populations. | Thank you for pointing out this confusion. More than half of the population was classified as “hospital-based” because many of the healthy individuals included in our review were either control groups in hospital-based studies or patients who visited hospitals for routine health checkups. Although these participants were recruited in hospital settings, they did not have chronic illnesses or conditions that would exclude them from the definition of a healthy population used in our study. To leave no confusion, we revised the sentence (Line #186-187) |
| **Line 185:** It is not necessarily required for the study design to be explicitly stated; it can often be identified from the described methodology. Please clarify why these studies were classified as having an unknown study design. | We specifically classified the study design only when it was explicitly mentioned in the article. Although some aspects of the methodology might suggest the design, we chose not to infer or assume the study design to maintain accuracy and avoid misclassification. Therefore, studies without a clear statement of their design were classified as “Not given” |
| **Lines 187-188:** Include references for the studies mentioned. | Suggestion accepted. |
| **Line 194:** Revise the vague term “A maximum number.” | Suggestion accepted. |
| **Line 197:** Index S2 Table in the text by stating “in Table 1 and S2 Table.” | Suggestion accepted. |
| **Line 257:** Correct the indexing error; supplementary figure 5 is for the forest plot of HSV among males, while the results of the quality assessment are in supplementary table 3. | Thank you for pointing out this honest mistake. The indexing errors have been corrected. |
