# Supplementary material for: Prevalence of CMV, EBV, HPV, and HSV among South Asian healthy population: A systematic review and meta-analysis
Source: PLOS Glob Public Health. 2026 Jan 7;6(1):e0005728. doi: 10.1371/journal.pgph.0005728 (PMC12779128; doi:10.1371/journal.pgph.0005728)
Supplement: Attachment — Submitted filename: Authors response to reviewer comments.docx [file pgph.0005728.s017.docx]

| **Reviewers Comments** | **Author Response** |
| --- | --- |
| Concerns previously raised regarding how some figures (i.e. Roman Numerals) were written, and the need for extensive language editing, have not yet been addressed by the authors in the revised manuscript R1. | Thank you for your feedback. We have carefully re-examined the entire manuscript, including all figures, and can confirm that no figures contain Roman numerals. Additionally, extensive language editing was conducted in Manuscript R1, with particular attention to clarity and readability. We believe that the current version accurately conveys our findings, and further editing may risk altering the intended meaning of key points. |
| There are a number of concerns remaining over the reporting of the methodology. Your previous response indicated that only keyword searches were utilised, but the manuscript still refers to MeSH terms. In addition, justification of the inclusion and exclusion criteria, as well as the search period, are missing from the manuscript. | Thank you for these constructive comments. Upon careful review, we confirm that neither the main manuscript nor the supplementary materials contain any references to MeSH terms. Only keyword searches were utilized in our study.  Justification of the the inclusion and exclusion criteria has been added in the manuscript. **(#L135-L154)**  Also, we have added the search period in the revised manuscript. The initial search was conducted between January and March 2024, with an updated search carried out in April–May 2025. The retrieved studies covered the period from 2000 to 2025. **(#L117-L120)** |
